# Thermodynamic Evaluation and Optimization of the (NaCl + Na_2_CO_3_ + Na_2_SO_4_ + Na_2_S_2_O_7_ + Na_2_CrO_4_ + Na_2_Cr_2_O_7_ + Na_2_O + KCl + K_2_CO_3_ + K_2_SO_4_ + K_2_S_2_O_7_ + K_2_CrO_4_ + K_2_Cr_2_O_7_ + K_2_O) System Involved in High-Temperature Corrosion

**DOI:** 10.1021/acsomega.6c00729

**Published:** 2026-05-19

**Authors:** Sara Benalia, Fiseha Tesfaye, Daniel Lindberg, Leena Hupa, Patrice Chartrand, Christian Robelin

**Affiliations:** b Centre for Research in Computational Thermochemistry (CRCT), Department of Chemical Engineering, 563124 Polytechnique Montréal, 3535 Queen Mary Road, Montréal, Quebec H3V 1H8, Canada; c Johan Gadolin Process Chemistry Centre, Laboratory of Molecular Science and Engineering, Åbo Akademi University, Henrikinkatu 2, Turku FI-20500, Finland; d School of Chemical Engineering, Department of Chemical and Metallurgical Engineering, Aalto University, Aalto FI-00076, Finland

## Abstract

A complete critical evaluation of all available phase diagram and thermodynamic data has been performed for all condensed phases of the salt system (NaCl + Na_2_CO_3_ + Na_2_SO_4_ + Na_2_S_2_O_7_ + Na_2_CrO_4_ + Na_2_Cr_2_O_7_ + Na_2_O + KCl + K_2_CO_3_ + K_2_SO_4_ + K_2_S_2_O_7_ + K_2_CrO_4_ + K_2_Cr_2_O_7_ + K_2_O) (diluted in free oxides), and optimized model parameters have been found. This system is important for the investigation of the chemistry of the different deposits formed in steel and stainless steel installations with Ni, Cr, Mo, W, and V as alloying elements during the combustion processes for energy production. The (NaCl + Na_2_CO_3_ + Na_2_SO_4_ + Na_2_S_2_O_7_ + KCl + K_2_CO_3_ + K_2_SO_4_ + K_2_S_2_O_7_) subsystem has been critically evaluated in previous articles. The Modified Quasichemical Model in the Quadruplet Approximation was used for both the molten salt phase and the high-temperature hexagonal solid solution (Na_2_CO_3_ + Na_2_SO_4_ + Na_2_CrO_4_ + K_2_CO_3_ + K_2_SO_4_ + K_2_CrO_4_) to take into account short-range-order between ions, while the Compound Energy Formalism was used for all other solid solutions. Owing to the lack of data, DSC-TGA experiments were conducted for several compositions in a few common-ion binary subsystems, with emphasis on solid–solid phase equilibria, and for one composition in a common-cation ternary subsystem. Also, the nonstoichiometric chrome-glaserite phase was investigated in particular at the composition (45 mol % Na_2_CrO_4_ + 55 mol % K_2_CrO_4_) using SEM-EDS and DSC-TGA, after annealing at 400 °C for 3 weeks. The model parameters obtained for the binary and ternary subsystems can be used to predict thermodynamic properties and phase equilibria for the multicomponent system.

## Graphical abstract

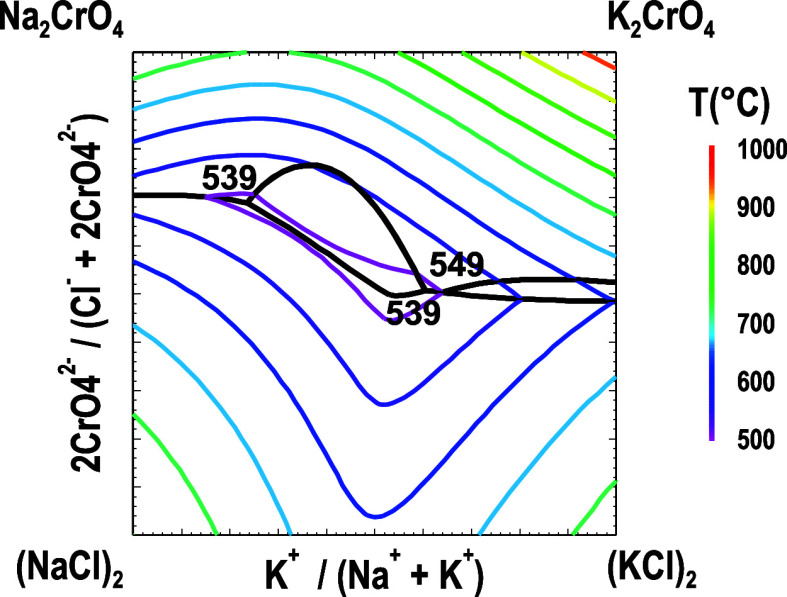

## 1 Introduction

Combustion-based energy production facilities are exposed to high-temperature gases containing impurities like SO_2_, Cl_2_, S_2_, and HCl and corrosive products such as Na_2_O, K_2_O, NaOH, KOH, NaCl, and KCl.^1–3^ These gases are a mixture of N_2_, CO_2_, CO, O_2_, and H_2_O and can contribute to corrosive deposit formation during ash evaporation in combustion. The most common deposits include KCl, NaCl, Na_2_SO_4_, and K_2_CO_3_.^1,3–5^ The presence of elements such as K, Na, Ca, Mg, Fe, Al, Si, P, S, Cl, C, H, and O during operation can also contribute to the formation of ashes and gases, which may cause different operational problems such as corrosion, slagging, fouling, and agglomeration.^6,7^ The extent of corrosiveness depends on the composition of the gas and volatile ash, which in turn is influenced by factors such as the amount of air needed for combustion and the type of fuel used, along with any impurities that may be present.^8^ Corrosion mechanisms are also associated with the nature of the steel or alloy used in combustion plants installation, as well as the formation of low melting point mixtures. Equipment made of steels and stainless steels with Ni, Cr, Mo, W, and V as alloying elements are particularly prone to this problem.^2,9–11^ The corrosion type experienced by the equipment under these conditions is known as hot corrosion. It takes place at elevated temperatures (from 600 to 950 °C)^4,12,13^ and is typically observed in municipal waste incineration, pyrolysis, and gasification of challenging fuels and feedstocks such as biomass waste. The temperature range over which corrosion typically occurs in certain combustion processes, such as black liquor combustion, is around 500 °C. The threshold for such temperatures is determined by the lowest melting temperature of the ashes generated during the process.

According to Li et al.,^14^ various complex reactions can lead to the formation of alkali chromates and alkali dichromates (including Na_2_CrO_4_, K_2_CrO_4_, Na_2_Cr_2_O_7_, and K_2_Cr_2_O_7_), in which chromium has an oxidation state of 6+. Therefore, when an alkali salt deposit forms on the stainless steel surface, the protective Cr_2_O_3_ layer can be partially deteriorated according to the following reactions:

$${Cr}_{2}O_{3}+4NaCl\;\left(\mathrm{or}\;4KCl\right)+\frac{5}{2}O_{2}⇌2{Na}_{2}{CrO}_{4}\;\left(\mathrm{or}\;2K_{2}{CrO}_{4}\right)+2{Cl}_{2}$$

$${Cr}_{2}O_{3}+2NaCl\;\left(\mathrm{or}\;2KCl\right)+2O_{2}⇌{Na}_{2}{Cr}_{2}O_{7}\;\left(\mathrm{or}\;K_{2}{Cr}_{2}O_{7}\right)+{Cl}_{2}$$

$$Cr+2NaCl\;\left(\mathrm{or}\;2KCl\right)+2O_{2}⇌{Na}_{2}{CrO}_{4}\;\left(\mathrm{or}\;K_{2}{CrO}_{4}\right)+{Cl}_{2}$$

Both chromates and chlorine can interact as oxidants. Hence, the degradation kinetics of Cr-based alloys may be established from the following reaction:^14^

$$Cr+{Na}_{2}{CrO}_{4}\;\left(\mathrm{or}\;K_{2}{CrO}_{4}\right)+{Cl}_{2}⇌{Cr}_{2}O_{3}+2NaCl\;\left(\mathrm{or}\;2KCl\right)+\frac{1}{2}O_{2}$$

Typically, pure chromium is significantly more reactive than Cr_2_O_3_ with NaCl (or KCl).^14^

Lindberg et al.^15–17^ developed previously an accurate thermodynamic model for the Na^+^, K^+^//Cl^–^, CO_3_^2–^, SO_4_^2–^, S_2_O_7_^2–^ reciprocal system. The present paper focuses on adding alkali chromates (Na_2_CrO_4_ and K_2_CrO_4_) and alkali dichromates (Na_2_Cr_2_O_7_ and K_2_Cr_2_O_7_) to this existing model. As a first step, an assessment of the thermodynamic properties (standard enthalpy of formation Δ*H*_298.15K_^°^ from the elements in their stable standard state at 298.15K and 1 atm, absolute (third law) entropy *S*_298.15K_^°^ referenced at 298.15K and 1 atm, and heat capacity *C*_P_ as a function of temperature) of Na_2_CrO_4_, K_2_CrO_4_, Na_2_Cr_2_O_7_, and K_2_Cr_2_O_7_ was conducted by us using the available data from the literature and also newly obtained differential scanning calorimetry (DSC) measurements (for Na_2_CrO_4_ and K_2_CrO_4_).^18,19^ In addition, in those studies, extensive information on the crystal structures of all allotropes of the four compounds was collected in order to identify possible solid solutions forming with other considered salts.

The present paper describes a thermodynamic model for the (NaCl + Na_2_CO_3_ + Na_2_SO_4_ + Na_2_S_2_O_7_ + Na_2_CrO_4_ + Na_2_Cr_2_O_7_ + Na_2_O + KCl + K_2_CO_3_ + K_2_SO_4_ + K_2_S_2_O_7_ + K_2_CrO_4_ + K_2_Cr_2_O_7_ + K_2_O) system. The optimization of the (NaCl + Na_2_CO_3_ + Na_2_SO_4_ + Na_2_S_2_O_7_ + KCl + K_2_CO_3_ + K_2_SO_4_ + K_2_S_2_O_7_) subsystem has been presented in previous articles.^15–17^ The free oxides (Na_2_O and K_2_O) are present in dilute amounts in the liquid solution since reactions of the type 2 A_2_CrO_4_ = A_2_Cr_2_O_7_ + A_2_O (where A = Na, K) are very limited up to above the liquidus temperatures.^19^ In this work, owing to the lack of data, differential scanning calorimetry (DSC)–thermogravimetric analysis (TGA) experiments were performed for several compositions in a few common-ion binary subsystems ((Na_2_CO_3_ + Na_2_CrO_4_), (K_2_CO_3_ + K_2_CrO_4_), and (Na_2_CrO_4_ + K_2_CrO_4_)), with emphasis on solid–solid phase equilibria. In addition, the chrome-glaserite solid solution was investigated in particular at the composition (45 mol % Na_2_CrO_4_ + 55 mol % K_2_CrO_4_) after annealing, using both scanning electron microscopy (SEM)–energy-dispersive spectroscopy (EDS) and DSC-TGA. Finally, the common-cation ternary mixture (55.0 mol % KCl + 28.0 mol % K_2_CO_3_ + 17.0 mol % K_2_CrO_4_) (corresponding to the predicted ternary eutectic) was studied by DSC-TGA.

As already mentioned, the present article describes the addition of the chromates and dichromates of sodium and potassium to the thermodynamic model of Lindberg et al. for the Na^+^, K^+^//Cl^–^, CO_3_^2–^, SO_4_^2–^, S_2_O_7_^2–^ system. Later on, the molybdates and dimolybdates of sodium and potassium were added to this new thermodynamic model, and this work has already been published.^20^ The present and previous^20^ studies are therefore closely related. In both cases, the CALPHAD (“CALculation of PHAse Diagrams”) method was used: All relevant experimental thermodynamic information (mainly phase equilibria) was collected from the literature, and the most reliable data were identified (based on the purity of the reagents, the experimental techniques used, etc.) and then used to calibrate the thermodynamic model. In addition, DSC-TGA measurements were conducted for several binary subsystems (for which data were lacking) and SEM-EDS was used to characterize the hP14 glaserite phase (chrome-glaserite in this work, and molybdenum-glaserite in our previously published work^20^). These in-house data were used along with the available data from the literature to calibrate the model. The present work is devoted to CrO_4_- and Cr_2_O_7_-based systems, and later on, it was extended by the addition of the MoO_4_^2–^ and Mo_2_O_7_^2–^ anions.^20^ Thus, these two studies do complement each other, but the present work includes original theoretical and experimental work.

All calculations and optimizations were performed using the FactSage software package.^21^

## 2 Thermodynamic Data, Crystal Structures, and Space Groups for the Pure Compounds

This study used directly the thermodynamic data (Δ*H*_298.15K_^°^, *S*_298.15 K_^°^, and *C*_P_) previously selected for the condensed pure compounds of the (NaCl + Na_2_CO_3_ + Na_2_SO_4_ + Na_2_S_2_O_7_ + Na_2_CrO_4_ + Na_2_Cr_2_O_7_ + Na_2_O + KCl + K_2_CO_3_ + K_2_SO_4_ + K_2_S_2_O_7_ + K_2_CrO_4_ + K_2_Cr_2_O_7_ + K_2_O) system.^15,16,18,19,22^ Chartrand and Pelton^22^ evaluated the thermodynamic data for NaCl and KCl, based on the compilation of Barin et al.^23^ Lindberg et al.^15,16^ assessed the thermodynamic properties of Na_2_CO_3_, Na_2_SO_4_, K_2_CO_3_, K_2_SO_4_, Na_2_S_2_O_7_, and K_2_S_2_O_7_ based on the NIST-JANAF thermodynamic data compilation^24^ and the work of Dessureault et al.^25^

The thermodynamic data for Na_2_CrO_4_, K_2_CrO_4_, Na_2_Cr_2_O_7_, and K_2_Cr_2_O_7_ were directly taken from our previous studies^18,19^ and were based on experimental data available in the literature, and our XRD analysis and DSC-TGA measurements (for Na_2_CrO_4_ and K_2_CrO_4_). Finally, the thermodynamic properties of Na_2_O and K_2_O were taken directly from the FToxid database in FactSage.^21^

Table S1 in the Supporting Information provides details on the crystal structures and space groups of all relevant pure salt compounds. In our previous work, we determined the most likely crystal structures and space groups for the different forms of Na_2_CrO_4_, K_2_CrO_4_, Na_2_Cr_2_O_7_, and K_2_Cr_2_O_7_.^18,19^

## 3 Thermodynamic Models for the Liquid Phase and Solid Solutions

The liquid phase and the high-temperature hexagonal solid solution hP22 in the (NaCl + Na_2_CO_3_ + Na_2_SO_4_ + Na_2_S_2_O_7_ + Na_2_CrO_4_ + Na_2_Cr_2_O_7_ + Na_2_O + KCl + K_2_CO_3_ + K_2_SO_4_ + K_2_S_2_O_7_ + K_2_CrO_4_ + K_2_Cr_2_O_7_ + K_2_O) system (diluted in free oxides) were both modeled using the Modified Quasichemical Model in the Quadruplet Approximation (MQMQA).^26^ Additionally, several low-temperature solid solutions exhibiting limited solubility were considered and modeled using the Compound Energy Formalism (CEF).^27–29^ Detailed descriptions of the MQMQA and CEF can be found, respectively, in Sections 2.1 and 2.2 of the Supporting Information. All optimized model parameters are listed in these two sections.

## 4 Experimental Procedure

### 4.1 Simultaneous DSC-TGA

#### Materials and Measurements

In this study, we used high-purity gases (99.999% pure Ar and 99.999% pure CO_2_) provided by Oy Linde Gas AB (Finland), along with the initial reagents KCl, Na_2_CO_3_, K_2_CO_3_, Na_2_CrO_4_·4H_2_O, and K_2_CrO_4_. Table 1 presents an overview of the suppliers, CAS numbers, and purities of all solid reagents.

**Table 1:** Source and Purity of Solid Reagents

**chemical formula**	**supplier**	**CAS number**	**purity (%)**
KCl	Sigma-Aldrich	7447-40-7	≥99.0
Na_2_CO_3_	Sigma-Aldrich	497-19-8	≥99.95
K_2_CO_3_	Sigma-Aldrich	584-08-7	99.995
Na_2_CrO_4_·4H_2_O	Sigma-Aldrich	10034-82-9	99.0
K_2_CrO_4_	Alfa-Aesar	7789-00-6	99.9

A NETZSCH STA 449 F1 Jupiter simultaneous DSC-TGA apparatus was used to perform experiments in relevant binary subsystems, specifically in (Na_2_CO_3_ + Na_2_CrO_4_), (K_2_CO_3_ + K_2_CrO_4_), and (Na_2_CrO_4_ + K_2_CrO_4_), at compositions for which experimental data (particularly phase diagram data) were not available in the existing literature. In addition, the ternary common-cation mixture (55.0 mol % KCl + 28.0 mol % K_2_CO_3_ + 17.0 mol % K_2_CrO_4_) was investigated to confirm the characteristics of the ternary eutectic predicted by our model.

The calibration of the DSC apparatus is described in ref 18. The estimated experimental uncertainty for temperature measurements was ±1 °C. Before conducting DSC-TGA, a dehydration treatment was performed for each pure compound.

To eliminate the moisture absorbed during storage and a substantial portion of the water originally present in the Na_2_CrO_4_·4H_2_O reagent, all reagents were heated in a Vulcan oven (model: 3-130) at 200 °C for 3 h. Once the treatment was completed, the reagents were allowed to cool down to 25 °C.

For measurements in the two common-cation binary subsystems (Na_2_CO_3_ + Na_2_CrO_4_) and (K_2_CO_3_ + K_2_CrO_4_) and the common-cation ternary subsystem (KCl + K_2_CO_3_ + K_2_CrO_4_), 15–20 mg of the dehydrated binary or ternary mixtures was placed in a Pt/Rh (80/20) crucible. During all experiments, a protective gas mixture consisting of 90 mL/min CO_2_ and 10 mL/min Ar was used. It should be noted that the use of a saturated CO_2_ gas atmosphere was intended to avoid dissociation of Na_2_CO_3_ and K_2_CO_3_ at high temperatures into Na_2_O_(S)_ and CO_2(g)_, and K_2_O_(S)_ and CO_2(g)_ and/or partial volatilization.

During experiments for the (Na_2_CrO_4_ + K_2_CrO_4_) common-anion binary subsystem, an inert atmosphere consisting of a continuous flow of 70 mL/min Ar was maintained. Detailed results for all DSC-TGA experiments are provided in the Supporting Information (Tables S5–S9).

To ensure thermal stability, both the sample and reference were first heated from room temperature to 40 °C and held at this temperature for 10 min. Following this, each sample underwent three consecutive heating–cooling cycles, at a constant rate of 10 °C/min.

The temperature range investigated was carefully selected for each binary or ternary composition. The maximum temperature exceeded the estimated liquidus temperature by 49 °C, whereas the minimum temperature was 50 °C (or lower) below the first solid–solid transition. A 1 min holding time was used at both temperatures. The maximum temperature was carefully controlled to prevent excessive vaporization while guaranteeing complete melting. Thermogravimetric analysis (TGA) was employed to continuously monitor any mass loss or volatilization during the experiments. Both mass loss and heat flow were measured, with a mass loss target of no more than 3%. The overall mass loss for each binary or ternary composition investigated was determined at the end of each DSC-TGA experiment and is reported in the Supporting Information (Tables S5–S9).

Typically, only heating runs were taken into account. Each solid–solid transition was determined as the temperature at the onset of the peak, while each liquidus temperature and eutectic temperature was identified as the temperature at the peak maximum.^30^

For all samples, our subsequent analyses excluded the first heating/cooling cycle owing to the thermal history of the sample. An exception was made for the four chrome-glaserite compositions investigated (Table S8 in the Supporting Information), as the corresponding samples had undergone a 3-week equilibration at 400 °C. That is, all heating/cooling cycles were considered for these four samples.

In this paper, for each binary common-ion subsystem investigated experimentally, a DSC thermogram related to a specific composition is displayed.

To achieve the equilibration of the chrome-glaserite phase, pretreated K_2_CrO_4_ and Na_2_CrO_4_·4H_2_O powders were first mixed in a 55:45 molar ratio (K_2_CrO_4_:Na_2_CrO_4_). In a muffle furnace, this mixture was heated in a fused silica tube to 800 °C at a rate of 15 °C/min for 50 min and then was cooled to 400 °C at the same rate (15 °C/min) for 27 min. Annealing was then conducted at 400 °C for a period of 3 weeks in an Ar atmosphere (flow rate of around 1 mL/min), followed by rapid cooling (quenching) in liquid nitrogen. The sample was then mechanically crushed and analyzed using the SEM-EDS and DSC-TGA techniques.

### 4.2 Scanning Electron Microscopy (SEM) and Energy-Dispersive X-ray Spectroscopy (EDS)

After the quenching process, the equilibrated chrome-glaserite sample (solid solution consisting of 45 mol % Na_2_CrO_4_ and 55 mol % K_2_CrO_4_) was examined at room temperature using a LEO 1450 scanning electron microscope (SEM) (Carl Zeiss Microscopy GmbH, Jena, Germany), coupled with an Oxford Instruments X-Max 50 mm^2^ energy-dispersive spectrometer (EDS) (Oxford Instruments plc, Abingdon, Oxfordshire, UK).

The global homogeneity of the chrome-glaserite phase has been confirmed by SEM-EDS. Twenty EDS analyses were conducted to confirm the formation of this phase. Specific results from six of these analyses are provided in Table 2, with an approximate experimental error of ±5%. The equilibrated chrome-glaserite phase has an approximate composition of Na_1.03_K_1.13_Cr_1.05_O_3.92_, which compares reasonably well to the composition of Na_0.9_K_1.1_CrO_4_ for the original mechanical mixture.

**Table 2:** EDS Analysis Results of Selected Spectra for the Quenched Chrome-Glaserite Sample (Approximate Uncertainty of ±5%)

**element**	**spectrum 1**	**spectrum 2**	**spectrum 5**	**spectrum 6**	**spectrum 12**	**spectrum 17**	**average**	**theoretical value**
**O**	3.847	4.058	3.982	3.890	3.841	3.920	**3.923**	**4**
**Na**	1.000	1.056	1.000	1.000	1.013	1.097	**1.028**	**0.9**
**K**	1.222	1.101	1.093	1.216	1.090	1.075	**1.133**	**1.1**
**Cr**	1.157	1.000	1.008	1.108	1.000	1.000	**1.045**	**1**
**K**/(**Na + K**)	0.550	0.510	0.522	0.549	0.518	0.495	**0.524**	**0.55**
(**Na + K**)/**Cr**	1.920	2.157	2.076	2.000	2.103	2.172	**2.068**	**2**

## 5 Results and Discussion

### 5.1 Chromate-Based Common-Ion Binary Subsystems

The present section discusses all common-ion binary subsystems involving CrO_4_ and Cr_2_O_7_, for which thermodynamic data (mainly phase diagram data) were available in the literature. Phase diagrams were calculated in air (with an oxygen partial pressure of 0.21 atm), with an ideal gas phase consisting of the following gaseous species: C, C_2_, C_3_, C_4_, C_5_, O, O_2_, O_3_, CO, C_2_O, CO_2_, C_3_O_2_, Na, Na_2_, NaO, S, S_2_, S_3_, S_4_, S_5_, S_6_, S_7_, S_8_, CS, CS_2_, SO, SO_2_, SO_3_, SSO, COS, Na_2_SO_4_, Cl, Cl_2_, CCl, C_2_Cl, CCl_2_, C_2_Cl_2_, CCl_3_, C_2_Cl_3_, CCl_4_, C_2_Cl_4_, C_2_Cl_5_, C_2_Cl_6_, C_6_Cl_6_, ClO, ClO_2_, ClO_3_, Cl_2_O, ClOOCl, ClOClO, ClClOO, COCl, COCl_2_, NaCl, (NaCl)_2_, SCl, S_2_Cl, SCl_2_, ClSSCl, SOCl_2_, SO_2_Cl_2_, K, K_2_, KO, K_2_SO_4_, KCl, (KCl)_2_, Cr, CrO, CrO_2_, CrO_3_, CrS, CrCl, CrCl_2_, CrCl_3_, CrCl_4_, CrCl_5_, CrCl_6_, CrOCl, CrO_2_Cl, CrOCl_2_, CrO_2_Cl_2_, CrOCl_3_, and CrOCl_4_ (all taken from the FactPS database in FactSage^21^). The possible reactions 2 A_2_CrO_4_ = A_2_Cr_2_O_7_ + A_2_O (with A = Na, K) were taken into account in our phase diagram calculations for all CrO_4_-based binary subsystems. It is important to note that these reactions are negligible up to above the liquidus temperatures.^19^

#### 5.1.1 The (NaCl + Na_2_CrO_4_) System

Various techniques were used to investigate the phase diagram.^31–33^ Sackur^31^ employed the method of cooling curves to measure the liquidus temperature of four NaCl-rich mixtures. Rasonskaya and Bergman^32^ and Shinata^33^ used the visual-polythermal technique and differential thermal analysis (DTA), respectively. The (NaCl + Na_2_CrO_4_) binary system is of simple eutectic-type; no solid solutions or intermediate compounds have been evidenced. The experimental limiting slopes of the NaCl and Na_2_CrO_4_ liquidus curves close to the pure salts (*x*(NaCl) → 1 and *x*(Na_2_CrO_4_) → 1, respectively) comply with eq 1, which assumes no solid solubility:

$$\left(\frac{dT}{{\mathrm{dx}}_{m}^{\mathrm{liquidus}}}\right)=\frac{RT_{\mathrm{fusion}\left(m\right)}^{2}}{\Delta h_{\mathrm{fusion}\left(m\right)}^{{}^{\circ}}}\;\mathrm{at}\;\left[x_{m}=1\right]$$ 1

where Δ*h*_fusion_^°^ and *T*_fusion_ are, respectively, the enthalpy and temperature of fusion of the pure salt *m*.

The measurements of Shinata^33^ are somewhat scattered. The liquidus data of Rasonskaya and Bergman^32^ are satisfactorily reproduced by our model. Rasonskaya and Bergman^32^ reported a eutectic at 54.4 mol % Na_2_CrO_4_ and 572 °C, whereas Shinata^33^ reported a eutectic temperature of 576.9 °C. The eutectic is calculated at 54.3 mol % Na_2_CrO_4_ and 574 °C.

The thermal arrest observed by Shinata^33^ at about 419 °C corresponds to the solid–solid transition Na_2_CrO_4(S1)_ = Na_2_CrO_4(S2)_.

Figure 1 displays the calculated (NaCl + Na_2_CrO_4_) phase diagram in air (p­(O_2_) = 0.21 atm) along with the available data.

**Figure 1:**
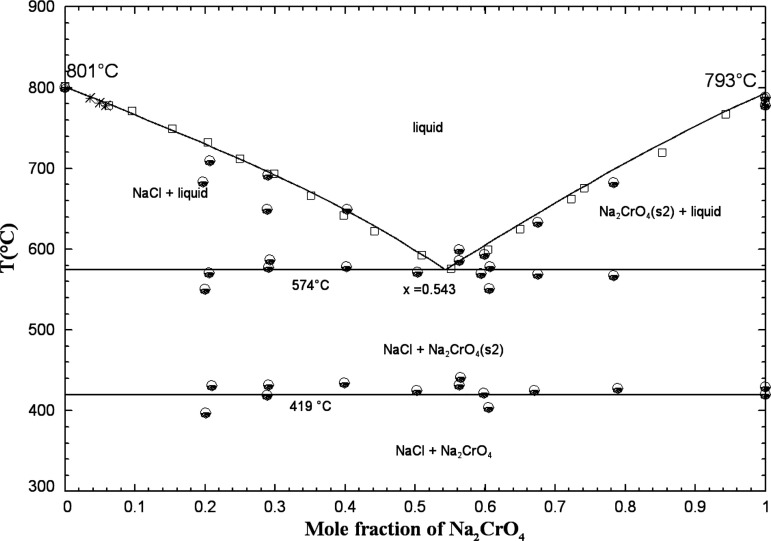
Calculated (NaCl + Na_2_CrO_4_) phase diagram in air (p­(O_2_) = 0.21 atm). Experimental data from Sackur^31^ (asterisk), Rasonskaya and Bergman^32^ (open square), and Shinata^33^ (down-filled circle).

#### 5.1.2 The (KCl + K_2_CrO_4_) System

Both the cooling curve method^31,34^ and the visual-polythermal technique in a platinum crucible^35,36^ have been used to measure the phase diagram. The (KCl + K_2_CrO_4_) binary system is of simple eutectic type; no solid solutions or intermediate compounds have been identified. The experimental limiting slope of the KCl liquidus curve complies with eq 1.

Measurements of liquidus temperatures for KCl-rich mixtures from Rassonskaya and Bergman^35,36^ and Sackur^31^ are consistent with each other. It was not possible to reproduce the data of Żemcżużny^34^ near the pure compounds since their melting points reported by Żemcżużny are higher than the values selected in the present work.^18,21^ In particular, for pure KCl, this author measured a temperature of fusion more than 20 °C higher than the accepted value of 771 °C.

For compositions near the eutectic point, the liquidus temperatures of refs 34 and 35,36 are in reasonable agreement. Żemcżużny^34^ reported a eutectic at 31.5 mol % K_2_CrO_4_ and 658 °C, while Rassonskaya and Bergman^35,36^ reported a somewhat lower eutectic temperature of 650 °C at the same composition. The eutectic is calculated at 32.1 mol % K_2_CrO_4_ and 651 °C.

The binary liquid was assumed to be ideal (that is, Δ*g*_K_2_/(Cl)(CrO_4_)_ = 0). This is consistent with the fact that the (NaCl + Na_2_CrO_4_) liquid is relatively close to ideal (see model parameters in Table S3 of the Supporting Information).

Figure 2 displays the calculated (KCl + K_2_CrO_4_) phase diagram in air (p­(O_2_) = 0.21 atm) along with the available data.

**Figure 2:**
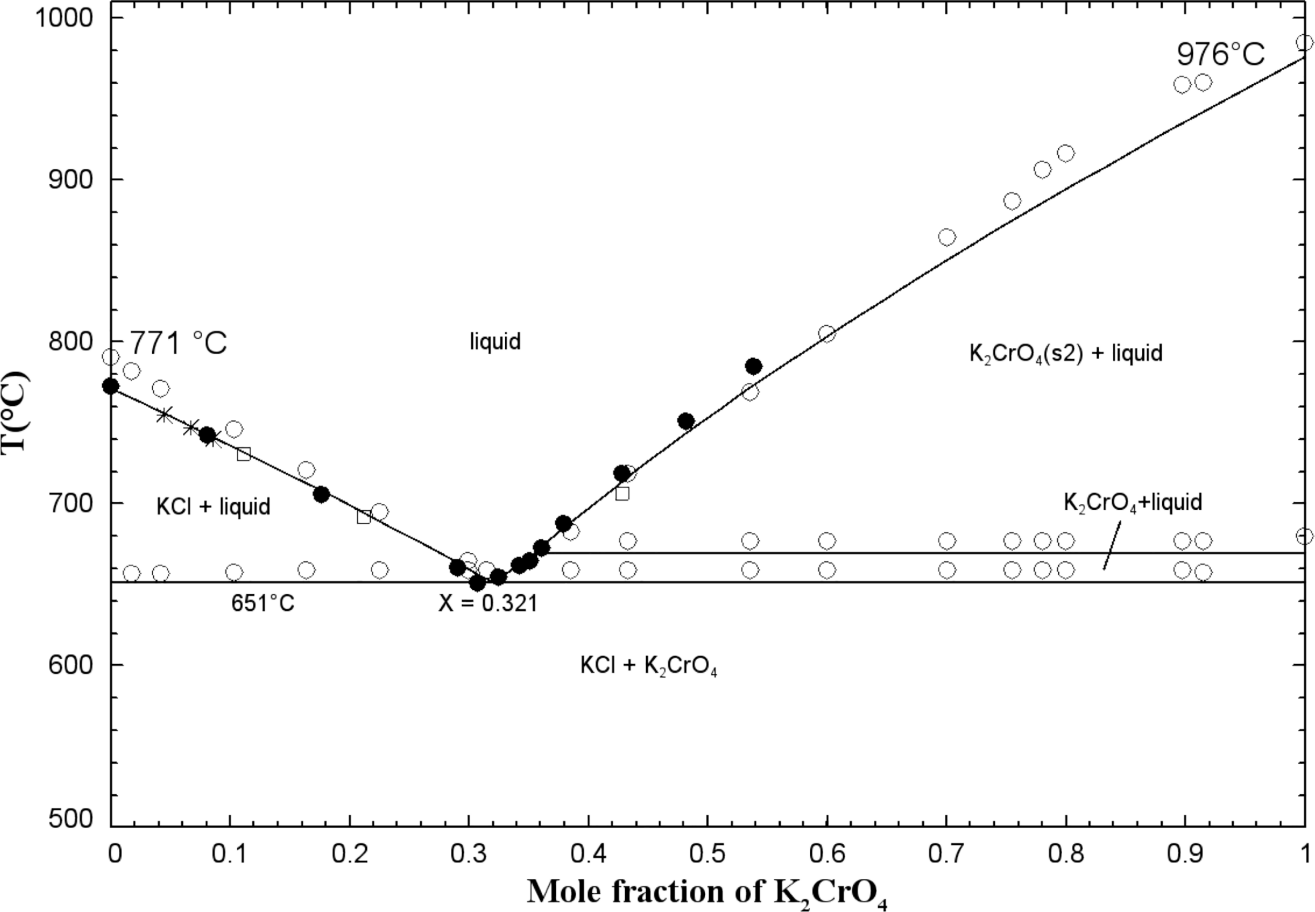
Calculated (KCl + K_2_CrO_4_) phase diagram in air (p­(O_2_) = 0.21 atm). Experimental data from Żemcżużny^34^ (open circle), Sackur^31^ (asterisk), and Rassonskaya and Bergman^35,36^ (filled circle, open square).

#### 5.1.3 The (Na_2_CO_3_ + Na_2_CrO_4_) System

The cooling curve method^37,38^ and DTA^39^ were employed to measure the phase diagram. In this study, DSC-TGA experiments were conducted for binary mixtures containing varying amounts of Na_2_CrO_4_: 5 and 10 mol % (from 250 to 865 °C), as well as 90, 92.5, and 95 mol % (from 250 to 800 °C). Figure 3 shows the DSC thermogram for the binary composition (90 mol % Na_2_CO_3_ + 10 mol % Na_2_CrO_4_) as an illustration.

**Figure 3:**
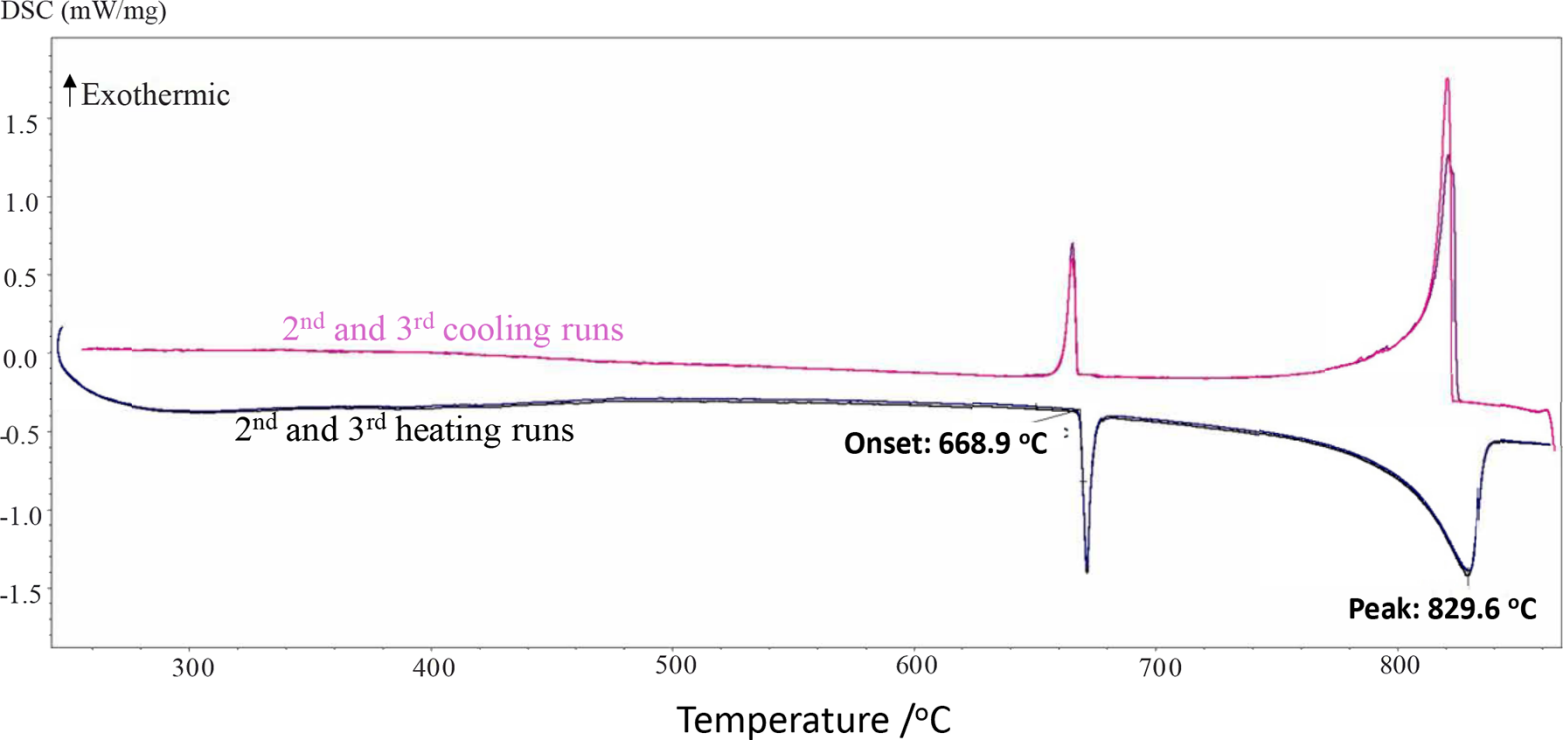
DSC thermogram for the mixture (90 mol % Na_2_CO_3_ + 10 mol % Na_2_CrO_4_) (second and third heating/cooling cycles only).

According to the studies of refs 38,39 , there is a eutectic at 52.0 mol % Na_2_CrO_4_ and 654 °C.

The eutectic is calculated at 53.9 mol % Na_2_CrO_4_ and 650 °C. The high-temperature allotropes Na_2_CO_3(S3)_ and Na_2_CrO_4(S2)_ exhibit isomorphic hexagonal crystal structures and belong to the same space group *P*6_3_/*mmc* (see Table S1 in the Supporting Information). Consequently, the eutectic plateau measured over a wide composition range can be explained by the presence of a miscibility gap. There are two solid solutions, both sharing the same crystal structure (hP22) but with distinct compositions. The measurements of Tathavadkar et al.,^39^ which report a eutectic plateau from 6.0 to 86.8 mol % Na_2_CrO_4_, were favored in the present work. The calculated solid solubility limits are 6.2 and 86.5 mol % Na_2_CrO_4_, respectively. Note that the true solid solubility limits may be somewhat lower than the values suggested by the measurements of Tathavadkar et al.^39^ However, as seen in Figure 4, all available data points are reasonably well reproduced by our model. For the calibration of the liquid phase, there were no experimental data (such as enthalpy of mixing or activity data). The binary liquid displays small positive deviations from ideality (see Table S3 in the Supporting Information). Due to the similar radii of the CO_3_^2–^ (2.10 Å) and CrO_4_^2–^ (2.32 Å) anions,^40^ the (Na_2_CO_3_ + Na_2_CrO_4_) common-cation binary liquid was anticipated to be close to ideal.

**Figure 4:**
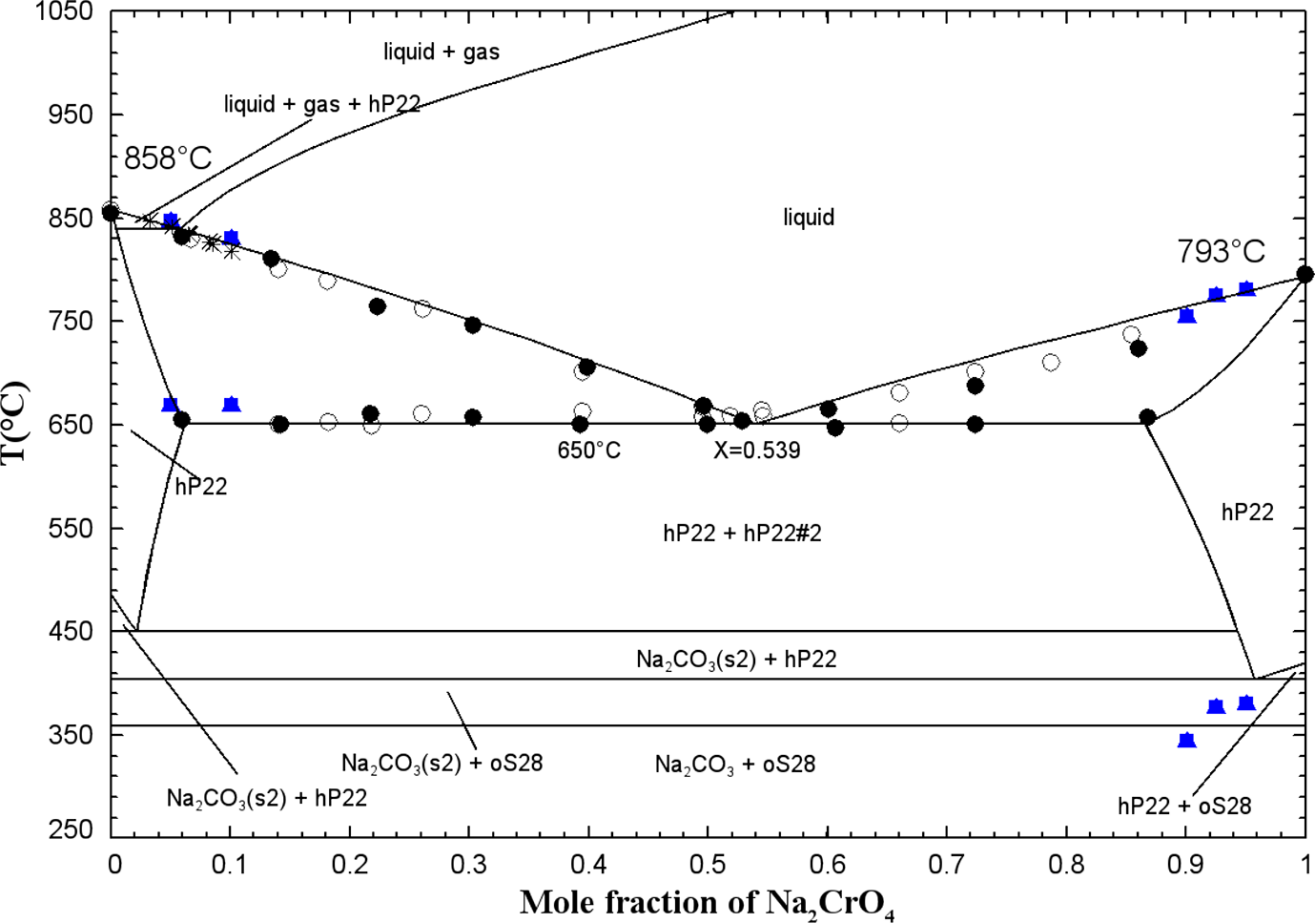
Calculated (Na_2_CO_3_ + Na_2_CrO_4_) phase diagram in air (p­(O_2_) = 0.21 atm). Experimental data from Sackur^37^ (asterisk), Vilnyanski and Pudovkina^38^ (open circle), Tathavadkar et al.^39^ (filled circle), and this work (blue symbols) (DSC, second heating: filled triangle, third heating: filled square).

In our DSC thermograms for Na_2_CrO_4_-rich compositions, a thermal arrest was observed around 360 °C both upon heating and cooling. This is associated with the solid–solid transition Na_2_CO_3(S1)_ = Na_2_CO_3(S2)_, which occurs at 358.9 °C.^16^ Thus, to calculate a negligible solid solubility of Na_2_CO_3_ in the low-temperature allotrope Na_2_CrO_4(S1)_, a regular parameter of +100,000.0 J/mol was introduced in the oS28 solid solution (see Table S4 in the Supporting Information). In our DSC thermograms for Na_2_CO_3_-rich compositions, no signals were detected at low temperatures.

Figure 4 shows the calculated (Na_2_CO_3_ + Na_2_CrO_4_) phase diagram in air (p­(O_2_) = 0.21 atm) along with the available data. The calculated isobar at 1 atm is displayed in this figure and shows that Na_2_CO_3(S3)_ partly decomposes to form liquid Na_2_O (dissolved in the molten salt phase) and CO_2_(g). However, the calculated extent of this decomposition is extremely small (Na_2_O mole fraction of around 10^–9^). As mentioned earlier, in our DSC-TGA measurements, we were able to avoid this decomposition by using a CO_2_(g)-saturated atmosphere.

#### 5.1.4 The (K_2_CO_3_ + K_2_CrO_4_) System

Both the cooling curves method^37,41^ and the visual-polythermal technique^42^ have been used to measure the phase diagram. Sackur^37^ only studied K_2_CO_3_-rich mixtures. Kurnakov and Shemtshushny^41^ reported a continuous series of solid solutions with a minimum at 33.0 mol % K_2_CrO_4_ and 806 °C, while Sanzharov and Bergman^42^ observed an extensive solid solution with a marked minimum at 36.0 mol % K_2_CrO_4_ and 788 °C.

In this study, DSC-TGA experiments were conducted for binary mixtures containing varying amounts of K_2_CrO_4_: 40 and 60 mol % (from 300 to 870 °C), and 80 and 90 mol % (from 300 to 960 °C). Figure 5 displays the DSC thermogram for the binary composition (10 mol % K_2_CO_3_ + 90 mol % K_2_CrO_4_) as an illustration.

**Figure 5:**
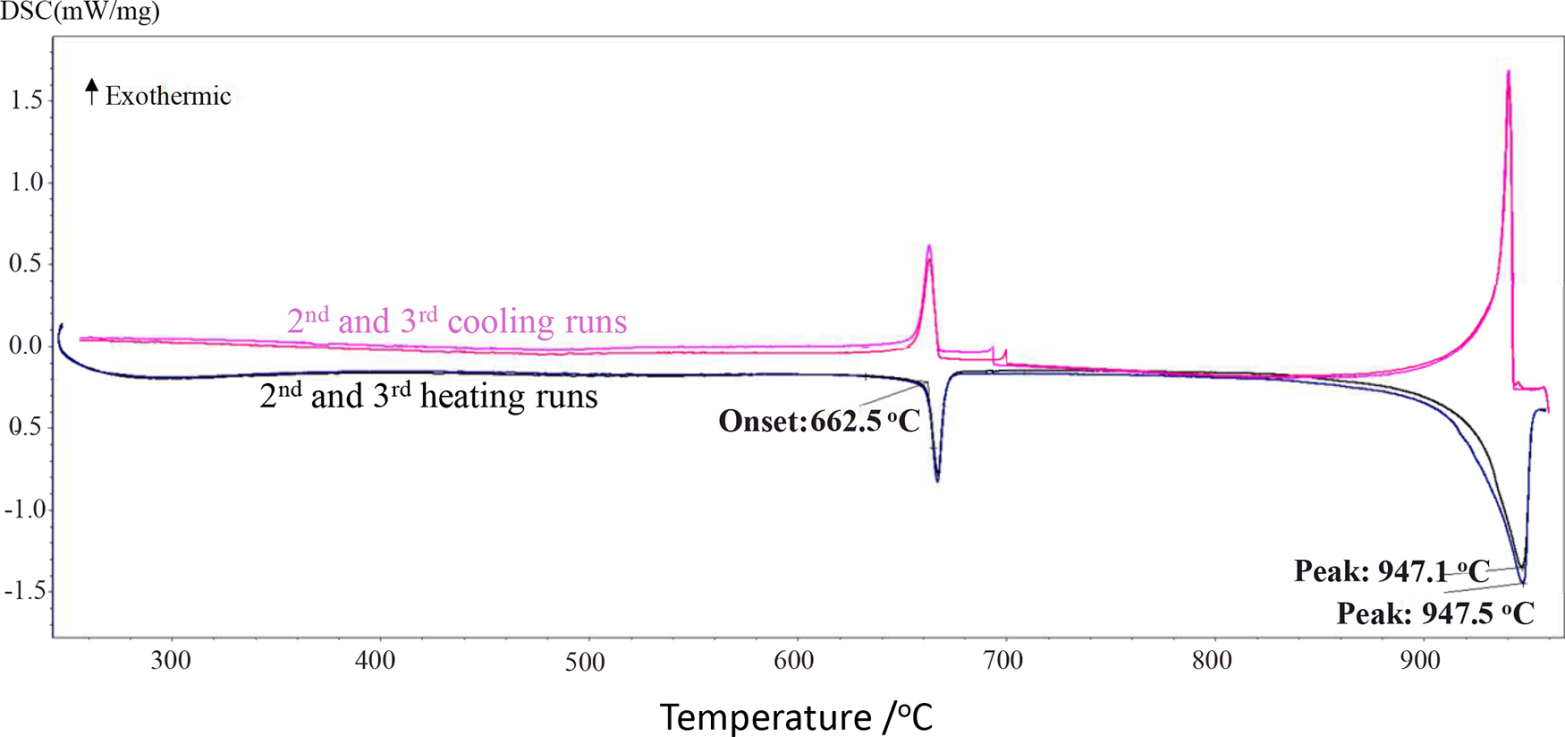
DSC thermogram for the mixture (10 mol % K_2_CO_3_ + 90 mol % K_2_CrO_4_) (second and third heating/cooling cycles only).

Owing to the common hexagonal crystal structure and space group *P*6_3_/*mmc* of the high-temperature allotropes K_2_CO_3(S2)_ and K_2_CrO_4(S2)_ (see Table S1 in the Supporting Information), a solid solution (hP22) was introduced across the entire range of compositions. A minimum was calculated at 39.4 mol % K_2_CrO_4_ and 806 °C. The latter temperature agrees very well with that reported by Kurnakov and Shemtshushny.^41^ Overall, all liquidus and solidus data are reasonably well reproduced by our model (Figure 6). At low temperatures, a miscibility gap (related to the hP22 solid solution) was calculated and agreed well with our low-temperature thermal arrests at 40 and 60 mol % K_2_CrO_4_. As was discussed previously, a miscibility gap (related to hP22) was also observed experimentally in the (Na_2_CO_3_ + Na_2_CrO_4_) phase diagram. Some solid solubility of K_2_CO_3_ in the low-temperature allotrope K_2_CrO_4(S1)_ was introduced (see oP28 solid solution in Table S4 in the Supporting Information) in order to reproduce our low-temperature thermal arrests at 80 and 90 mol % K_2_CrO_4_. For K_2_CO_3_-rich binary mixtures, no experimental data were available at low temperatures, and, therefore, this part of the calculated phase diagram was predicted. For the calibration of the (K_2_CO_3_ + K_2_CrO_4_) liquid phase, there were no experimental data other than phase equilibria (such as enthalpy of mixing or activity data). The binary liquid displays small positive deviations from ideality (Table S3 in the Supporting Information), as was the case for the (Na_2_CO_3_ + Na_2_CrO_4_) liquid. Owing to the similar anionic radii of CO_3_^2–^ and CrO_4_^2–^,^40^ the (K_2_CO_3_ + K_2_CrO_4_) common-cation binary liquid was anticipated to be close to ideal.

**Figure 6:**
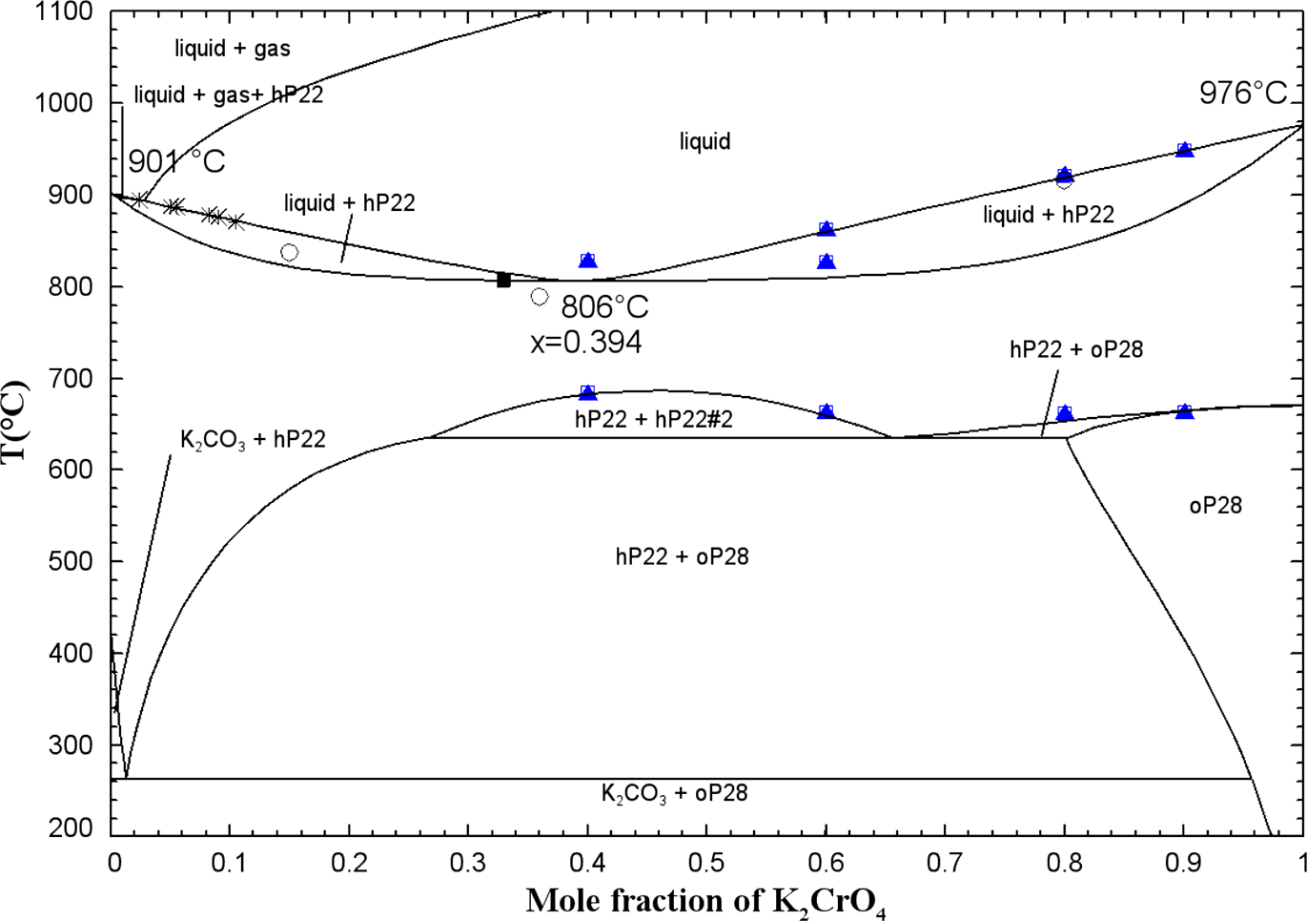
Calculated (K_2_CO_3_ + K_2_CrO_4_) phase diagram in air (p­(O_2_) = 0.21 atm). Experimental data from Kurnakov and Shemtshushny^41^ (filled square), Sackur^37^ (asterisk), Sanzharov and Bergman^42^ (open circle), and this work (blue symbols) (DSC, second heating: open square, third heating: filled triangle).

The calculated (K_2_CO_3_ + K_2_CrO_4_) phase diagram in air (p­(O_2_) = 0.21 atm) is displayed in Figure 6 along with the available data. Based on the calculated isobar at 1 atm included in this figure, K_2_CO_3(S2)_ undergoes partial decomposition, resulting in the formation of liquid K_2_O (which dissolves in the liquid solution) and CO_2_(g). However, the calculated extent of this decomposition is extremely small (K_2_O mole fraction of about 10^–10^). In our DSC-TGA measurements, we were able to avoid this decomposition by using a CO_2_(g)-saturated atmosphere.

#### 5.1.5 The (Na_2_SO_4_ + Na_2_CrO_4_) System

Flach,^43^ Mateiko and Bukhalova,^44^ and Eysel^45^ have measured the phase diagram using the method of cooling curves, the visual-polythermal technique and DTA, respectively. These various data are in agreement (Figure 7) and indicate the existence of a solid solution at high temperatures over the entire composition range and with no minimum,^43–45^ as well as an extensive solid solution at intermediate temperatures extending from 235 °C (for pure Na_2_SO_4_) to 413 °C (for pure Na_2_CrO_4_).^43–45^

**Figure 7:**
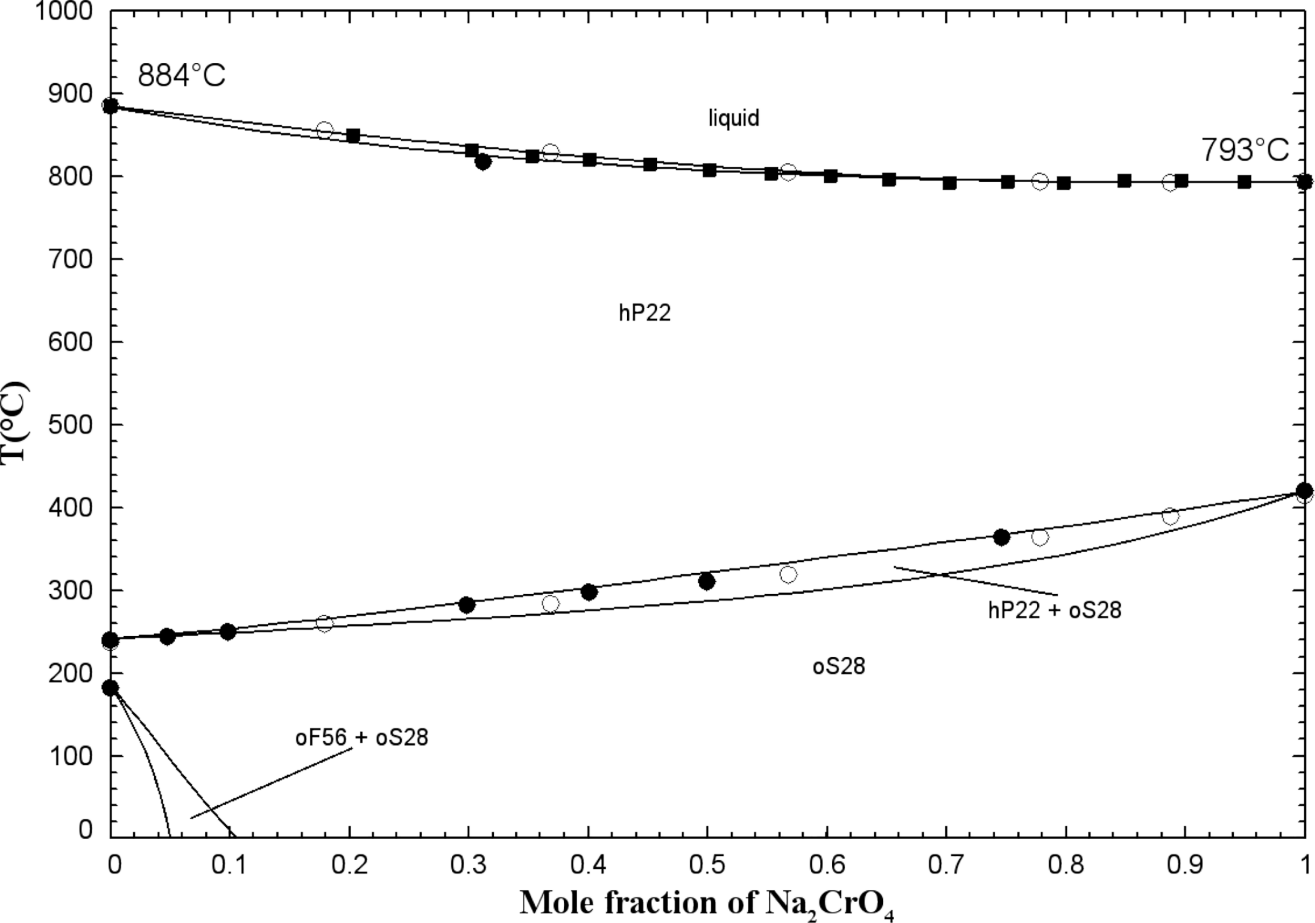
Calculated (Na_2_SO_4_ + Na_2_CrO_4_) phase diagram in air (p­(O_2_) = 0.21 atm). Experimental data from Flach^43^ (open circle), Mateiko and Bukhalova^44^ (filled square), and Eysel^45^ (filled circle).

The crystal structures and space groups of Na_2_SO_4(S2)_ (respectively Na_2_SO_4(S1)_) and Na_2_CrO_4(S2)_ (respectively Na_2_CrO_4(S1)_) are identical, as shown in Table S1 in the Supporting Information. Thus, solid solutions at high temperature (hP22) and low temperature (oS28) were both introduced over the full range of compositions.

At very low temperatures, a Na_2_SO_4(S3)_-rich solid solution (oF56) dissolving Na_2_CrO_4_ was introduced based on the measurements of Eysel.^45^ This author reported the existence of two complete solid solutions: a high-temperature one between Na_2_SO_4(S2)_ and Na_2_CrO_4(S2)_, and a low-temperature one between Na_2_SO_4(S1)_ and Na_2_CrO_4(S1)_. At room temperature, the Na_2_SO_4(S1)_-rich solid solutions were observed to slowly decompose into two different phases: Na_2_SO_4(S3)_-rich solid solutions and Na_2_CrO_4(S1)_-rich solid solutions. The maximum solid solubility in the former was reported to be lower than 5 mol % Na_2_CrO_4_ at 20 °C, based on the evolution of the lattice parameters measured by XRD. The oF56 solid solution was calculated to dissolve 4.73 mol % Na_2_CrO_4_ at 20 °C.

For the calibration of the liquid phase, there were no experimental data (such as enthalpy of mixing or activity data). Due to the close similarity of the radii of the SO_4_^2–^ (2.31 Å) and CrO_4_^2–^ (2.32 Å) anions,^40^ the liquid phase was assumed to be ideal (i.e., Δ*g*_Na_2_/(SO_4_)(CrO_4_)_ = 0).

The calculated (Na_2_SO_4_ + Na_2_CrO_4_) phase diagram in air (p­(O_2_) = 0.21 atm) is compared to the available data in Figure 7.

#### 5.1.6 The (K_2_SO_4_ + K_2_CrO_4_) System

The phase diagram has been investigated using various techniques. Using the method of heating and cooling curves, Groschuff^46^ reported a continuous series of solid solutions with no maximum or minimum at both high and low temperatures. The method of cooling curves has been employed by Amadori.^47^ Eysel^45^ performed DTA measurements, which confirmed a close-to-linear crystallization curve at low temperatures between the two pure compounds, as was previously reported by Groschuff.^46^ Later studies by Bukhalova and Mateiko,^48^ and by Sanzharov and Bergman,^42^ using the visual-polythermal technique, confirmed the high-temperature results of Groschuff^46^ and Amadori.^47^ Sanzharov and Bergman^42^ reported a slight minimum at 80 mol % K_2_CrO_4_ and 968 °C. Finally, visual observations of Le Chatelier^49^ revealed the existence of complete solid solutions at both high and low temperatures, which is in agreement with the other studies. K_2_SO_4(S2)_ (respectively K_2_SO_4(S1)_) and K_2_CrO_4(S2)_ (respectively K_2_CrO_4(S1)_) have the same crystal structure and space group (Table S1 in the Supporting Information). Therefore, a high-temperature solid solution (hP22) and a low-temperature solid solution (oP28) were both introduced over the entire composition range.

For the calibration of the liquid phase, there were no experimental data (such as enthalpy of mixing or activity data). By analogy with the (Na_2_SO_4_ + Na_2_CrO_4_) liquid, an ideal (K_2_SO_4_ + K_2_CrO_4_) liquid phase was assumed (i.e., Δ*g*_K_2_/(SO_4_)(CrO_4_)_ = 0) due to the close similarity of the anionic radii for SO_4_^2–^ and CrO_4_^2–^.^40^

Figure 8 displays the calculated (K_2_SO_4_ + K_2_CrO_4_) phase diagram in air (p­(O_2_) = 0.21 atm) along with the available data.

**Figure 8:**
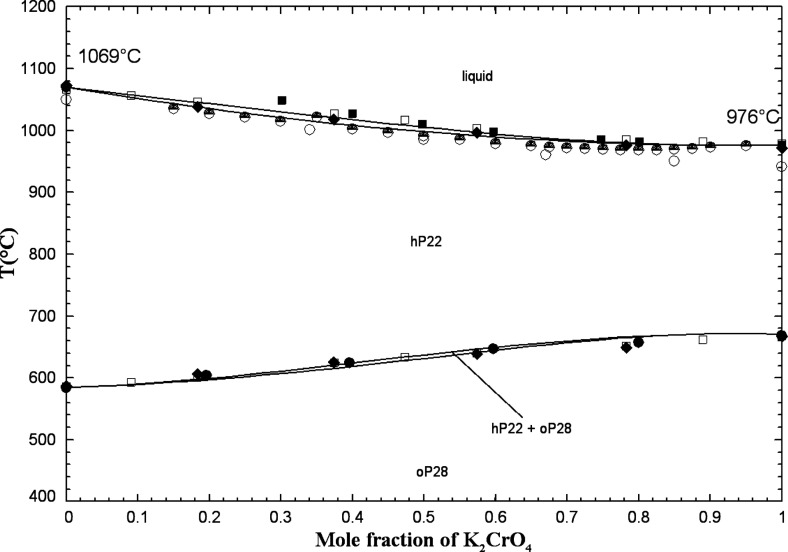
Calculated (K_2_SO_4_ + K_2_CrO_4_) phase diagram in air (p­(O_2_) = 0.21 atm). Experimental data from Eysel^45^ (filled circle), Sanzharov and Bergman^42^ (up-filled circle), Groschuff^46^ (filled diamond), Amadori^47^ (open square), Bukhalova and Mateiko^48^ (filled square), and Le Chatelier^49^ (open circle). Average temperatures of 624.5, 638.5, and 648.0 °C were used in this figure for the temperature ranges of 624–625, 637–640, and 644–652 °C reported by ref 46.

#### 5.1.7 The (Na_2_CrO_4_ + K_2_CrO_4_) System

Several studies have been conducted on the (Na_2_CrO_4_ + K_2_CrO_4_) binary phase diagram. Flach^43^ used the method of cooling curves, while Dergunov and Bergman^50^ and Bergman and Vartbaronov^51^ used the visual-polythermal technique. These investigations revealed the formation at high temperatures of a complete solid solution. The latter exhibits a minimum at approximately 30 mol % K_2_CrO_4_ and 752 °C. The identical hexagonal crystal structures and space groups (*P*6_3_/*mmc*) (see Table S1 in the Supporting Information) of the high-temperature allotropes Na_2_CrO_4(S2)_ and K_2_CrO_4(S2)_ explain the existence of a complete solid solution.

The liquidus and solidus curves measured by Stingele^52^ using high-temperature microscopy are reproducible to within 1 °C for samples of the same composition, and the minimum reported by refs 43,50,51 has been confirmed. The liquidus curve of Amadori^53^ obtained by thermal analysis revealed a minimum at around 25 mol % K_2_CrO_4_ and 740 °C.

Goldberg et al.^54^ performed powder and single-crystal X-ray diffraction, along with DTA and DSC analysis for samples obtained by melting mixtures of Na_2_CrO_4_ and K_2_CrO_4_ and then quenching them in air, or by crystallization from aqueous solutions. Lattice parameters of the quenched samples suggest the formation of two terminal solid solutions based on the low-temperature allotropes Na_2_CrO_4(S1)_ and K_2_CrO_4(S1)_. This is consistent with the fact that these are orthorhombic with different space groups (Table S1 in the Supporting Information). At intermediate compositions, a trigonal chrome-glaserite phase forming an extended solid solution around the composition NaK_3_(CrO_4_)_2_ and stable at room temperature is reported.^45,54^ Eysel’s results^45^ suggest that chrome-glaserite should be considered as a solid solution and that the glaserite phases in the (Na_2_CrO_4_ + K_2_CrO_4_) and (Na_2_SO_4_ + K_2_SO_4_) systems have close similarities. The chrome-glaserite solid solution exhibits a temperature maximum around the composition (Na_0.45_K_0.55_)_2_CrO_4_ and not around NaKCrO_4_.^45^

Flach^43^ considered the chrome-glaserite phase to be a stoichiometric compound NaK_3_(CrO_4_)_2_, and this author reported no solid solubility in the low-temperature allotropes Na_2_CrO_4(S1)_ and K_2_CrO_4(S1)_. He observed a maximum temperature at around 50–55 mol % K_2_CrO_4_ and 607 °C. Amadori^53^ reported a flat maximum between 45 and 60 mol % K_2_CrO_4_ and at 606 °C, which was attributed to the formation of the compound Na_2_CrO_4_·K_2_CrO_4_ from the high-temperature hexagonal solid solution (hP22).

Glaserites are solid solutions of the type (A,C)_2_BX_4_, where the A cations include Na^+^, Ag^+^, Mg^2+^, Ca^2+^, and Cd^2+^; the C cations include K^+^, Rb^+^, Cs^+^, NH_4_^+^, Sr^2+^, Ba^2+^, and Eu^2+^; and the tetrahedral complex anions BX_4_ include BeF_4_^2–^, SO_4_^2–^, SeO_4_^2–^, CrO_4_^2–^, MoO_4_^2–^, WO_4_^2–^, PO_4_^3–^, VO_4_^3–^, AsO_4_^3–^, SiO_4_^4–^, and GeO_4_^4–^.^45^

The crystal structure and space group of the two reported forms of chrome-glaserite K_3_Na­(CrO_4_)_2_ are given in Table 3.

**Table 3:** Crystal Structure and Space Group of Chrome-Glaserite K_3_Na­(CrO_4_)_2_

**K**_**3**_ **Na(CrO**_**4**_ **)**_**2**_	**crystal structure**	**space group**	**Pearson symbol/phase prototype**	**reference**
low-temperature form	monoclinic (stable below −34.15 °C)	*C*2/*c*	—	^54–57^
high-temperature form	trigonal (stable above −34.15 °C)	*P*3̅*m*1 (164)	hP14/K_3_Na(SO_4_)_2_	^54–57^

Figure 9 displays the calculated (Na_2_CrO_4_ + K_2_CrO_4_) phase diagram in air (p­(O_2_) = 0.21 atm) along with the available data. There are four distinct solid solutions: a high-temperature hexagonal solid solution (hP22) that exists across the entire composition range, two orthorhombic low-temperature solid solutions (oS28, which is rich in Na_2_CrO_4(S1)_ and diluted in K_2_CrO_4_, and oP28, which is rich in K_2_CrO_4(S1)_ and diluted in Na_2_CrO_4_), and the nonstoichiometric phase K_3_Na­(CrO_4_)_2_ (chrome-glaserite, hP14). The low-temperature form of chrome-glaserite was not considered in the present work since it is stable at very low temperatures. Table S4 in the Supporting Information provides detailed descriptions of these various solid solutions, including the optimized model parameters.

**Figure 9:**
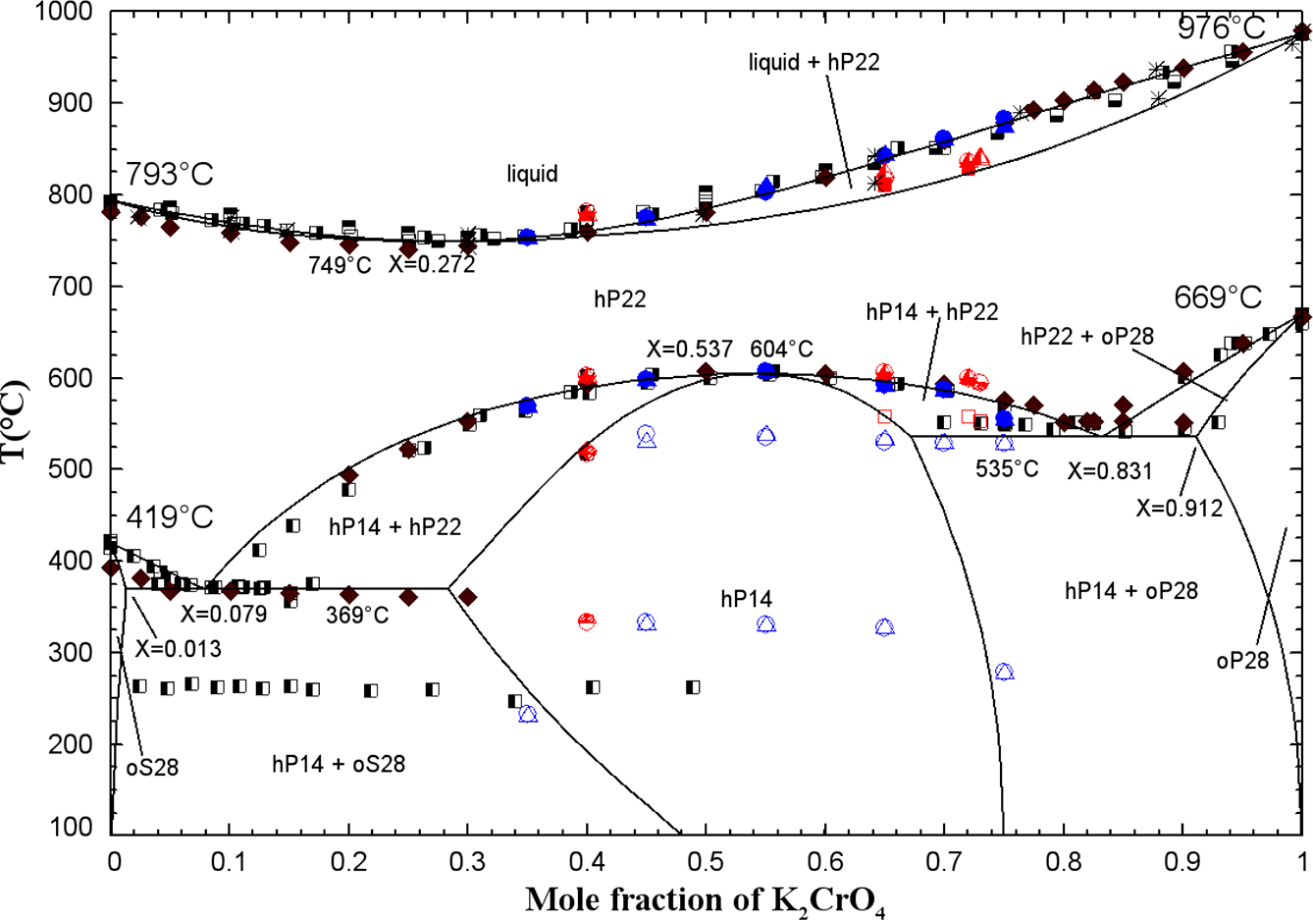
Calculated (Na_2_CrO_4_ + K_2_CrO_4_) phase diagram in air (p­(O_2_) = 0.21 atm). Experimental data from Flach^43^ (right-filled square), Amadori^53^ (filled diamond), Dergunov and Bergman^50^ (up-filled square), Bergman and Vartbaronov^51^ (down-filled square), Stingele^52^ (asterisk), Goldberg et al.^54^ (left-filled square), and this work (blue and red symbols) (DSC, red filled square: first heating for chrome-glaserite equilibrated at 400 °C for 3 weeks, red open square: very low-intensity peak of first heating for chrome-glaserite equilibrated at 400 °C for 3 weeks, blue filled circle: second heating for mechanical mixtures of pretreated reagents, blue open circle: very low-intensity peak of second heating for mechanical mixtures of pretreated reagents, red down-filled circle: second heating for chrome-glaserite equilibrated at 400 °C for 3 weeks, red up-filled circle: very low-intensity peak of second heating for chrome-glaserite equilibrated at 400 °C for 3 weeks, blue filled triangle: third heating for mechanical mixtures of pretreated reagents, blue open triangle: very low-intensity peak of third heating for mechanical mixtures of pretreated reagents, red left-filled triangle: third heating for chrome-glaserite equilibrated at 400 °C for 3 weeks, red left-filled diamond: very low-intensity peak of third heating for chrome-glaserite equilibrated at 400 °C for 3 weeks).

As indicated in this table, the glaserite phase was modeled with the sublattice structure: (K^+^, Na^+^)_3_(Na^+^)­(SO_4_^2–^, CrO_4_^2–^)_2_. The cations Na^+^ and K^+^ (positions 1a and 2a) occupy the first sublattice, while the Na^+^ cation (position 1b) occupies the second sublattice. Finally, the anions SO_4_^2–^ and CrO_4_^2–^ occupy the third sublattice. Previously, the sulfur-glaserite phase was modeled,^15^ and the associated model parameters were directly used in this study. Only a trigonal form (space group *P*3̅*m*1) has been reported.^45,58^ For the modeling of the chrome-glaserite phase, two new “end-members” (K_3_Na­(CrO_4_)_2_ and Na_3_Na­(CrO_4_)_2_) were added to the existing hP14 solid solution. The only model parameters used were their Gibbs energies; similarly to the sulfur-glaserite phase, no interaction parameters were required.^15^

The present investigation involved DSC-TGA experiments for a series of binary mixtures of the pretreated reagents Na_2_CrO_4_·4H_2_O and K_2_CrO_4_. A total of six mixtures were studied, each containing varying amounts of K_2_CrO_4_: 35, 45, 55, and 65 mol % (analyzed from 30 to 860 °C), as well as 70 and 75 mol % (analyzed from 30 to 890 °C).

The chrome-glaserite composition of (45 mol % Na_2_CrO_4_ + 55 mol % K_2_CrO_4_) was also studied using DSC-TGA. This composition had been equilibrated at 400 °C for 3 weeks, and an excess of Na_2_CrO_4_ or K_2_CrO_4_ had been added subsequently. Four binary mixtures with different amounts of K_2_CrO_4_ were analyzed under those conditions: 40 and 73 mol % (from 30 to 890 °C) and 65 and 72 mol % (from 30 to 920 °C).

Tables S7 and S8 in the Supporting Information provide a comprehensive list of all measured thermal arrests. As an illustration, Figure 10 displays the DSC thermogram for the equilibrated chrome-glaserite sample with the composition of (35 mol % Na_2_CrO_4_ + 65 mol % K_2_CrO_4_) and with the subsequent addition of an excess of pretreated K_2_CrO_4_.

**Figure 10:**
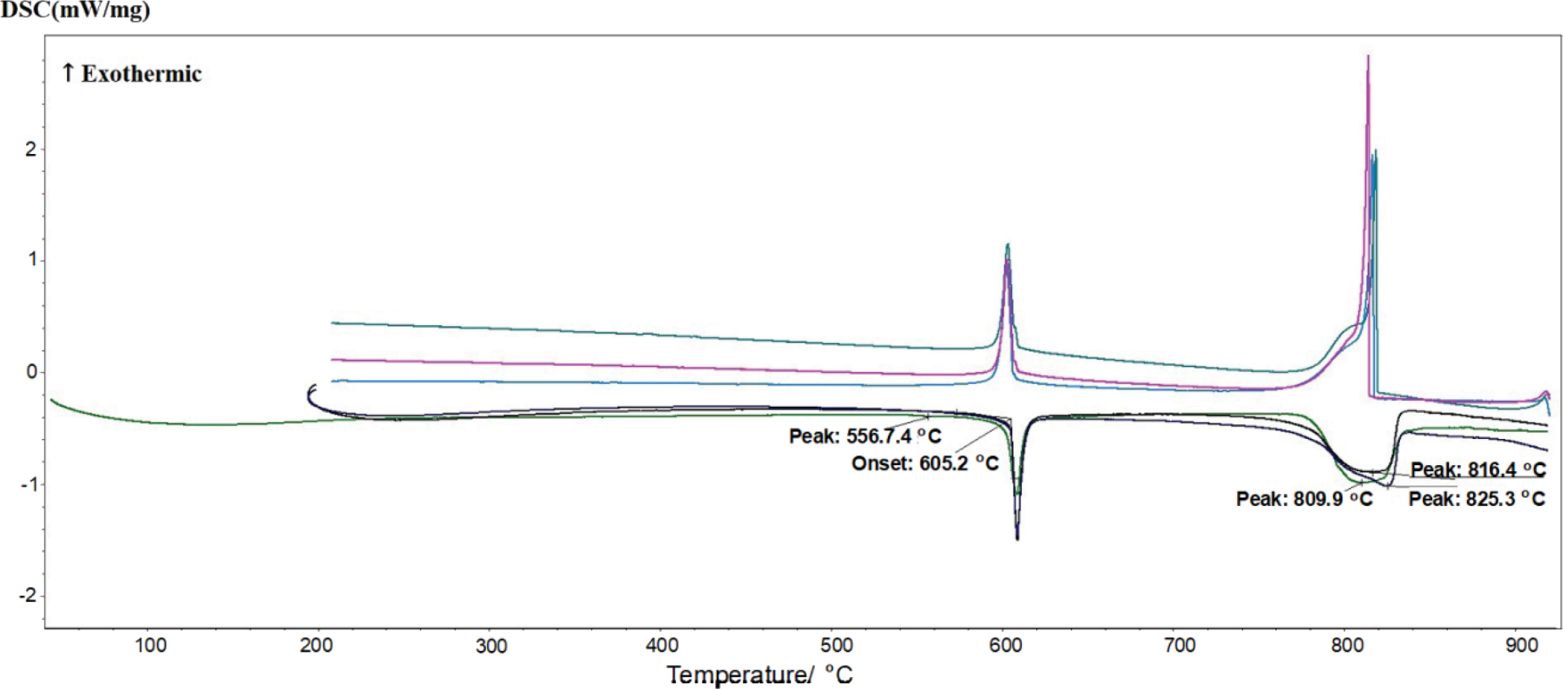
DSC thermogram for the composition (35 mol % Na_2_CrO_4_ + 65 mol % K_2_CrO_4_) of the equilibrated chrome-glaserite sample to which an excess of pretreated K_2_CrO_4_ was added subsequently (first, second, and third heating/cooling cycles). The temperature value of 556.7 °C for the first heating run was taken at the maximum of the peak (and not at the onset of the peak) since this was a very low-intensity peak.

For the calibration of the (Na_2_CrO_4_ + K_2_CrO_4_) liquid phase, there were no experimental data (such as enthalpy of mixing or activity data). Thus, as for the (Na_2_SO_4_ + K_2_SO_4_) liquid phase modeled by Lindberg et al.,^15^ a constant parameter of −1419.6 J/mol was selected in this work. This parameter had been used to reproduce the enthalpy of mixing data. Note that the SO_4_^2–^ and CrO_4_^2–^ anions have very similar radii.^40^

Thereafter, the hexagonal solid solution (hP22) was modeled to best reproduce the high-temperature measurements, especially the minimum of hP22 at around 30 mol % K_2_CrO_4_ ^43,50–52^ and the maximum extension of the chrome-glaserite phase (hP14) at approximately 605 °C.^54^ For hP22, three model parameters were required. For hP14, simultaneous optimization of the Gibbs energies of the two “end-members” K_3_Na­(CrO_4_)_2_ and Na_3_Na­(CrO_4_)_2_ was performed. Finally, the two low-temperature terminal solid solutions (oS28 and oP28) were modeled in order to best reproduce the experimental temperatures and compositions of the two eutectoid reactions as well as the measured solubility limits. Temperatures of 369 and 535 °C are calculated for the eutectoid reactions hP22 = oS28 + hP14 and hP22 = hP14 + oP28, respectively. These compare reasonably well with the values reported by ref 54 (374 and 550 °C, respectively) and by ref 53 (between 360 and 366 °C, and between 550 and 552 °C, respectively). For the eutectoid temperature calculated at 369 °C, both the data of Goldberg et al.^54^ and Amadori^53^ were considered. For the eutectoid temperature calculated at 535 °C, our DSC-TGA measurements for equilibrated chrome-glaserite (to which an excess of K_2_CrO_4_ was added subsequently) as well as the data of Goldberg et al.^54^ and Amadori^53^ were taken into account.

The solubility limits for the two terminal solid solutions were estimated by Goldberg et al.^54^ using X-ray diffraction measurements of the lattice parameters. Our calculated maximum solubility of 1.3 mol % K_2_CrO_4_ (at 369 °C) in oS28 is somewhat lower than the value of 4.0 mol % K_2_CrO_4_ reported by ref 54. Our calculated maximum solubility of 8.8 mol % Na_2_CrO_4_ (at 535 °C) in oP28 is close to the reported value of 7 mol % Na_2_CrO_4_ from Goldberg et al.^54^

Goldberg et al.’s DTA measurements^54^ for Na_2_CrO_4_-rich samples showed a very weak reversible peak at about 266 °C, which was not observed for pure Na_2_CrO_4_. This peak had its maximum intensity at 25 mol % K_2_CrO_4_ and then progressively disappeared around 50 mol % K_2_CrO_4_.^54^ Goldberg et al. did not provide any interpretation for this thermal arrest. We concluded that it might be attributed to the formation of Na_2_Cr_2_O_7_ or K_2_Cr_2_O_7_ in the presence of small amounts of CrO_3_. Indeed, the reactions Na_2_CrO_4_ + CrO_3_ = Na_2_Cr_2_O_7_ and K_2_CrO_4_ + CrO_3_ = K_2_Cr_2_O_7_ have very negative Gibbs energy changes at least up to the melting temperatures of the pure chromates. Also, the Na_2_Cr_2_O_7(S1)_ = Na_2_Cr_2_O_7(S2)_ and K_2_Cr_2_O_7(S1)_ = K_2_Cr_2_O_7(S2)_ solid–solid transitions occur at 249.6 and 267.2 °C, respectively, which are relatively close to the value of 266 °C.^19^

Figures 11 and 12 show the calculated (Na_2_CrO_4_ + K_2_CrO_4_) phase diagrams in the presence of 0.1 mol % CrO_3_ along with the available data. Figure 11 includes as possible products the pure dichromates along with all dichromate-based solid solutions modeled in the present work (aP44, aP22, and K_2_Cr_2_O_7_(s.s)). The latter are described in Table S4 in the Supporting Information and will be discussed later. Figure 12 includes as possible products the pure dichromates, while the formation of all dichromate-based solid solutions is inhibited. It can be seen that the K_2_Cr_2_O_7(S1)_ = K_2_Cr_2_O_7(S2)_ solid–solid transition agrees closely with the thermal arrest observed around 266 °C by Goldberg et al.^54^ Thus, the latter might be due to trace amounts of CrO_3_ in the Na_2_CrO_4_ and K_2_CrO_4_ reagents used by these authors. However, it is unclear why the formation of the dichromate-based solid solutions would be impeded, thus favoring the formation of pure dichromates.

**Figure 11:**
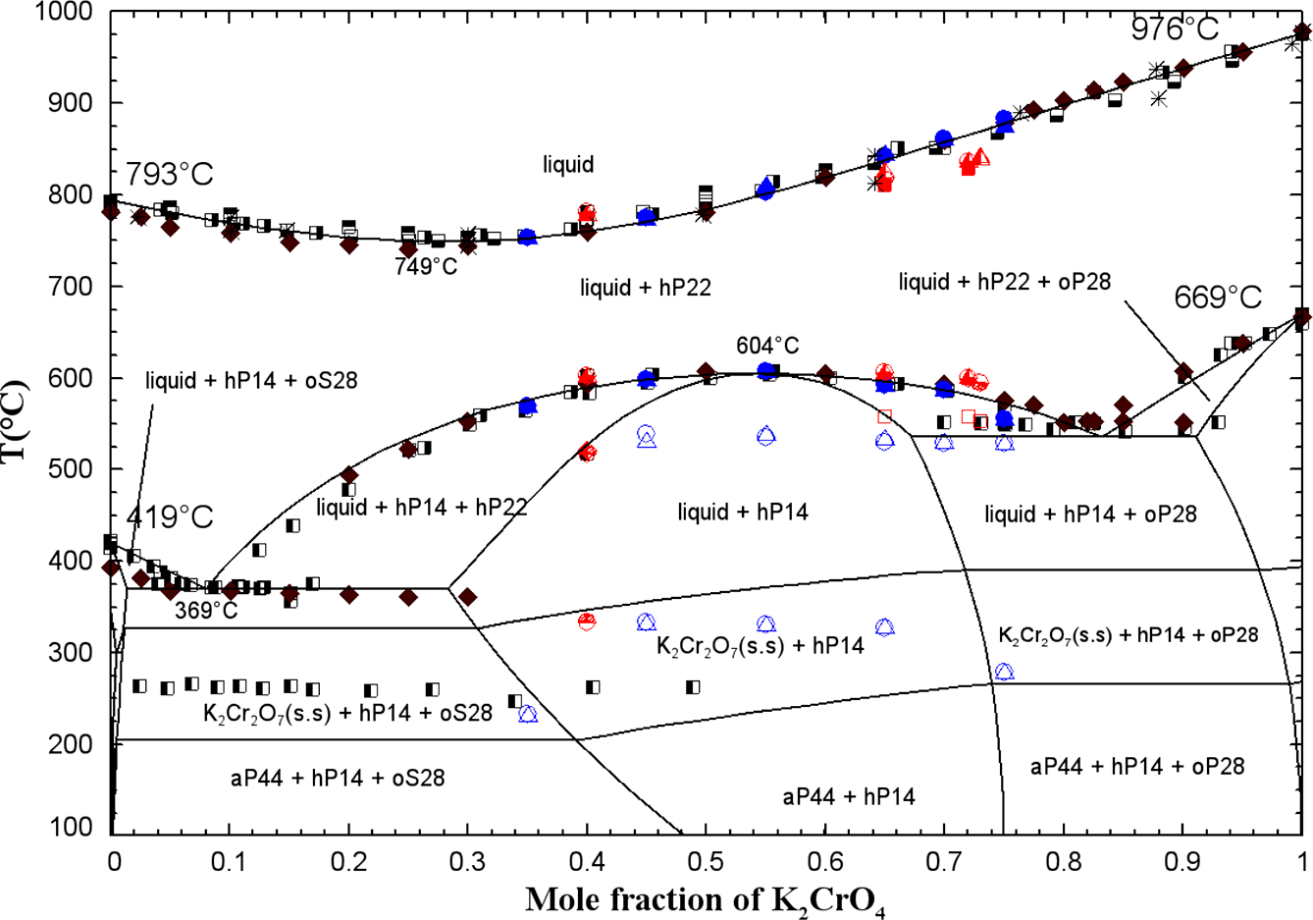
Calculated (Na_2_CrO_4_ + K_2_CrO_4_) phase diagram in the presence of 0.1 mol % CrO_3_, with possible formation of the pure dichromates and all dichromate-based solid solutions (aP44, aP22, and K_2_Cr_2_O_7_(s.s)). The symbols used are defined in Figure 9.

**Figure 12:**
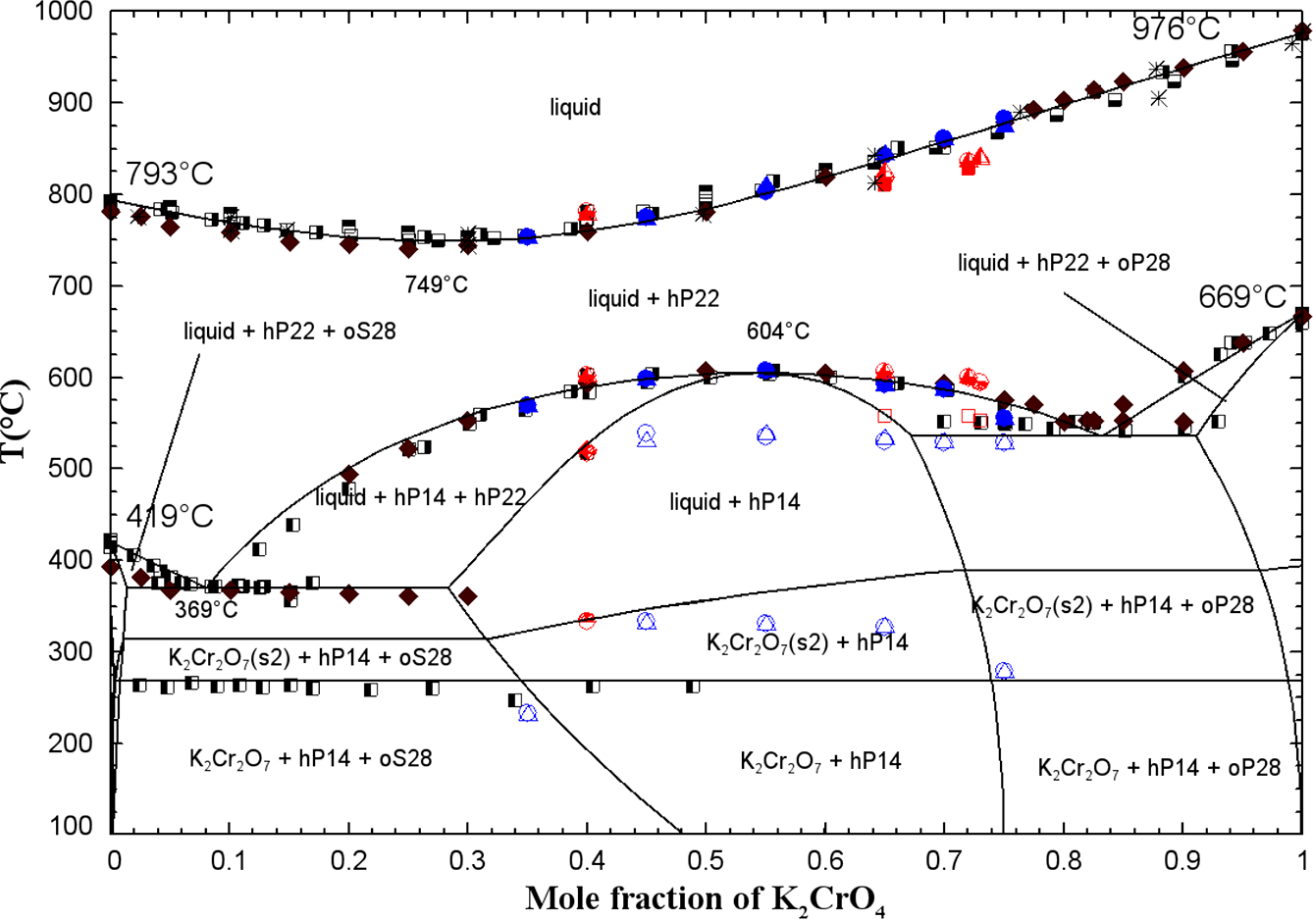
Calculated (Na_2_CrO_4_ + K_2_CrO_4_) phase diagram in the presence of 0.1 mol % CrO_3_, with possible formation of the pure dichromates and with inhibited formation of all dichromate-based solid solutions (aP44, aP22, and K_2_Cr_2_O_7_(s.s)). The symbols used are defined in Figure 9.

#### 5.1.8 The (KCl + K_2_Cr_2_O_7_) System

Phase diagram measurements were conducted using the cooling curves method.^34^ The binary system (KCl + K_2_Cr_2_O_7_) is of simple eutectic type; no solid solutions or intermediate compounds have been evidenced. It was not possible to reproduce the data of Żemcżużny^34^ near pure KCl since its melting point reported by Żemcżużny is about 20 °C higher than the accepted value of 771 °C selected in the present work.^22^ Żemcżużny^34^ reported a eutectic at 72.5 mol % K_2_Cr_2_O_7_ and 366 °C. The eutectic is calculated at 73.2 mol % K_2_Cr_2_O_7_ and 364 °C.

The binary liquid exhibits small negative deviations from ideality (Table S3 in the Supporting Information). Figure 13 displays the calculated (KCl + K_2_Cr_2_O_7_) phase diagram in air (p­(O_2_) = 0.21 atm) along with the available data.

**Figure 13:**
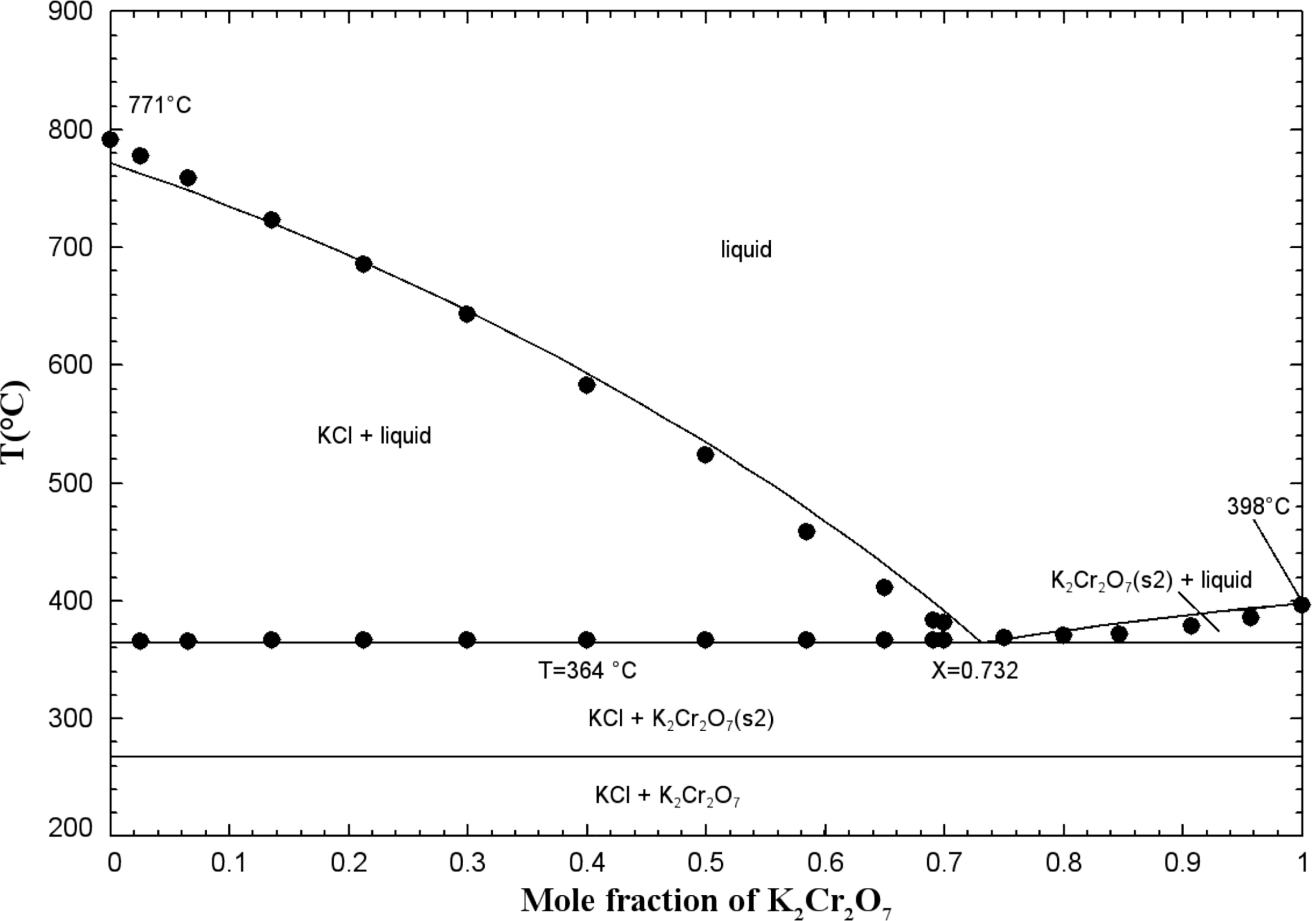
Calculated (KCl + K_2_Cr_2_O_7_) phase diagram in air (p­(O_2_) = 0.21 atm). Experimental data from Żemcżużny^34^ (filled circle).

#### 5.1.9 The (K_2_CrO_4_ + K_2_Cr_2_O_7_) System

The method of heating and cooling curves has been employed to measure the phase diagram.^46^ According to Groschuff, a eutectic was observed at 98.5 mol % K_2_Cr_2_O_7_ and 393 °C. The latter temperature is very close to the melting point of pure potassium dichromate. The eutectic is calculated at 95.9 mol % K_2_Cr_2_O_7_ and 393 °C. The binary liquid was assumed to exhibit ideal behavior (i.e., Δ*g*_K_2_/(CrO_4_)(Cr_2_O_7_)_ = 0). Figure 14 displays the calculated (K_2_CrO_4_ + K_2_Cr_2_O_7_) phase diagram in air (p­(O_2_) = 0.21 atm) as well as the available data.

**Figure 14:**
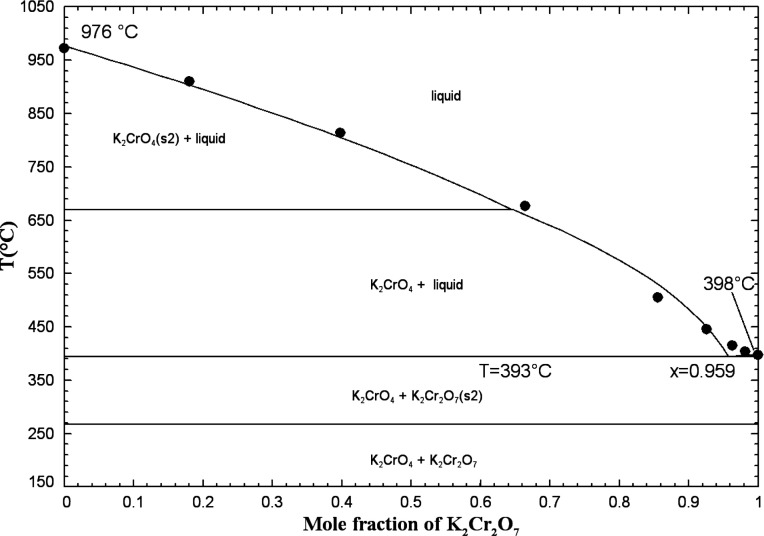
Calculated (K_2_CrO_4_ + K_2_Cr_2_O_7_) phase diagram in air (p­(O_2_) = 0.21 atm). Experimental data from Groschuff^46^ (filled circle).

Flood and Muan^59^ measured the K_2_CrO_4_ content of (K_2_Cr_2_O_7_ + K_2_CrO_4_) melts from 610 to 760 °C, using a gravimetric method. These authors started with a weighted sample of K_2_Cr_2_O_7_ saturated with Cr_2_O_3_ at a fixed partial pressure of O_2_(g) of 1 or 0.21 atm (air). Under those conditions, the following equilibrium was reached:

$$K_{2}{\mathrm{Cr}}_{2}O_{7\left(\mathrm{liquid}\right)}=K_{2}{\mathrm{CrO}}_{4\left(\mathrm{liquid}\right)}+0.5{\mathrm{Cr}}_{2}O_{3\left(s\right)}+0.75O_{2\left(g\right)}$$ 2

The amount of O_2_(g) expelled during adjustment to equilibrium was determined by weighing, after rapid cooling.^59^ Starting from the K_2_CrO_4_ side of reaction 2 was unsuccessful; O_2_(g) was consumed by the melt, but this process was extremely slow. By starting from the K_2_Cr_2_O_7_ side of reaction 2, equilibrium was attained in 2–6 h at p­(O_2_) = 1 atm, but more time was required in air.^59^ The calculated K_2_Cr_2_O_7_ content (as a function of temperature) of (K_2_Cr_2_O_7_ + K_2_CrO_4_) melts saturated with Cr_2_O_3_ and at a fixed partial pressure of O_2_(g) of 1 or 0.21 atm is shown along with the measurements of Flood and Muan in Figure 15. As a starting point, the thermodynamic data for solid Cr_2_O_3_ were taken directly from the FToxid database in FactSage.^21^ The black lines in Figure 15 represent calculations for (K_2_Cr_2_O_7_ + K_2_CrO_4_) melts, assuming an ideal liquid. These calculations underestimate the data of Flood and Muan.^59^ The calculated K_2_Cr_2_O_7_ content of the melt depends on the Gibbs energy change for reaction 2, which depends itself on the Gibbs energies of K_2_Cr_2_O_7(liquid)_, K_2_CrO_4(liquid)_, Cr_2_O_3(s)_, and O_2(g)_. The Gibbs energies of liquid K_2_CrO_4_ ^18^ and K_2_Cr_2_O_7_ ^19^ were both evaluated by us from the literature and are expected to be accurate. The thermodynamic data for O_2_(g) are well known; they were taken directly from the FactPS database in FactSage.^21^ The thermodynamic data for Cr_2_O_3(s)_ may be less accurate: for instance, its Δ*H*_298.15K_^°^ values are −1,127,120 and −1,140,600 J/mol, respectively, in the FToxid and SGPS databases in FactSage.^21^ There is a shift of about 13 kJ/mol between these two values. In Figure 15, the blue lines correspond to a (K_2_Cr_2_O_7_ + K_2_CrO_4_) ideal liquid, but the Δ*H*_298.15K_^°^ value for solid Cr_2_O_3_ is 4 kJ/mol higher than that for the black lines. Agreement is excellent between these new calculations and the measurements of Flood and Muan.^59^

**Figure 15:**
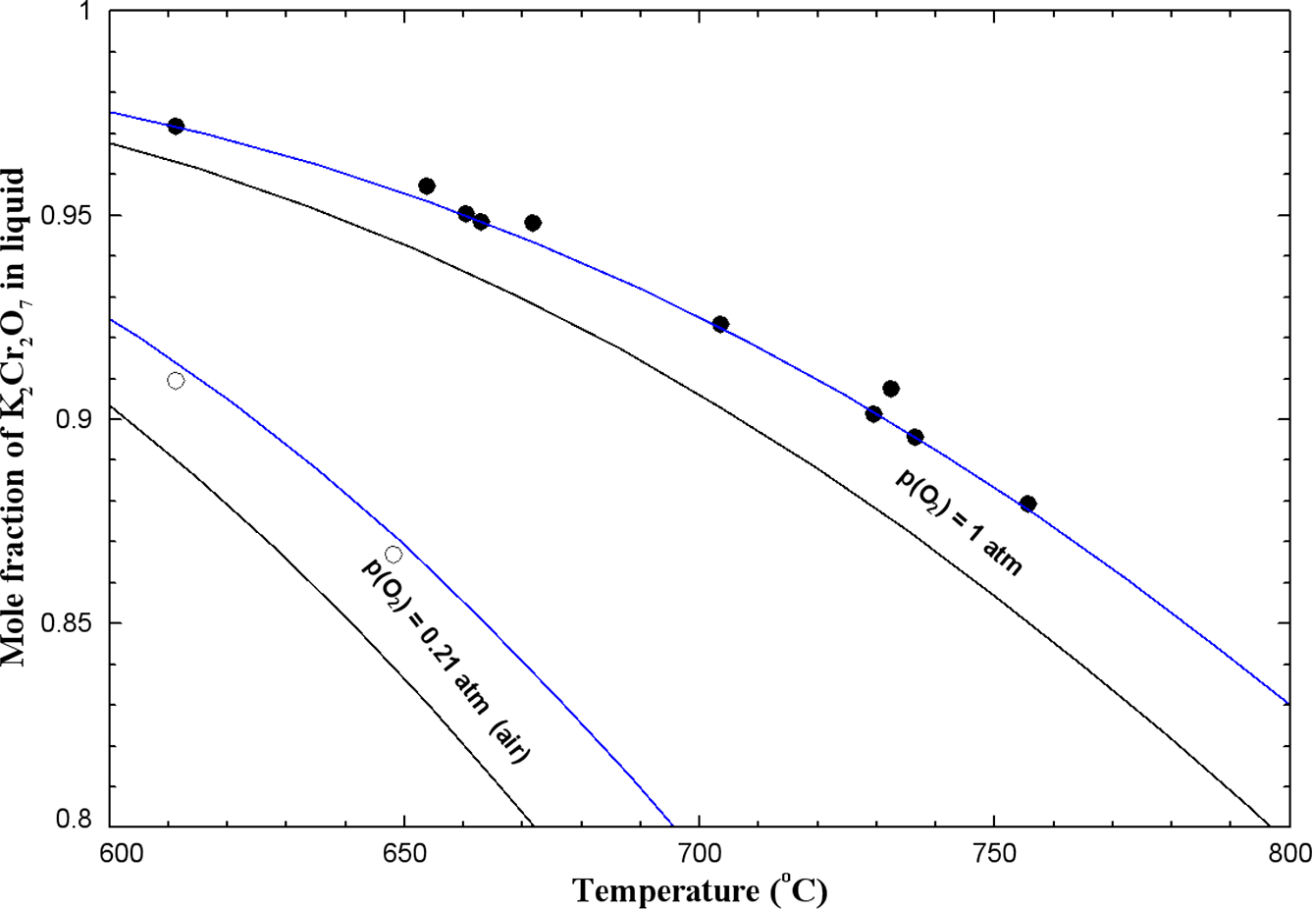
Calculated K_2_Cr_2_O_7_ content of (K_2_Cr_2_O_7_ + K_2_CrO_4_) melts saturated with Cr_2_O_3_ and at a fixed partial pressure of O_2_(g) of 1 or 0.21 atm (air). Black lines: (K_2_Cr_2_O_7_ + K_2_CrO_4_) ideal liquid, and Δ*H*_298.15K_^°^(Cr_2_O_3_(s)) taken from the FToxid database; blue lines: (K_2_Cr_2_O_7_ + K_2_CrO_4_) ideal liquid, and Δ*H*_298.15K_^°^(Cr_2_O_3_(s)) is 4 kJ/mol higher than that from the FToxid database. Experimental data from Flood and Muan^59^ (filled circle: p­(O_2_) = 1 atm, open circle: in air - p­(O_2_) = 0.21 atm).

#### 5.1.10 The (Na_2_CrO_4_ + Na_2_Cr_2_O_7_) System

To our knowledge, this phase diagram has not been measured. Flood and Muan^59^ measured the Na_2_CrO_4_ content of (Na_2_Cr_2_O_7_ + Na_2_CrO_4_) melts from 400 to 550 °C, using the same experimental protocol as described previously for the (K_2_Cr_2_O_7_ + K_2_CrO_4_) system. The calculated Na_2_Cr_2_O_7_ content (as a function of temperature) of (Na_2_Cr_2_O_7_ + Na_2_CrO_4_) melts saturated with Cr_2_O_3_ and at a fixed partial pressure of O_2_(g) of 1 atm is compared to the measurements of Flood and Muan^59^ in Figure 16. Different scenarios were considered. Scenario 1 corresponds to a (Na_2_Cr_2_O_7_ + Na_2_CrO_4_) ideal liquid: The calculated Na_2_Cr_2_O_7_ content is about 84.4 mol % at 405 °C, which is significantly lower than the measured value of about 91.1 mol %.^59^ Also, it decreases much more rapidly with temperature than the data of Flood and Muan, and the liquid phase is calculated to disappear completely at about 469 °C with the formation of Na_2_CrO_4(S2)_. This clearly disagrees with the results of those authors. The calculated Na_2_Cr_2_O_7_ content of the melt depends on the Gibbs energy change for reaction 2 (in which K would be replaced with Na), which depends itself on the Gibbs energies of Na_2_Cr_2_O_7(liquid)_, Na_2_CrO_4(liquid)_, Cr_2_O_3(s)_, and O_2(g)_. The Gibbs energy of liquid Na_2_CrO_4_ was evaluated by us from the literature^18^ and is expected to be accurate. As reported previously, the thermodynamic data for O_2_(g) are well known. On the other hand, owing to the lack of data, some estimations were sometimes made for Na_2_Cr_2_O_7_, and the selected thermodynamic data were therefore less accurate.^19^ As already discussed previously, the thermodynamic data for Cr_2_O_3(s)_ may be less accurate too. In Figure 16, scenario 2 corresponds to a (Na_2_Cr_2_O_7_ + Na_2_CrO_4_) ideal liquid, but the Δ*H*_298.15K_^°^ value for solid Cr_2_O_3_ is 4 kJ/mol higher than that for scenario 1 (i.e., the same value used for our calculations related to the (K_2_Cr_2_O_7_ + K_2_CrO_4_) system: see the blue curves in Figure 15). The calculated Na_2_Cr_2_O_7_ content of about 88.6 mol % at 405 °C is now relatively close to the experimental value, and the liquid phase is calculated to be stable over the entire temperature range investigated by Flood and Muan.

**Figure 16:**
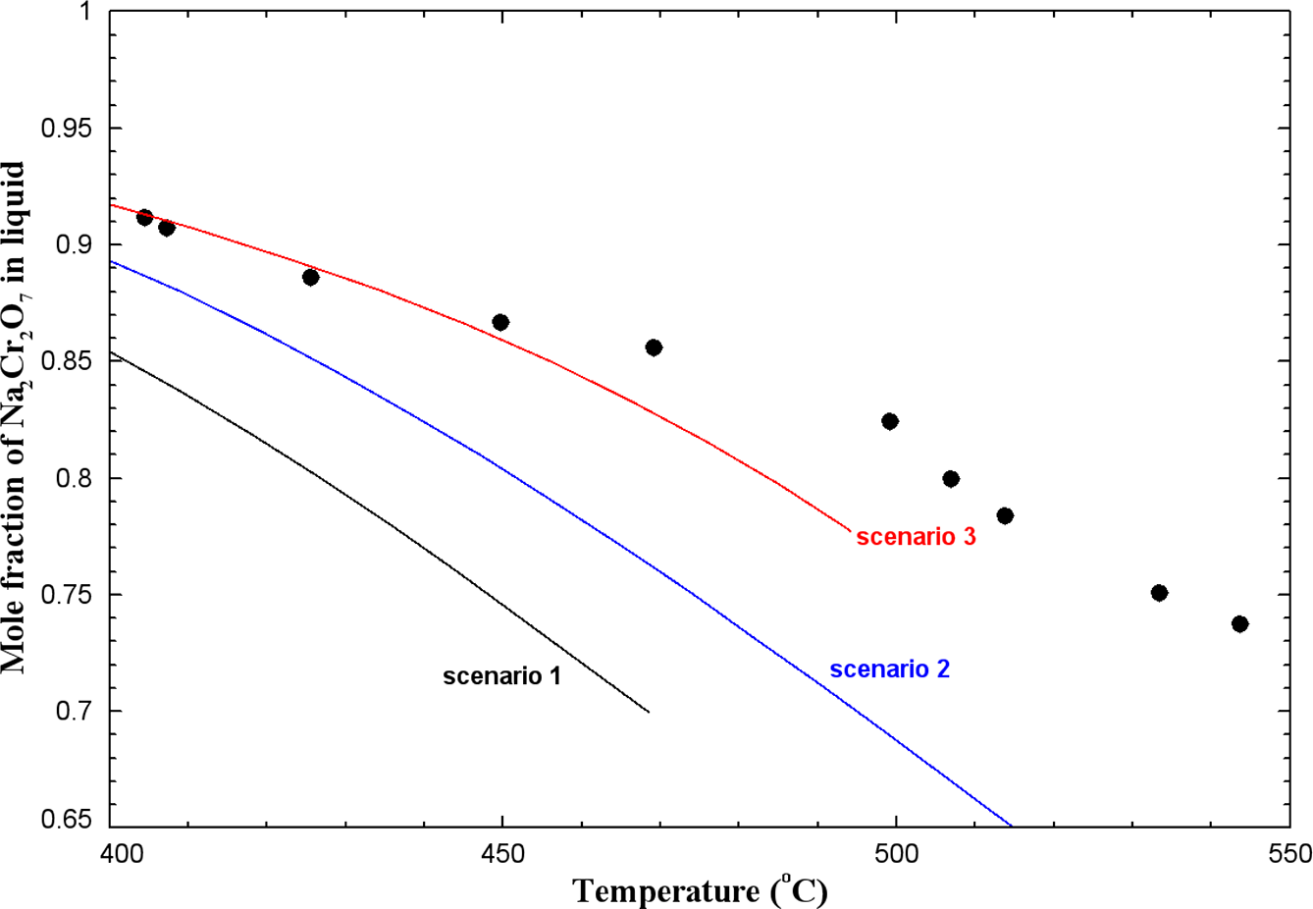
Calculated Na_2_Cr_2_O_7_ content of (Na_2_Cr_2_O_7_ + Na_2_CrO_4_) melts saturated with Cr_2_O_3_ at p­(O_2_) = 1 atm. Scenario 1: (Na_2_Cr_2_O_7_ + Na_2_CrO_4_) ideal liquid, and Δ*H*_298.15K_^°^(Cr_2_O_3_(s)) taken from the FToxid database; scenario 2: (Na_2_Cr_2_O_7_ + Na_2_CrO_4_) ideal liquid, and Δ*H*_298.15K_^°^(Cr_2_O_3_(s)) is 4 kJ/mol higher than that from the FToxid database; scenario 3: parameter of (−6100 + 10·*T*) J/mol for the (Na_2_Cr_2_O_7_ + Na_2_CrO_4_) liquid, and Δ*H*_298.15K_^°^(Cr_2_O_3_(s)) is 4 kJ/mol higher than that from the FToxid database. Experimental data from Flood and Muan^59^ (filled circle).

The calculated Na_2_Cr_2_O_7_ content of the binary melt also depends on the activities of Na_2_Cr_2_O_7_ and Na_2_CrO_4_ in this melt. In scenario 3, the Δ*H*_298.15K_^°^ value for solid Cr_2_O_3_ is identical to that for scenario 2, but a parameter of (−6100 + 10·*T*) J/mol was introduced for the (Na_2_Cr_2_O_7_ + Na_2_CrO_4_) binary liquid. This model parameter permitted to introduce positive deviations from ideality and thus better reproduce the data of ref 59 up to about 450 °C. From the three scenarios displayed in Figure 16, it can be concluded that arbitrary choices would need to be made in order to reproduce the measurements of Flood and Muan.

Although the (Na_2_Cr_2_O_7_ + Na_2_CrO_4_) liquid may exhibit negative or positive deviations from ideality, it is anticipated to be relatively close to ideality since both anions are similar.

Finally, in the present work, it was assumed to be ideal (that is, Δ*g*_Na_2_/(CrO_4_)(Cr_2_O_7_)_ = 0). The predicted phase diagram in air (p­(O_2_) = 0.21 atm) is shown in Figure 17. Measuring this phase diagram would permit to best calibrate the binary liquid.

**Figure 17:**
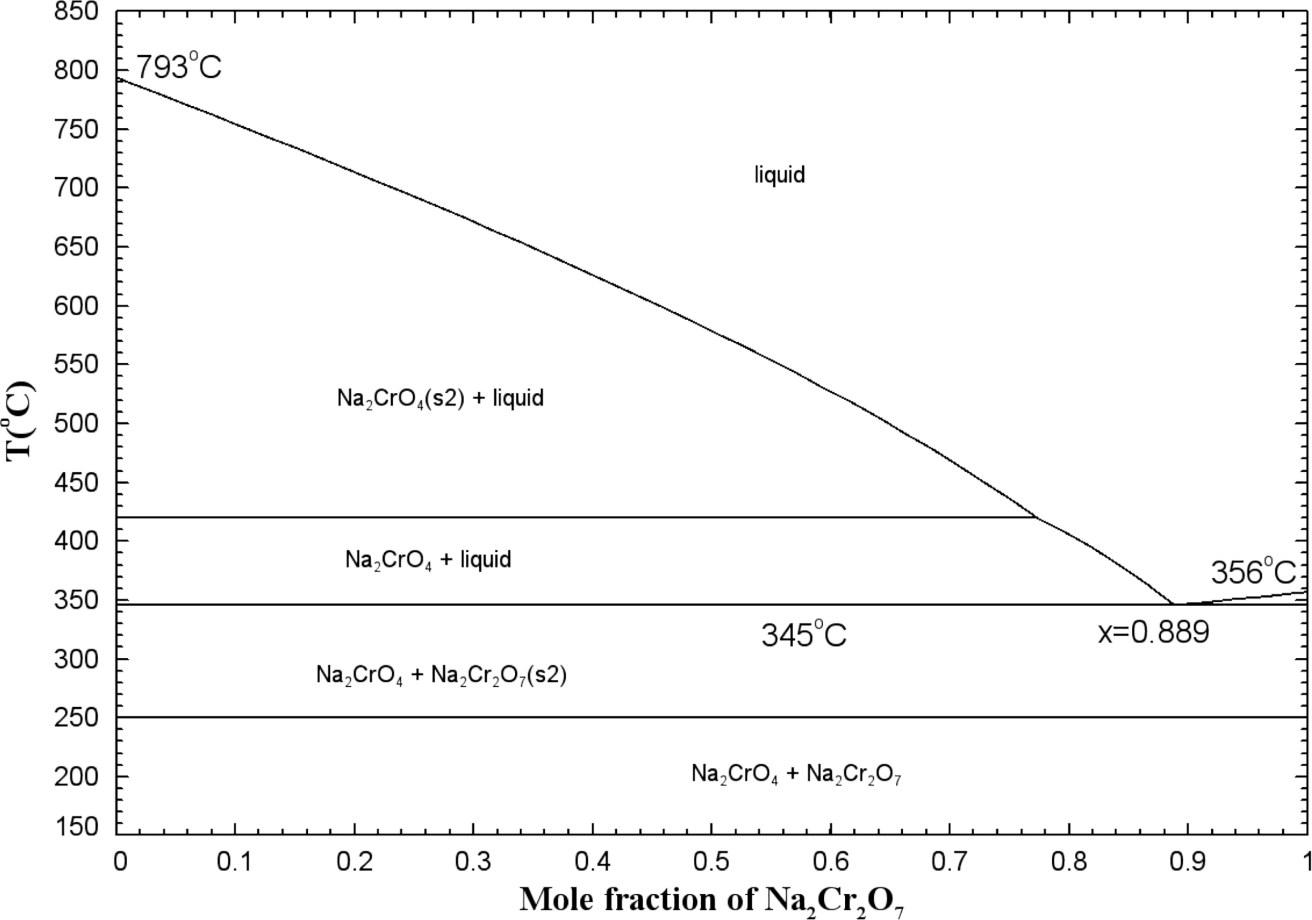
Calculated (predicted) (Na_2_CrO_4_ + Na_2_Cr_2_O_7_) phase diagram in air (p­(O_2_) = 0.21 atm), for an ideal liquid.

Note that Flood and Muan^59^ also measured the CrO_4_ content of (Na_2_Cr_2_O_7_ + K_2_Cr_2_O_7_ + Na_2_CrO_4_ + K_2_CrO_4_) melts at 662 °C over the entire range of Na/K molar ratios, using the same experimental protocol as described previously. Those data are not presented here since the same problem as for (Na_2_Cr_2_O_7_ + Na_2_CrO_4_) was encountered.

#### 5.1.11 The (Na_2_Cr_2_O_7_ + K_2_Cr_2_O_7_) System

The phase diagram has been investigated using a combination of thermal analysis and visual observation^60^ and the heating and cooling curves method^61^. Lehrman et al.^60^ reported the presence of a eutectic plateau from 22.9 to 84.7 mol % K_2_Cr_2_O_7_ at a temperature of about 303 to 306 °C, based on thermal analysis. Palkin and Bokhovkin^61^ reported a eutectic at 42.5 mol % K_2_Cr_2_O_7_ and 300 °C. The eutectic is calculated at 45.8 mol % K_2_Cr_2_O_7_ and 300 °C. The data of refs 60,61 are somewhat different but were both considered in our model.

The high-temperature allotropes Na_2_Cr_2_O_7(S2)_ and K_2_Cr_2_O_7(S2)_ have different crystal structures (Table S1 in the Supporting Information). Consequently, aP22 and K_2_Cr_2_O_7_(s.s) were introduced as two terminal high-temperature solid solutions. At the eutectic temperature of 300 °C, the solid solubility limits are calculated to be 24.4 and 82.9 mol % K_2_Cr_2_O_7_, respectively. These agree reasonably well with the experimental values of 25.0 and 79.0 mol % K_2_Cr_2_O_7_ reported by Palkin and Bokhovkin.^61^

According to Table S1 in the Supporting Information, the low-temperature allotropes Na_2_Cr_2_O_7(S1)_ and K_2_Cr_2_O_7(S1)_ exhibit an identical crystal structure and space group *P*1̅. A low-temperature solid solution (aP44) was therefore introduced across the whole composition range. A significant positive regular parameter of +20,000.0 J/mol was arbitrarily included in this solid solution to reduce its calculated extent and permit the reproduction of the high-temperature data of refs 60,61 with the two high-temperature terminal solid solutions. A miscibility gap (related to aP44) was calculated at low temperatures.

Due to the similarity of the cationic radii,^62^ the binary liquid was assumed to be ideal (i.e., Δ*g*_NaK/Cr_2_O_7__ = 0). The calculated (Na_2_Cr_2_O_7_ + K_2_Cr_2_O_7_) phase diagram in air (p­(O_2_) = 0.21 atm) is shown in Figure 18 along with the available data.

**Figure 18:**
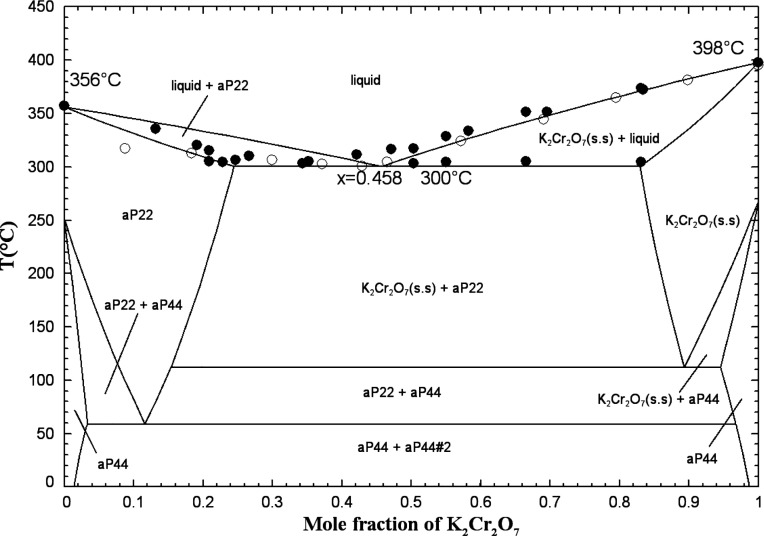
Calculated (Na_2_Cr_2_O_7_ + K_2_Cr_2_O_7_) phase diagram in air (p­(O_2_) = 0.21 atm). Experimental data from Lehrman et al.^60^ (filled circle) and Palkin and Bokhovkin^61^ (open circle, extracted from ref 63).

### 5.2 Chromate-Based Common-Cation Ternary Subsystems

#### 5.2.1 The (Na_2_CO_3_ + Na_2_SO_4_ + Na_2_CrO_4_) System

Bergman and Sanzharov^64^ studied the liquidus surface of the (Na_2_CO_3_ + Na_2_SO_4_ + Na_2_CrO_4_) system using the visual-polythermal method. Eight isoplethal sections were measured. However, the corresponding data were not provided, and the authors only reported a smoothed liquidus projection. Thus, the intersections between the eight isoplethal sections and the drawn isotherms were extracted graphically. The calculated liquidus projection is shown in Figure 19 along with the graphically extracted data points.

**Figure 19:**
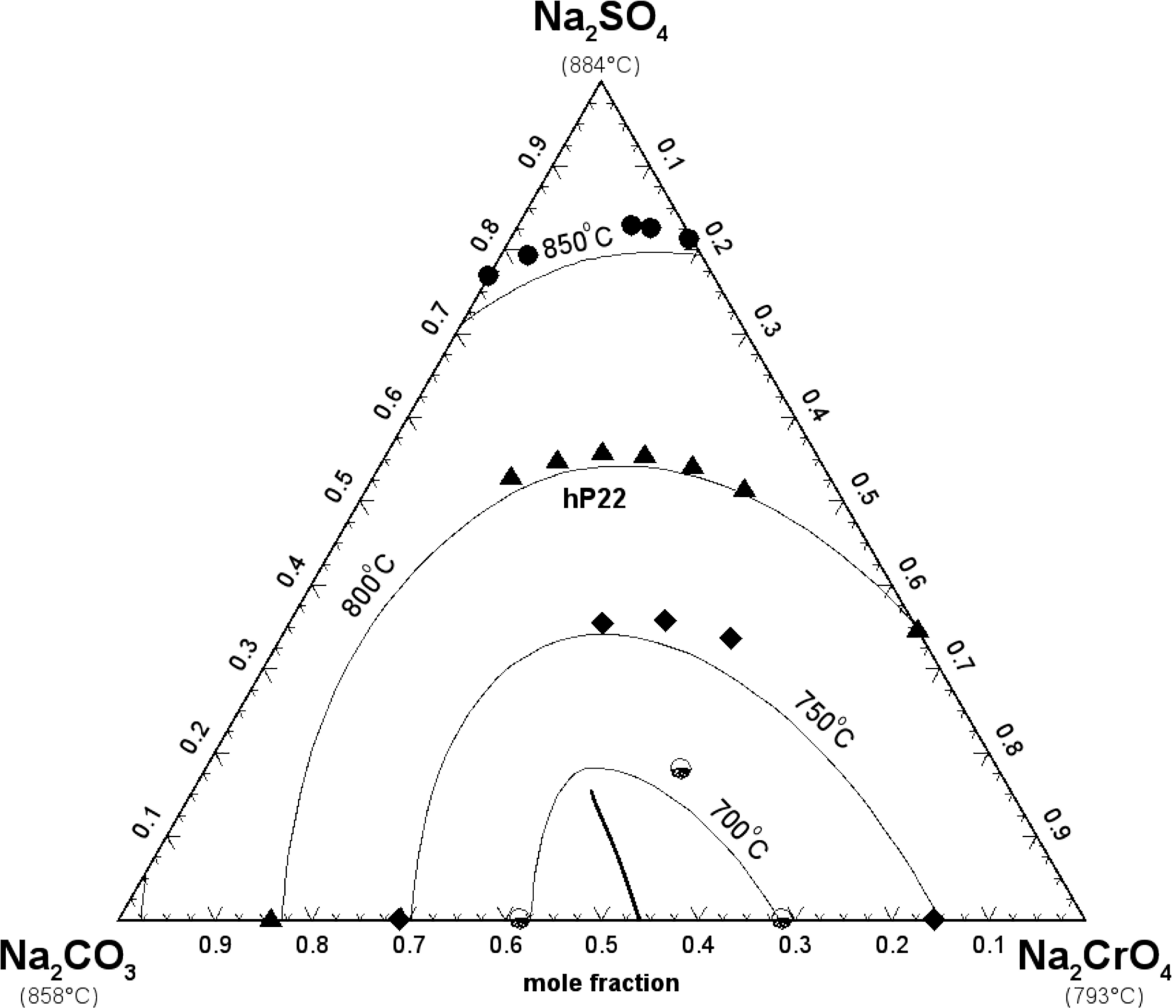
Calculated liquidus projection of the (Na_2_CO_3_ + Na_2_SO_4_ + Na_2_CrO_4_) system. Experimental data for isotherms (filled circle: 850 °C, filled triangle: 800 °C, filled diamond: 750 °C, down-filled circle: 700 °C) were extracted graphically from the smoothed liquidus projection of Bergman and Sanzharov.^64^

In a previous study,^16^ the binary subsystem (Na_2_CO_3_ + Na_2_SO_4_) was modeled, and the optimized model parameters were directly used in this study. No ternary excess parameter was introduced in the liquid model. Overall, agreement was satisfactory owing to the uncertainty related to the graphical extraction of the intersection points from a smoothed liquidus projection. As shown in Figure 19, the measured composition of the binary mixture (Na_2_CO_3_ + Na_2_SO_4_) at 850 °C is in disagreement with the calculated composition, which was based on experimental data referenced in ref 16.

Bergman and Sanzharov^64^ reported a ternary eutectic reaction at 648 °C and liquid composition of (46 mol % Na_2_CO_3_ + 3 mol % Na_2_SO_4_ + 51 mol % Na_2_CrO_4_). The hexagonal solid solution hP22 is calculated to precipitate from the liquid phase over the whole range of compositions. Close to the (Na_2_CO_3_ + Na_2_CrO_4_) binary subsystem, there is a calculated univariant line corresponding to a solid–solid miscibility gap.

#### 5.2.2 The (K_2_CO_3_ + K_2_SO_4_ + K_2_CrO_4_) System

Sanzharov and Bergman^42^ investigated the liquidus surface of the (K_2_CO_3_ + K_2_SO_4_ + K_2_CrO_4_) system by the visual-polythermal method. Ten isoplethal sections were studied but measurements were reported only for two of them. The calculated liquidus projection is presented in Figure 20, and the comparison of the two calculated isoplethal sections with the available experimental data is displayed in Figures 21 and 22. The previous model of the binary subsystem (K_2_CO_3_ + K_2_SO_4_) is described in ref 16; all optimized model parameters were directly used in this study. One small ternary excess parameter was introduced in the liquid model (Table S3 in the Supporting Information). The data shown in Figure 21 are in better agreement with the calculated solidus, while the data shown in Figure 22 are in better agreement with the calculated liquidus. In Figures 21 and 22, red lines are predictions made only from the optimized model parameters for the three common-cation binary subsystems.

**Figure 20:**
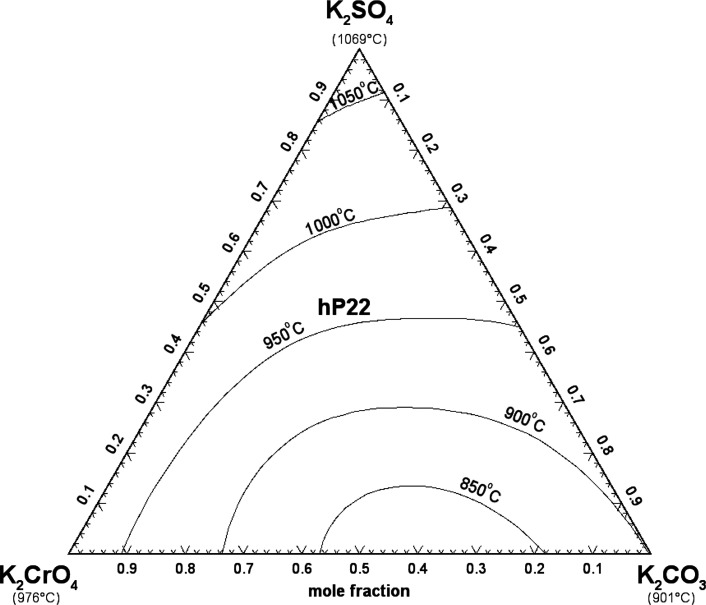
Calculated liquidus projection of the (K_2_CO_3_ + K_2_SO_4_ + K_2_CrO_4_) system.

**Figure 21:**
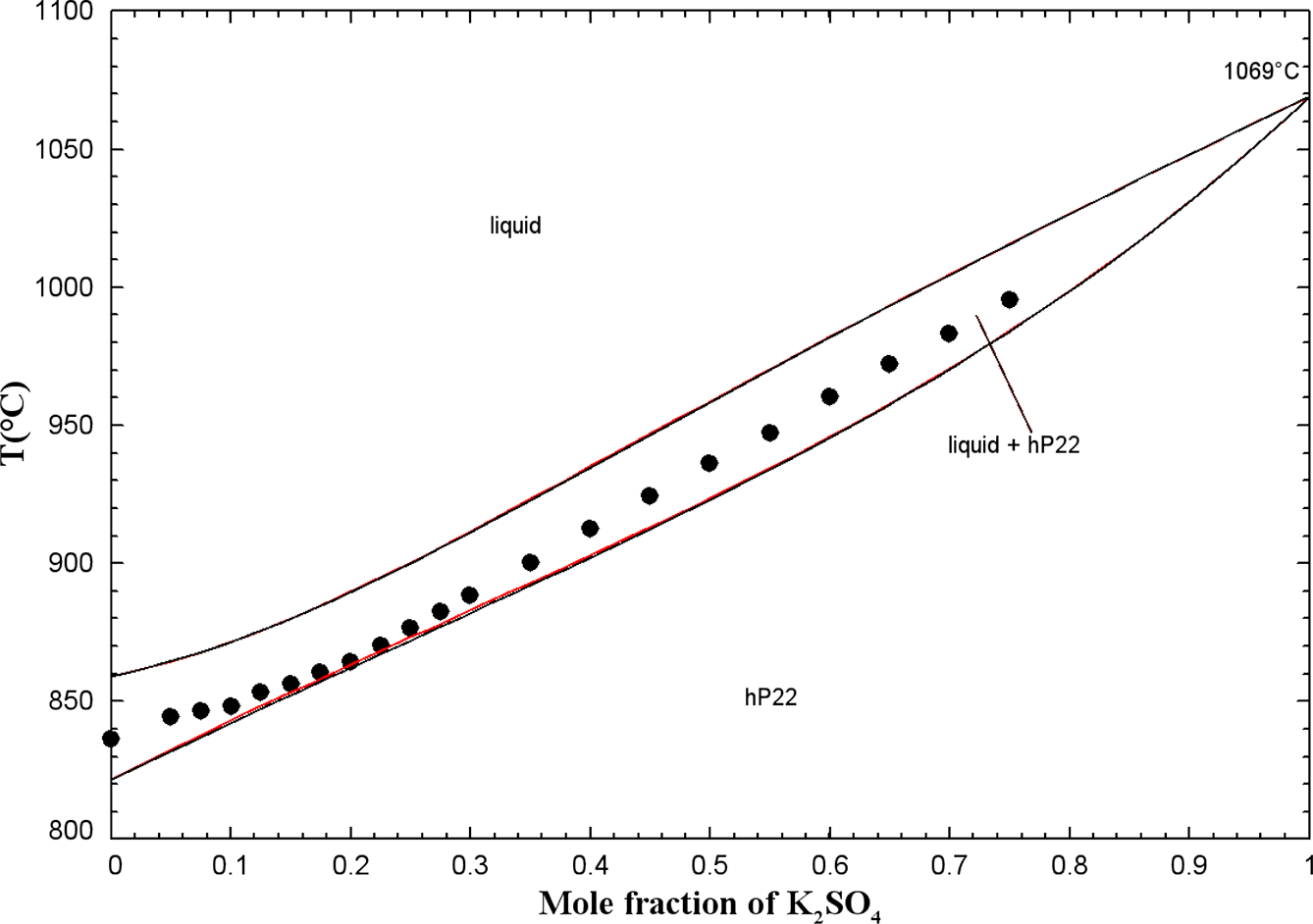
Calculated isoplethal section in the (K_2_CO_3_ + K_2_SO_4_ + K_2_CrO_4_) system ((K_2_CO_3_)_0.85_(K_2_CrO_4_)_0.15_-K_2_SO_4_). Black lines: final calculations (with one ternary excess parameter); red lines: predictions (without any ternary excess parameter). Experimental data from ref 42 (filled circle).

**Figure 22:**
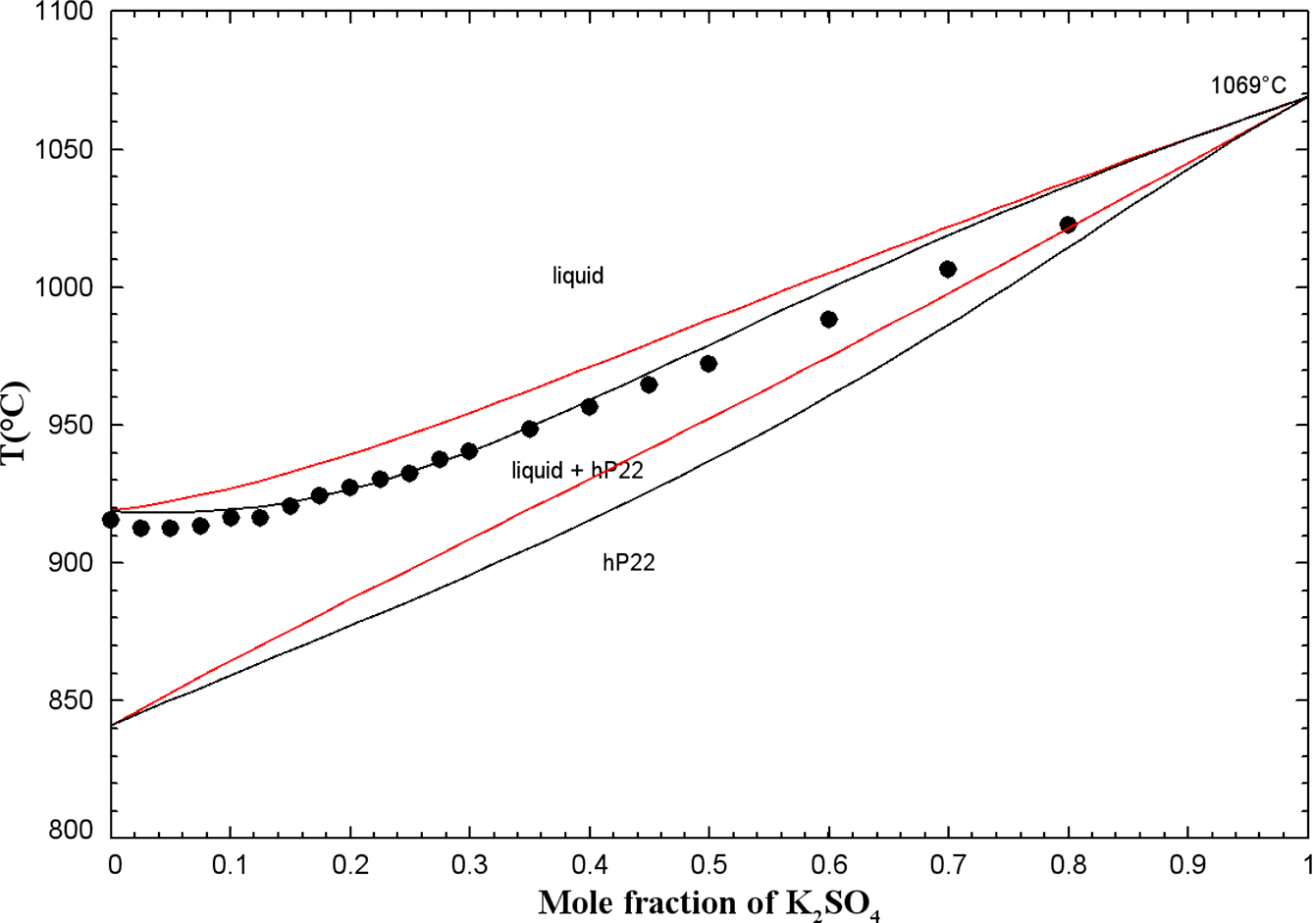
: Calculated isoplethal section in the (K_2_CO_3_ + K_2_SO_4_ + K_2_CrO_4_) system ((K_2_CO_3_)_0.20_(K_2_CrO_4_)_0.80_-K_2_SO_4_). Black lines: final calculations (with one ternary excess parameter); red lines: predictions (without any ternary excess parameter). Experimental data from ref 42 (filled circle).

Sanzharov and Bergman^42^ reported a ternary eutectic reaction at 786 °C and liquid composition of (62 mol % K_2_CO_3_ + 1.5 mol % K_2_SO_4_ + 36.5 mol % K_2_CrO_4_). This is not possible since Figure 20 shows that the hexagonal solid solution hP22 is calculated to precipitate from the liquid phase over the whole range of compositions. Therefore, there are no calculated ternary invariant points.

#### 5.2.3 The (KCl + K_2_CO_3_ + K_2_CrO_4_) System

Currently, there are no reported ternary phase diagram data for the (KCl + K_2_CO_3_ + K_2_CrO_4_) system. Figure 23 displays the calculated liquidus projection. No ternary excess parameter was introduced in the liquid model. Thus, only the optimized model parameters for the three common-cation binary subsystems were used to predict the liquidus projection. The (KCl + K_2_CO_3_) binary subsystem was previously modeled,^17^ and the optimized model parameters were directly used in the present study.

**Figure 23:**
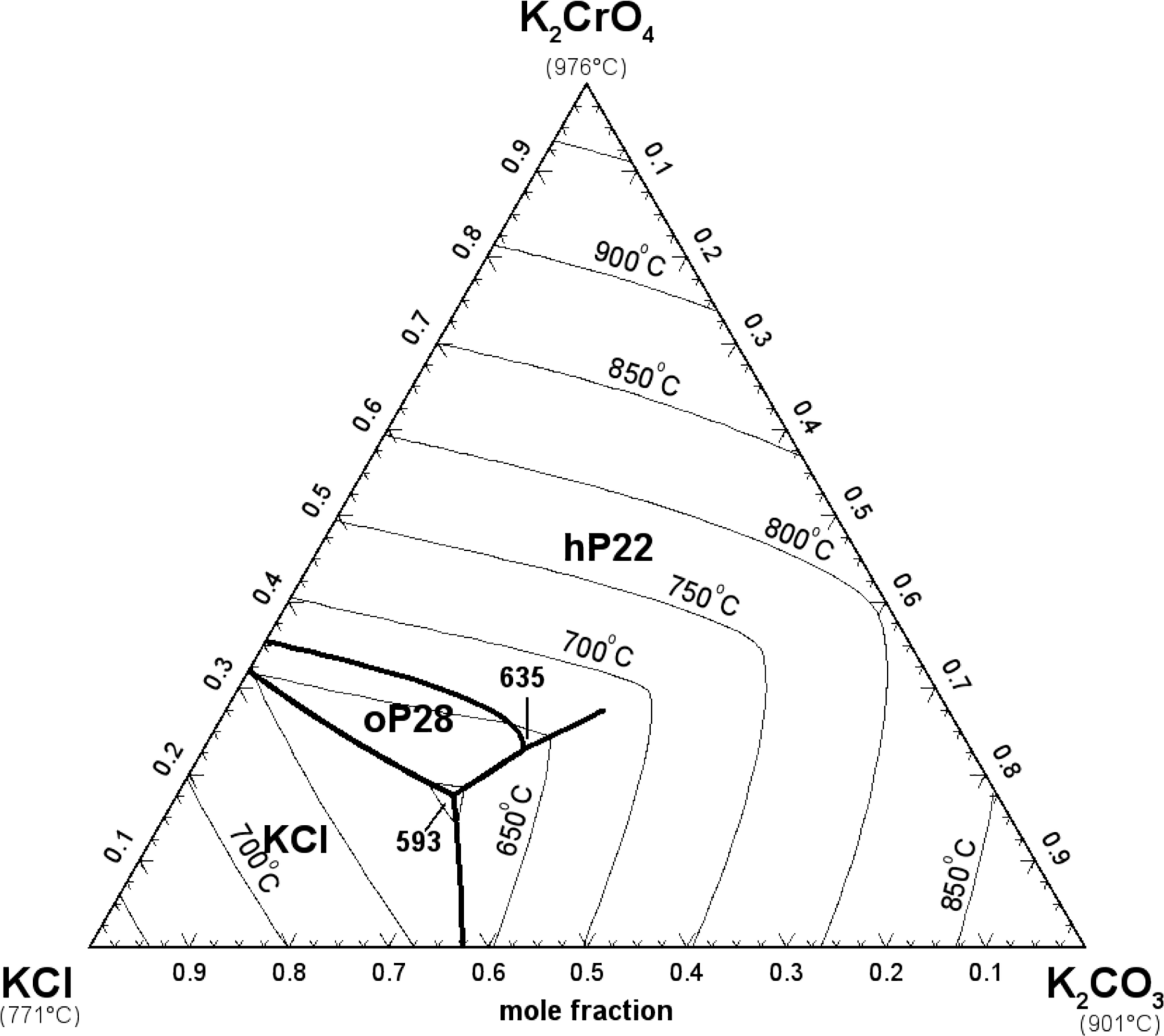
Calculated liquidus projection of the (KCl + K_2_CO_3_ + K_2_CrO_4_) system.

The ternary eutectic reaction liquid = KCl_(S)_ + hP22 + oP28 is predicted at 593 °C and liquid composition of (54.7 mol % KCl + 27.8 mol % K_2_CO_3_ + 17.5 mol % K_2_CrO_4_), and the ternary quasi-peritectic reaction liquid + hP22 = hP22 + oP28 is predicted at 635 °C and liquid composition of (45.1 mol % KCl + 32.1 mol % K_2_CO_3_ + 22.8 mol % K_2_CrO_4_).

In this work, a ternary mixture consisting of (55.0 mol % KCl + 28.0 mol % K_2_CO_3_ + 17.0 mol % K_2_CrO_4_) was investigated by DSC-TGA over the temperature range 200–645 °C. The corresponding DSC thermogram is shown in Figure 24. There is a single thermal arrest around 596 °C, which represents the average of the two experimental temperatures from the second and third heating runs. This experimental ternary eutectic is, therefore, very well predicted by our model. This is an important feature as the formation of a liquid at low temperatures (such as ternary eutectics) may lead to catastrophic corrosion.

**Figure 24:**
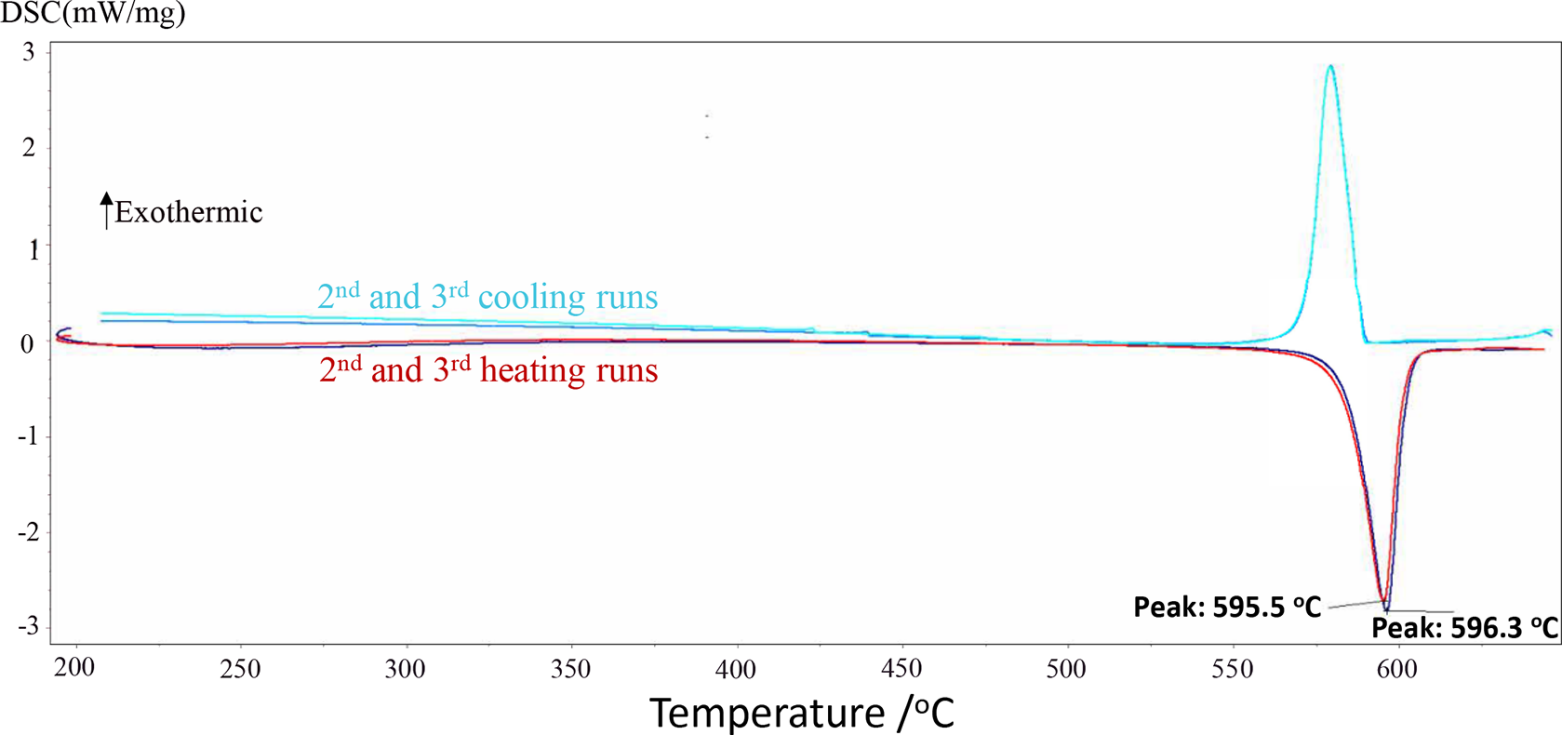
DSC thermogram for the ternary mixture (55.0 mol % KCl + 28.0 mol % K_2_CO_3_ + 17.0 mol % K_2_CrO_4_) (second and third heating/cooling cycles only).

### 5.3 Chromate-Based Ternary and Quaternary Reciprocal Subsystems

#### 5.3.1 The (Na_2_SO_4_ + K_2_SO_4_ + Na_2_CrO_4_ + K_2_CrO_4_) System

Figure 25 displays the calculated liquidus projection of the ternary reciprocal system (Na_2_SO_4_ + K_2_SO_4_ + Na_2_CrO_4_ + K_2_CrO_4_). In this figure, the four apices of the reciprocal square represent the pure salts (Na_2_SO_4_, K_2_SO_4_, Na_2_CrO_4_, and K_2_CrO_4_). Compositions are expressed as equivalent fractions of cations and anions, so that tie-lines are straight lines. In Figure 25, the horizontal axis represents the cationic molar ratio *n*_K_/(*n*_Na_ + *n*_K_), while the vertical axis represents the anionic molar ratio *n*_CrO_4__/(*n*_SO_4__ + *n*_CrO_4__). No ternary reciprocal parameter was introduced in the liquid model. Thus, only the optimized model parameters for the four common-ion binary subsystems were used to make predictions in the (Na_2_SO_4_ + K_2_SO_4_ + Na_2_CrO_4_ + K_2_CrO_4_) system. The (Na_2_SO_4_ + K_2_SO_4_) binary subsystem was previously modeled,^15^ and the optimized model parameters were directly used in this study. As shown in Figure 25, the hexagonal solid solution hP22 is calculated to precipitate from the liquid phase over the whole range of compositions.

**Figure 25:**
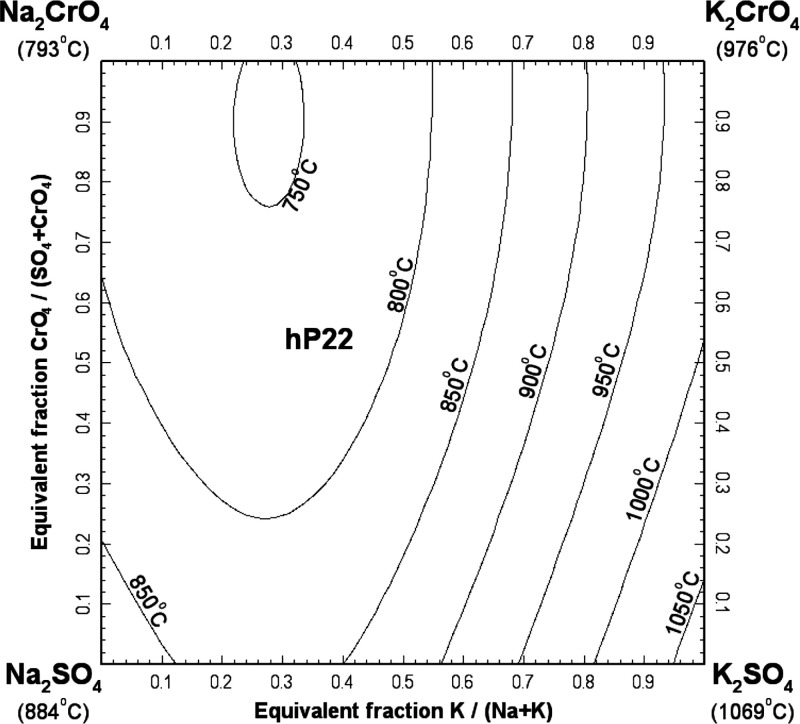
Calculated liquidus projection of the (Na_2_SO_4_ + K_2_SO_4_ + Na_2_CrO_4_ + K_2_CrO_4_) system.

Flach^43^ used the method of cooling curves to measure the section (K_3_Na­(SO_4_)_2_ + K_3_Na­(CrO_4_)_2_), extending from the sulfur-glaserite to the chrome-glaserite which were both modeled with the hP14 nonstoichiometric compound. Figure 26 displays the calculated section along with the available data. Agreement is excellent. Three different solid solutions (hP22, hP14, and oP28) can precipitate. Note that several calculated thermal arrests (in particular, the solidus of the hexagonal solid solution hP22) were not observed experimentally by Flach.^43^

**Figure 26:**
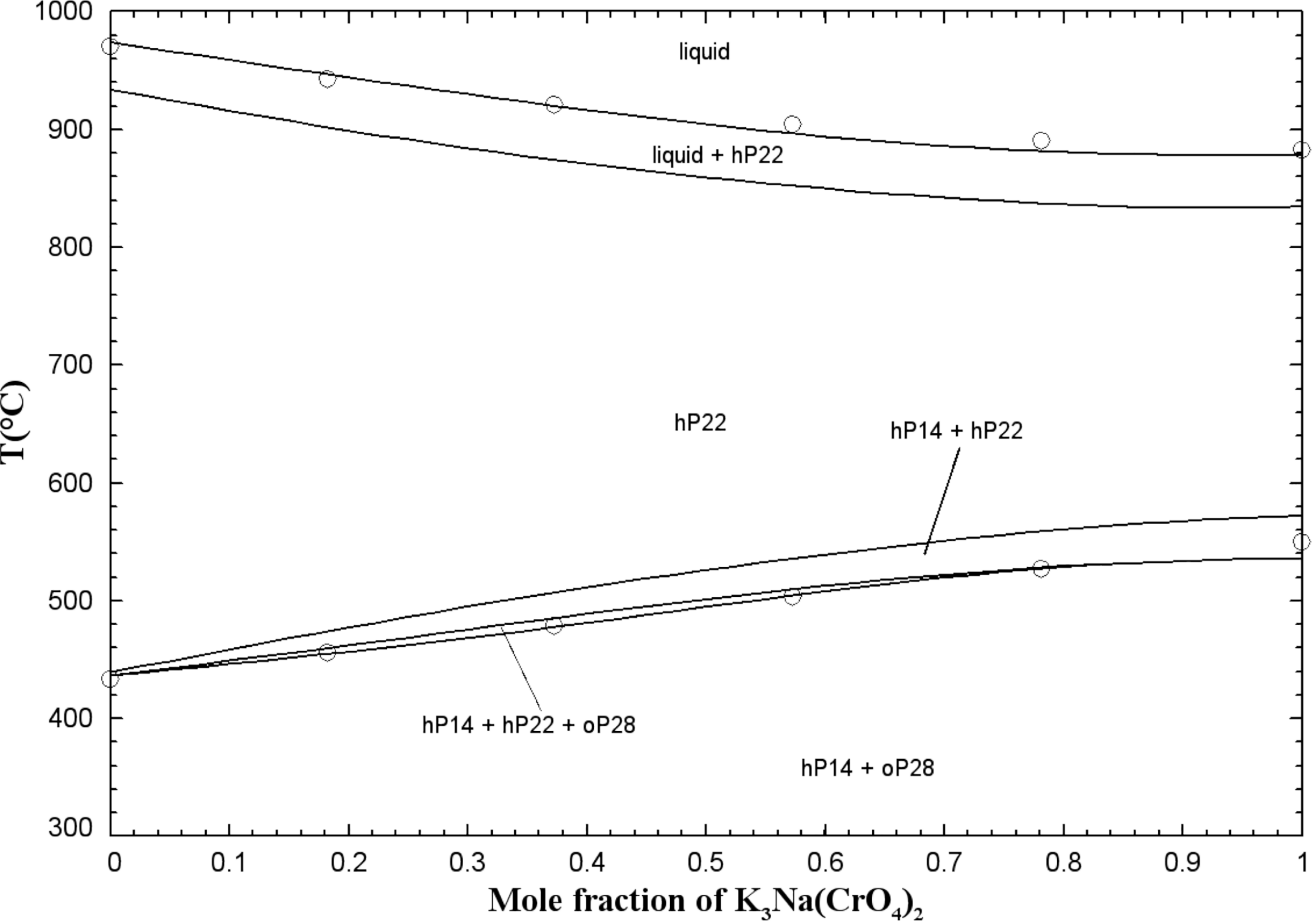
Calculated (K_3_Na­(SO_4_)_2_ + K_3_Na­(CrO_4_)_2_) section in the (Na_2_SO_4_ + K_2_SO_4_ + Na_2_CrO_4_ + K_2_CrO_4_) system. Experimental data from Flach^43^ (open circle).

Bergman and Sanzharov^65^ measured the (Na_2_SO_4_ + K_2_CrO_4_) diagonal section using the visual-polythermal method, and reported a marked minimum at 32.5 mol % K_2_CrO_4_ and 780 °C. No measurements were reported, and therefore the data points were extracted graphically from the smoothed liquidus projection provided by the authors for the (Na_2_CO_3_ + Na_2_SO_4_ + K_2_CrO_4_) system. The calculated (Na_2_SO_4_ + K_2_CrO_4_) phase diagram is compared to the graphically extracted data points in Figure 27. Agreement is very satisfactory.

**Figure 27:**
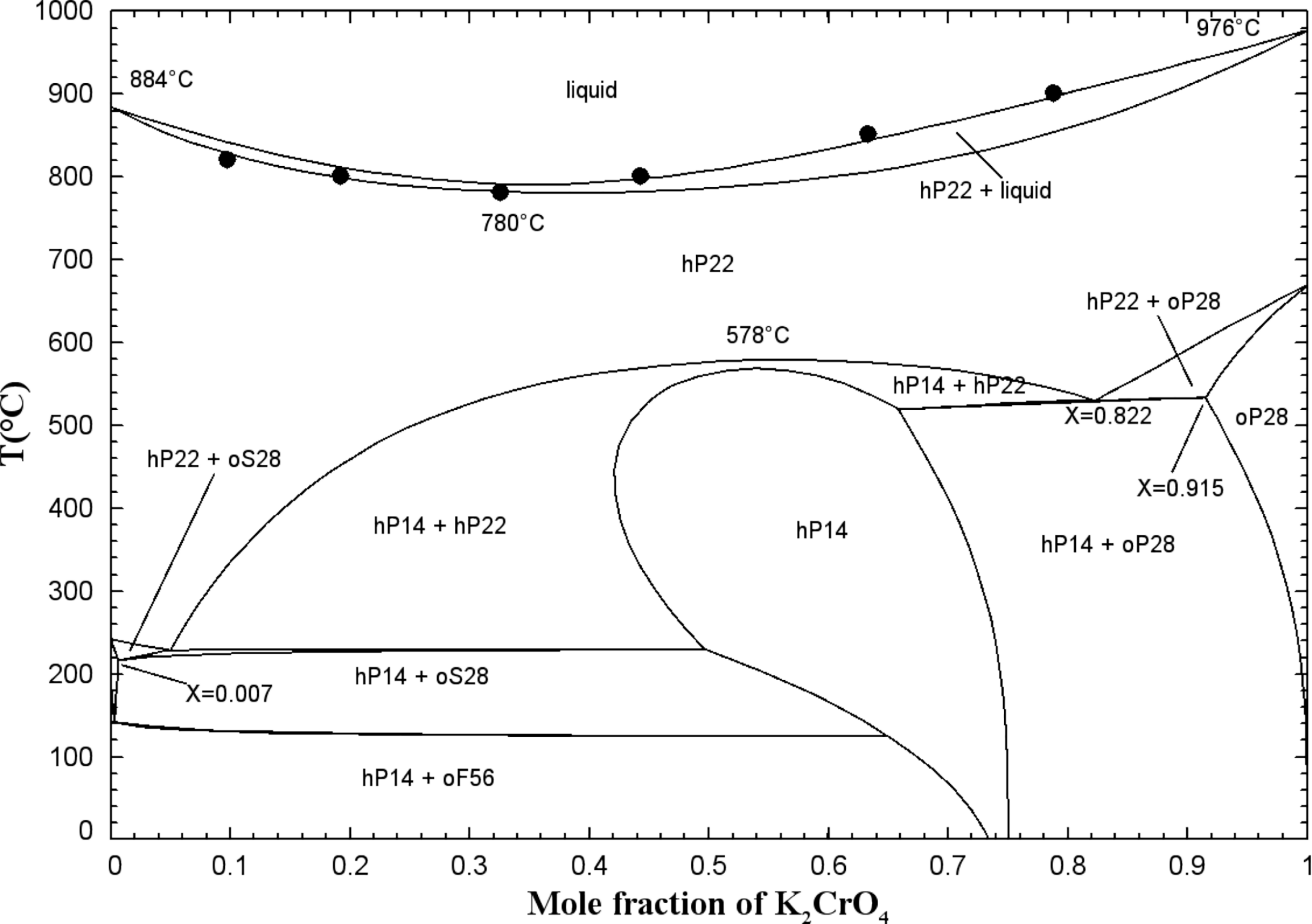
Calculated (Na_2_SO_4_ + K_2_CrO_4_) section in the (Na_2_SO_4_ + K_2_SO_4_ + Na_2_CrO_4_ + K_2_CrO_4_) system. Experimental data (filled circle) were extracted graphically from the smoothed liquidus projection of Bergman and Sanzharov^65^ for the (Na_2_CO_3_ + Na_2_SO_4_ + K_2_CrO_4_) system.

#### 5.3.2 The (NaCl + KCl + Na_2_CrO_4_ + K_2_CrO_4_) System

Figure 28 shows the calculated liquidus projection of the (NaCl + KCl + Na_2_CrO_4_ + K_2_CrO_4_) system. No ternary reciprocal parameter was introduced in the liquid model. The (NaCl + KCl) binary subsystem was previously modeled,^22^ and the optimized model parameters were directly used in this study. Bergman and Trunin^66^ employed the visual-polythermal method to investigate 15 isoplethal sections, which permitted them to construct the liquidus surface of this system. Unfortunately, these data points have limited accuracy since only their measured liquidus temperatures were reported and their ternary compositions had to be extracted graphically from a smoothed liquidus projection provided by the authors. Each isoplethal section investigated by Bergman and Trunin^66^ consisted of a common-cation binary mixture to which was added a pure salt or another common-cation binary mixture.

**Figure 28:**
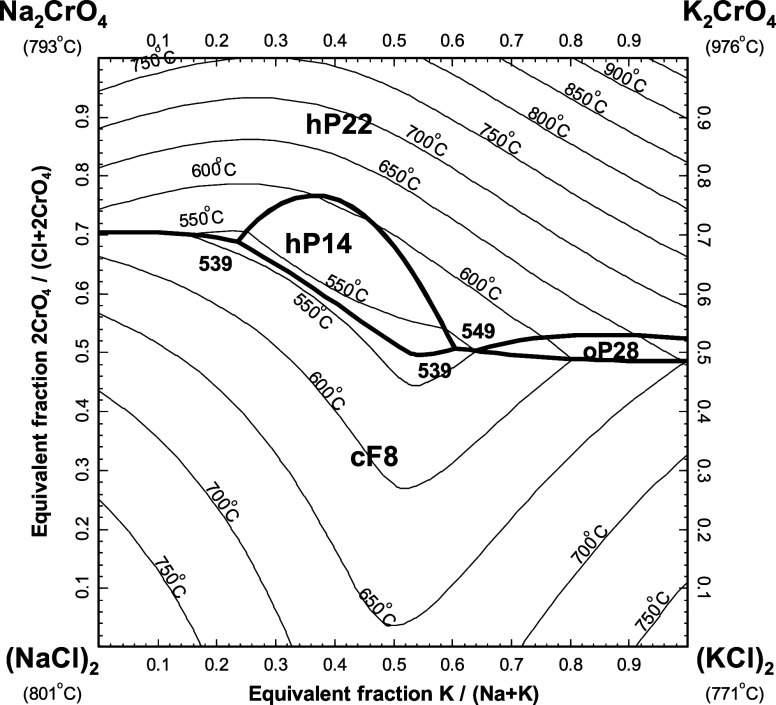
Calculated liquidus projection of the (NaCl + KCl + Na_2_CrO_4_ + K_2_CrO_4_) system.

For the sake of comparison between the calculations and the measurements, there were enough ternary compositions studied for only six of the 15 isoplethal sections. For each of those six isoplethal sections, the composition of the binary mixture was assessed as accurately as possible by considering simultaneously the intersection point with the relevant side of the reciprocal square along with the graphically extracted ternary compositions investigated by Bergman and Trunin.^66^

As an illustration, Figures 29 and 30 present a comparison of two calculated isoplethal sections with the corresponding measurements. The four other isoplethal sections can be found in the Supporting Information (Figures S2–S5). Overall, the agreement was satisfactory owing to the uncertainty related to the graphical extraction of the compositions from a smoothed liquidus projection.

**Figure 29:**
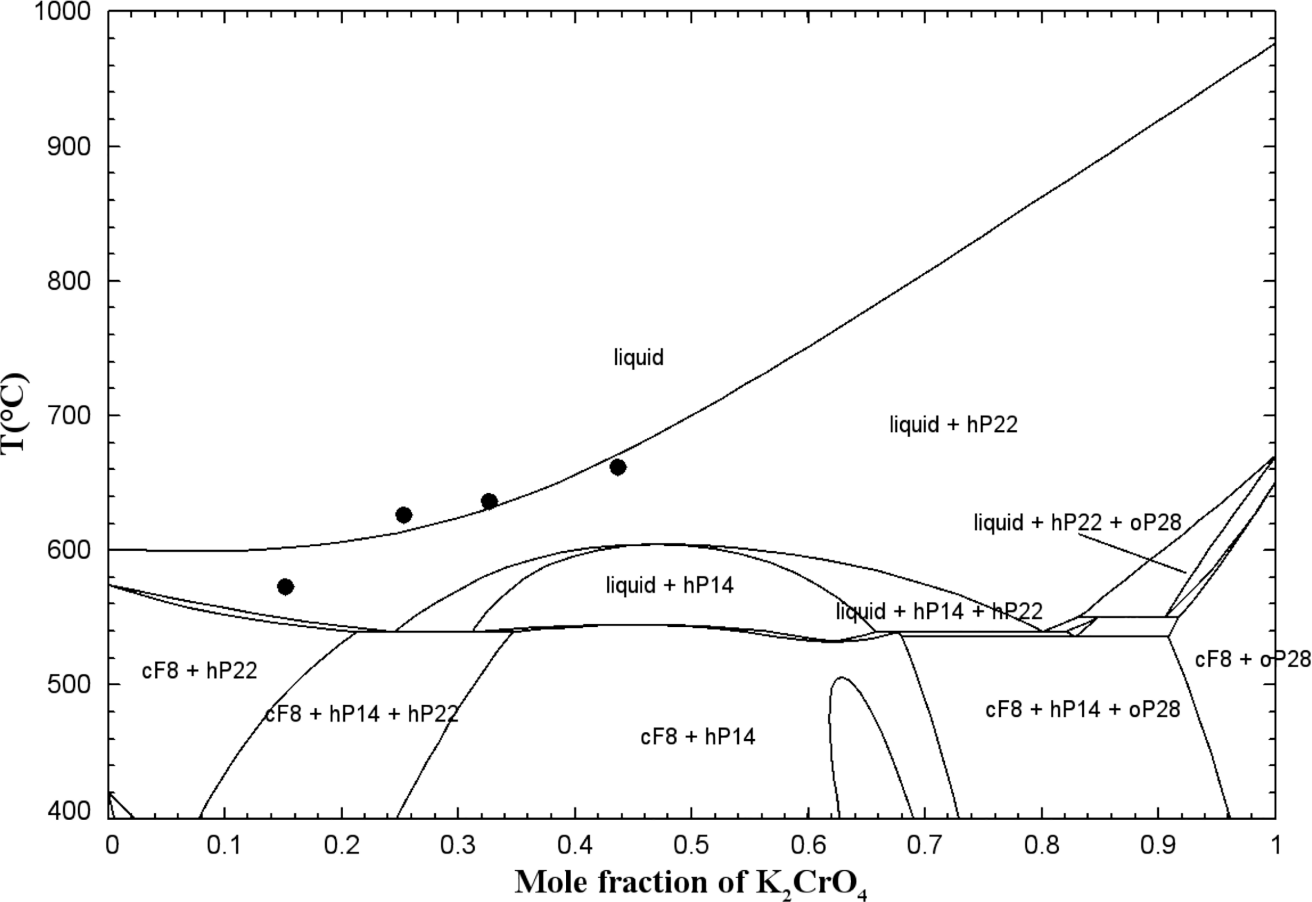
Calculated isoplethal section in the (NaCl + KCl + Na_2_CrO_4_ + K_2_CrO_4_) system ((Na_2_CrO_4_)_0.743_(Na_2_Cl_2_)_0.257_-K_2_CrO_4_, section III). Experimental data extracted graphically from the liquidus projection of Bergman and Trunin^66^ (filled circle).

**Figure 30:**
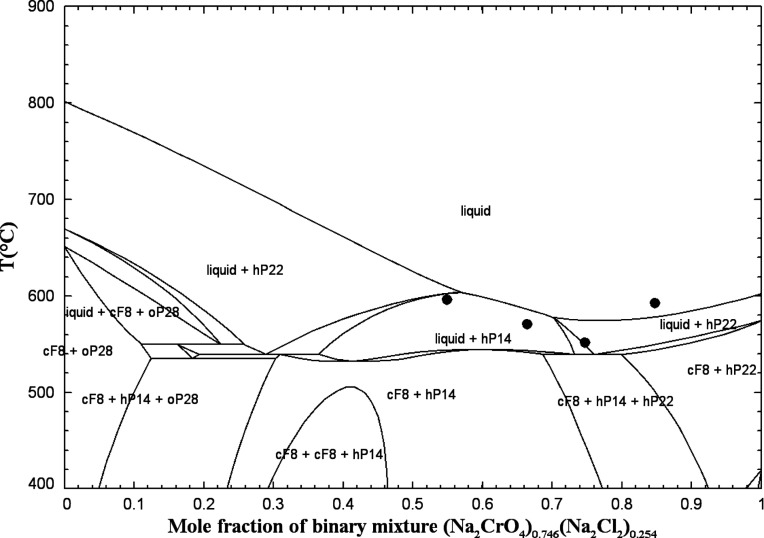
Calculated isoplethal section in the (NaCl + KCl + Na_2_CrO_4_ + K_2_CrO_4_) system ((K_2_CrO_4_)_0.746_(K_2_Cl_2_)_0.254_-(Na_2_CrO_4_)_0.746_(Na_2_Cl_2_)_0.254_, section XII). Experimental data extracted graphically from the liquidus projection of Bergman and Trunin^66^ (filled circle).

#### 5.3.3 The (Na_2_CO_3_ + K_2_CO_3_ + Na_2_CrO_4_ + K_2_CrO_4_) System

Bergman and Sanzharov^65^ used the visual-polythermal technique to measure the (Na_2_CO_3_ + K_2_CrO_4_) diagonal section. They reported a eutectic point at 37.5 mol % K_2_CrO_4_ and 635 °C. Three small ternary reciprocal parameters were included in the liquid phase to best reproduce those data (see Table S3 in the Supporting Information). Again, no measurements were provided by the authors, and thus the data points were extracted graphically from their smoothed liquidus projection of the (Na_2_CO_3_ + Na_2_SO_4_ + K_2_CrO_4_) system. The calculated liquidus projection of the (Na_2_CO_3_ + K_2_CO_3_ + Na_2_CrO_4_ + K_2_CrO_4_) ternary reciprocal system is displayed in Figure 31.

**Figure 31:**
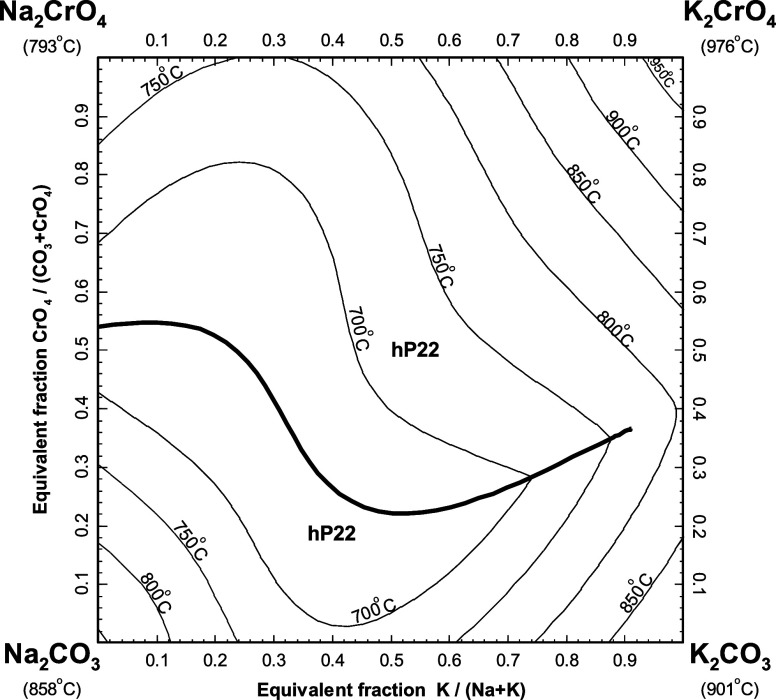
Calculated liquidus projection of the (Na_2_CO_3_ + K_2_CO_3_ + Na_2_CrO_4_ + K_2_CrO_4_) system.

The hexagonal solid solution hP22 is calculated to precipitate from the liquid phase over the whole range of compositions. The calculated univariant line does not reach the (K_2_CO_3_ + K_2_CrO_4_) binary subsystem since this system exhibits a minimum (see Figure 6).

Figure 32 displays the calculated (Na_2_CO_3_ + K_2_CrO_4_) phase diagram in air (p­(O_2_) = 0.21 atm) along with the data points extracted graphically and the calculated isobar at 1 atm.

**Figure 32:**
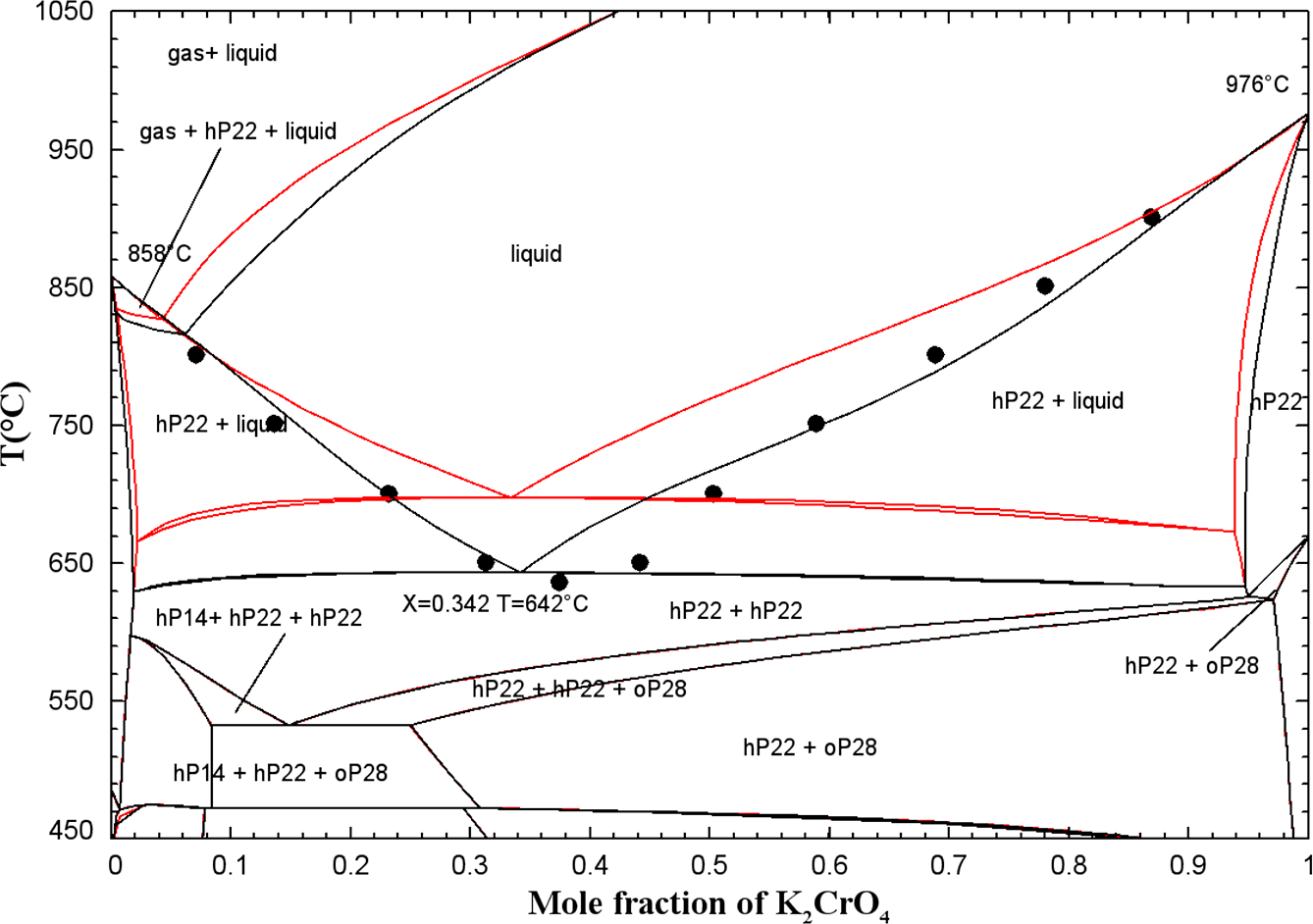
Calculated (Na_2_CO_3_ + K_2_CrO_4_) phase diagram in air (p­(O_2_) = 0.21 atm). Black lines: final calculations (with three ternary reciprocal parameters); red lines: predictions (without any ternary reciprocal parameter). Experimental data (filled circle) were extracted graphically from the smoothed liquidus projection of Bergman and Sanzharov^65^ for the (Na_2_CO_3_ + Na_2_SO_4_ + K_2_CrO_4_) system.

#### 5.3.4 The (Na_2_CO_3_ + Na_2_SO_4_ + Na_2_CrO_4_ + K_2_CO_3_ + K_2_SO_4_ + K_2_CrO_4_) Quaternary Reciprocal Subsystem

Bergman and Sanzharov^65^ used the visual-polythermal technique to investigate the (Na_2_CO_3_ + Na_2_SO_4_ + K_2_CrO_4_) system and measured nine isoplethal sections. Experimental data were not provided by the authors, and only a smoothed liquidus projection was reported. Therefore, the intersections between the nine isoplethal sections and the drawn isotherms were extracted graphically. Figure 33 shows the calculated (predicted) liquidus projection of the (Na_2_CO_3_ + Na_2_SO_4_ + K_2_CrO_4_) system along with the extracted data points.

**Figure 33:**
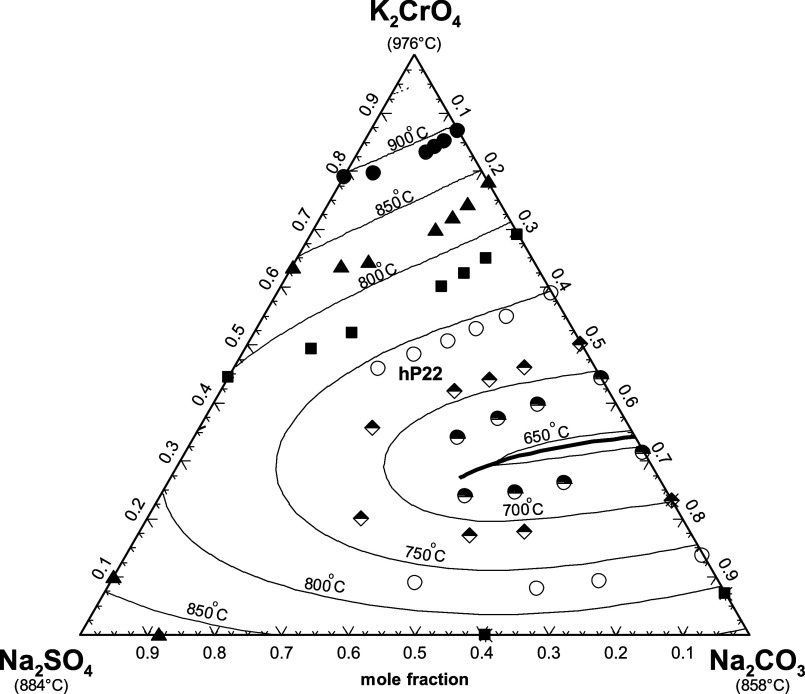
Calculated liquidus projection of the (Na_2_CO_3_ + Na_2_SO_4_ + K_2_CrO_4_) system. Experimental data for isotherms (filled circle: 900 °C, filled triangle: 850 °C, filled square: 800 °C, open circle: 750 °C, up-filled diamond: 700 °C, and up-filled circle: 650 °C) were extracted graphically from the smoothed liquidus projection of Bergman and Sanzharov.^65^

The reciprocal ternary subsystem (Na_2_CO_3_ + K_2_CO_3_ + Na_2_SO_4_ + K_2_SO_4_) was modeled earlier,^16^ and the optimized model parameters were directly used in this study. Note that no higher-order model parameters than ternary reciprocal parameters are available in the MQMQA. Sometimes, significant shifts were observed between the measured and calculated isotherms. This may be partly attributed to the limited accuracy of the data points extracted graphically. Also, the measured compositions of the (Na_2_CO_3_ + Na_2_SO_4_) common-cation binary mixtures at 800 and 850 °C disagree substantially with the calculated compositions, which were based on the experimental data reported in ref 16.

Bergman and Sanzharov^65^ reported a liquidus temperature of 632 °C for the composition (54 mol % Na_2_CO_3_ + 13 mol % Na_2_SO_4_ + 33 mol % K_2_CrO_4_).^65^ The calculated liquidus temperature is 648 °C.

### 5.4 Multicomponent System (NaCl + Na_2_CO_3_ + Na_2_SO_4_ + Na_2_S_2_O_7_ + Na_2_CrO_4_ + Na_2_Cr_2_O_7_ + Na_2_O + KCl + K_2_CO_3_ + K_2_SO_4_ + K_2_S_2_O_7_ + K_2_CrO_4_ + K_2_Cr_2_O_7_ + K_2_O) (Diluted in Free Oxides)

The MQMQA (described in detail in Section 2.1 of the Supporting Information) was employed to model the liquid phase of the 14-component system (diluted in free oxides). The availability of phase diagram data was limited, particularly for Na_2_CrO_4_- and K_2_CrO_4_-containing ternary common-cation subsystems, ternary reciprocal subsystems (involving Na, K, and two anions), and higher-order subsystems. Such data were available for the (Na_2_CO_3_ + Na_2_SO_4_ + Na_2_CrO_4_), (K_2_CO_3_ + K_2_SO_4_ + K_2_CrO_4_), (Na_2_SO_4_ + K_2_SO_4_ + Na_2_CrO_4_ + K_2_CrO_4_), (NaCl + KCl + Na_2_CrO_4_ + K_2_CrO_4_), and (Na_2_CO_3_ + K_2_CO_3_ + Na_2_CrO_4_ + K_2_CrO_4_) systems. For the (K_2_CO_3_ + K_2_SO_4_ + K_2_CrO_4_) system, one small ternary excess parameter was required, whereas for the (Na_2_CO_3_ + K_2_CO_3_ + Na_2_CrO_4_ + K_2_CrO_4_) system, three small ternary reciprocal parameters were needed. Satisfactory predictions were obtained for all other systems. Our present and previous evaluations^15–17^ of all ternary and reciprocal ternary subsystems showed that, typically, no, or only small, ternary or reciprocal ternary excess parameters were required to reliably reproduce the existing data.

Assuming that ternary or reciprocal ternary excess parameters can be disregarded for all ternary or ternary reciprocal subsystems for which data are missing is anticipated to permit accurate predictions of the 14-component liquid phase (diluted in free oxides).

This work, along with previous studies,^15–17^ examined various multicomponent solid solutions (hP22, oS28, oP28, hP14, oF56, and aP22); the models used are described in detail in the Supporting Information (Section 2.1 for hP22 and section 2.2 for all other solid solutions). We were able to reproduce satisfactorily the measured phase equilibria involving hP22, hP14, and oP28 in the ternary reciprocal subsystems (Na_2_SO_4_ + K_2_SO_4_ + Na_2_CrO_4_ + K_2_CrO_4_) and (NaCl + KCl + Na_2_CrO_4_ + K_2_CrO_4_), and thus no additional model parameters were included for these solid solutions. Therefore, our thermodynamic model is expected to reliably predict phase equilibria in the multicomponent system.

## 6 Conclusions

For all condensed phases of the system (NaCl + Na_2_CO_3_ + Na_2_SO_4_ + Na_2_S_2_O_7_ + Na_2_CrO_4_ + Na_2_Cr_2_O_7_ + Na_2_O + KCl + K_2_CO_3_ + K_2_SO_4_ + K_2_S_2_O_7_ + K_2_CrO_4_ + K_2_Cr_2_O_7_ + K_2_O), a thorough critical evaluation of all available phase diagram and thermodynamic data was carried out in this article and previous papers,^15–17^ and optimized model parameters have been obtained. The oxides are present in dilute amounts in the liquid phase since reactions of the type 2 A_2_CrO_4_ = A_2_Cr_2_O_7_ + A_2_O (where A = Na, K) are significantly limited at temperatures up to above the liquidus temperature.^19^ This study has taken into account all chromate and dichromate-based subsystems for which measurements (mainly phase equilibria) were available. Additionally, DSC-TGA experiments were carried out for various compositions in the common-ion binary subsystems (Na_2_CO_3_ + Na_2_CrO_4_), (K_2_CO_3_ + K_2_CrO_4_), and (Na_2_CrO_4_ + K_2_CrO_4_). In (K_2_CO_3_ + K_2_CrO_4_), a miscibility gap related to the hP22 hexagonal solid solution and some solid solubility of K_2_CO_3_ in the low-temperature allotrope K_2_CrO_4(S1)_ were observed. In (Na_2_CrO_4_ + K_2_CrO_4_), emphasis was put on the chrome-glaserite phase (hP14), and both mechanical mixtures of the two pretreated reagents and an equilibrated sample (with subsequent addition of an excess of Na_2_CrO_4_ or K_2_CrO_4_) were investigated to best identify the compositional range of hP14. Finally, the common-cation ternary mixture (55.0 mol % KCl + 28.0 mol % K_2_CO_3_ + 17.0 mol % K_2_CrO_4_) was also studied by DSC-TGA, thus permitting to confirm the existence of a ternary eutectic near this composition and around 596 °C. The data obtained both from the existing literature and from this study were reproduced within experimental error limits. The Modified Quasichemical Model in the Quadruplet Approximation (MQMQA) was employed to model both the liquid phase and the high-temperature hexagonal phase (hP22), while the Compound Energy Formalism (CEF) was used to model all other solid solutions. Using the optimized parameters obtained for the binary, common-cation ternary, and reciprocal ternary subsystems, the MQMQA allows the thermodynamic properties of the multicomponent liquid phase to be predicted with good accuracy. Our newly developed thermodynamic model can be used along with the FactSage thermochemical software^21^ to calculate phase equilibria in the multicomponent system and investigate corrosion phenomena at high temperatures, especially in the temperature range 600–950 °C. The addition to this model of the MoO_4_^2–^ and Mo_2_O_7_^2–^ anions is described in ref 20.

## Supplementary Material



## Notes

Thermodynamic data, crystal structures, and space groups for the pure compounds; thermodynamic models for the liquid phase and solid solutions (including all optimized model parameters); DSC-TGA measurements for chromate-based common-ion binary subsystems; SEM secondary electron images of equilibrated chrome-glaserite; DSC-TGA measurements for the chromate-based common-cation ternary subsystem (KCl + K_2_CO_3_ + K_2_CrO_4_); and additional calculated isoplethal sections in the (NaCl + KCl + Na_2_CrO_4_ + K_2_CrO_4_) system (PDF)
