# Supplementary material for: Thermodynamic Evaluation and Optimization of the (NaCl + Na2CO3 + Na2SO4 + Na2S2O7 + Na2CrO4 + Na2Cr2O7 + Na2O + KCl + K2CO3 + K2SO4 + K2S2O7 + K2CrO4 + K2Cr2O7 + K2O) System Involved in High-Temperature Corrosion
Source: ACS Omega. 2026 May 19;11(21):31131–55. doi: 10.1021/acsomega.6c00729 (PMC13234794; doi:10.1021/acsomega.6c00729)
Supplement: Supplementary file 1 [file ao6c00729_si_001.pdf]

SUPPORTING INFORMATION

Thermodynamic Evaluation and Optimization of the ($NaCl$ + Na_2CO_3 + Na_2SO_4 + $Na_2S_2O_7$ + Na_2CrO_4 + $Na_2Cr_2O_7$ + Na_2O + KCl + K_2CO_3 + K_2SO_4 + $K_2S_2O_7$ + K_2CrO_4 + $K_2Cr_2O_7$ + K_2O) System Involved in High-Temperature Corrosion

Sara Benalia[a,b], Fiseha Tesfaye[b], Daniel Lindberg[b,c], Leena Hupa[b], Patrice Chartrand[a], Christian Robelin[a,*]

[a] Centre for Research in Computational Thermochemistry (CRCT), Department of Chemical Engineering, Polytechnique Montréal, 3535 Queen Mary Road, Montréal (Quebec), Canada, H3V 1H8

[b] Johan Gadolin Process Chemistry Centre, Laboratory of Molecular Science and Engineering, Åbo Akademi University, Henrikinkatu 2, FI-20500 Turku, Finland

[c] Aalto University, School of Chemical Engineering, Department of Chemical and Metallurgical Engineering, FI-00076 Aalto, Finland

[*]Corresponding author (E-mail address: christian.robelin@polymtl.ca)

1. Thermodynamic data, crystal structures and space groups for the pure compounds

Table S1 provides a summary of the crystal structures and space groups of the pure salt compounds in the ($NaCl + Na_2CO_3 + Na_2SO_4 + Na_2S_2O_7 + Na_2CrO_4 + Na_2Cr_2O_7 + Na_2O + KCl + K_2CO_3 + K_2SO_4 + K_2S_2O_7 + K_2CrO_4 + K_2Cr_2O_7 + K_2O$) system.

Table S1: Crystal structures and space groups of all relevant pure salt compounds

Pure compound	Allotrope	Crystal structure	Space group	Pearson Symbol / Phase Prototype	Reference
NaCl	NaCl	Cubic	$Fm\bar{3}m$ (225)	cF8 / NaCl	FactSage (FTsalt database) [1]
KCl	KCl	Cubic	$Fm\bar{3}m$ (225)	cF8 / NaCl	FactSage (FTsalt database) [1, 2]
Na_2CO_3	Na_2CO_3 (S_1)	Monoclinic	C2/m (12)	mS24 / γ-Na_2CO_3-a	
	Na_2CO_3 (S_2)	Monoclinic	C2/m (12)	mS24 / β-Na_2CO_3-b	
	Na_2CO_3 (S_3)	Hexagonal	$P6_3/mmc$ (194)	hP22 / K_2SO_4	
K_2CO_3	K_2CO_3 (S_1)	Monoclinic	$P2_1/c$ (14)	mP24 / K_2CO_3	FactSage (FTsalt database) [1] [3]
	K_2CO_3 (S_2)	Hexagonal	$P6_3/mmc$ (194)	hP22 / K_2SO_4	
Na_2SO_4	Na_2SO_4(S_3)	Orthorhombic	Fddd (70)	oF56 / Na_2SO_4	FactSage (FTsalt database) [1], [4]
	Na_2SO_4(S_1)	Orthorhombic	Cmcm (63)	oS28 / Na_2CrO_4	
	Na_2SO_4(S_2)	Hexagonal	$P6_3/mmc$ (194)	hP22 / K_2SO_4	
$Na_2S_2O_7$	$Na_2S_2O_7$	Triclinic	$P\bar{1}$ (2)	aP22 / $Cd_2P_2O_7$	FactSage (FTsalt database) [1], [5, 6]
K_2SO_4	K_2SO_4(S_1)	Orthorhombic	Pnma (62)	oP28 / K_2SO_4	FactSage (FTsalt database) [1], [4]
	K_2SO_4(S_2)	Hexagonal	$P6_3/mmc$ (194)	hP22 / K_2SO_4	
$K_2S_2O_7$	$K_2S_2O_7$(S_1)	Monoclinic	C2/c (15)	mS44 / $K_2S_2O_7$	FactSage (FTsalt database) [1], [5]
	$K_2S_2O_7$(S_2)	-	-	-	
Na_2CrO_4	Na_2CrO_4(S_1)	Orthorhombic	Cmcm (63)	oS28 / Na_2CrO_4	[7, 8]
	Na_2CrO_4(S_2)	Hexagonal	$P6_3/mmc$ (194)	*	
$Na_2Cr_2O_7$	$Na_2Cr_2O_7$(S_1)	Triclinic	$P\bar{1}$ (2)	aP44 / $Na_2Cr_2O_7$	[9-11]
	$Na_2Cr_2O_7$(S_2)	Triclinic	$A\bar{1}$ (2)	aP22 / $Cd_2P_2O_7$	
K_2CrO_4	K_2CrO_4(S_1)	Orthorhombic	Pnma (62)	oP28 / K_2SO_4	[7, 12]
	K_2CrO_4(S_2)	Hexagonal	$P6_3/mmc$ (194)	*	
$K_2Cr_2O_7$	$K_2Cr_2O_7$(S_1)	Triclinic	$P\bar{1}$ (2)	aP44 / $K_2Cr_2O_7$	[5, 9, 13]
	$K_2Cr_2O_7$(S_2)	Monoclinic	$P2_1/c$ (14)	-	

* The corresponding Pearson symbols / prototypes for Na_2CrO_4(S_2) and K_2CrO_4(S_2) were not available to our knowledge. Thus, since these two compounds have the same crystal structure and space group as Na_2CO_3(S_3), Na_2SO_4(S_2), K_2CO_3(S_2), and K_2SO_4(S_2), they were assumed to have the same Pearson symbol / prototype phase (that is, hP22 / K_2SO_4).

2. Thermodynamic models for the liquid phase and solid solutions

2.1 Modified Quasichemical Model in the Quadruplet Approximation (MQMQA)

The Modified Quasichemical Model in the Quadruplet Approximation (MQMQA) was employed to model the liquid solution of the ($NaCl$ + Na_2CO_3 + Na_2SO_4 + $Na_2S_2O_7$ + Na_2CrO_4 + $Na_2Cr_2O_7$ + Na_2O + KCl + K_2CO_3 + K_2SO_4 + $K_2S_2O_7$ + K_2CrO_4 + $K_2Cr_2O_7$ + K_2O) system (diluted in free oxides) [14]. The cations (Na^+ and K^+) and anions (Cl^-, CO_3^{2-}, SO_4^{2-}, $S_2O_7^{2-}$, CrO_4^{2-}, $Cr_2O_7^{2-}$ and O^{2-}) are distributed over a cationic and an anionic sublattice, respectively. Using the MQMQA, the short-range order related to 1st- and 2nd-nearest-neighbour interactions within a sublattice and between sublattices can be assessed. Each quadruplet is composed of two 2nd-nearest-neighbour cations and two 2nd-nearest-neighbour anions, which are mutual 1st-nearest-neighbours. Examples of quadruplets are $Na_2(CrO_4)_2$, $NaK(CrO_4)_2$, $Na_2(Cl)(CrO_4)$ and $NaK(Cl)(CrO_4)$. Quadruplets all have a Gibbs energy and undergo random mixing, while maintaining an elemental mass balance. The equilibrium quadruplet composition or configuration is obtained by minimizing the Gibbs energy of the melt under specific conditions of temperature, pressure, and composition.

A more comprehensive description of the model can be found in reference [14]. Short-range order is relatively limited in the system investigated in this work. However, the Modified Quasichemical Model (MQM) was shown to provide a significantly improved representation compared to a random mixing model, particularly in reciprocal ternary subsystems (i.e. systems with two cations and two anions). For instance, reference [15] discusses the use of the MQM and Bragg-Williams random mixing model to describe some binary and ternary metallic liquid solutions displaying short-range ordering. The main limitations of the random mixing model were evidenced. Note that, in a liquid metal, atoms are assumed to occupy the sites of a quasi-lattice, and 1st-nearest-neighbour interactions thus need to be considered. The conclusions from reference [15] remain valid for

multicomponent common-ion molten salt systems, in which only 2nd-nearest-neighbour interactions between cations or anions need to be taken into account.

The system considered in the present work is a reciprocal molten salt solution (i.e. a solution with two or more cations, and two or more anions). In those liquid solutions, strong 1st-nearest-neighbour (cation-anion) interactions may lead to important deviations from ideal mixing. Strong 2nd-nearest-neighbour (cation-cation) short-range ordering may also occur simultaneously. The following equilibrium describes 2nd-nearest-neighbour (cation-cation) short-range ordering :

$$[\text{A-X-A}]_{\text{pair}} + [\text{B-X-B}]_{\text{pair}} = 2\,[\text{A-X-B}]_{\text{pair}};\quad \Delta g_{\text{AB/X}_2} \qquad \text{(S1)}$$

In reaction (1), A and B represent two different cations (namely Na^+ and K^+), while X is an anion. As the Gibbs energy change $\Delta g_{\text{AB/X}_2}$ becomes progressively more negative, reaction (1) is displaced to the right. Thus, [A-X-B] pairs predominate, which leads to 2nd-nearest-neighbour (cation-cation) short-range ordering. The model parameter $\Delta g_{\text{AB/X}_2}$ is defined as a function of composition through the empirical polynomial expression of equation [11] in reference [16].

First-nearest-neighbour (cation-anion) short-range ordering can take place in reciprocal salt solutions. Its extent depends on the Gibbs energy change of the following exchange reaction :

$$[\text{A-X}]_{\text{pair}} + [\text{B-Y}]_{\text{pair}} = [\text{A-Y}]_{\text{pair}} + [\text{B-X}]_{\text{pair}};\quad \Delta g_{\text{AB/XY}}^{\text{exchange}} \qquad \text{(S2)}$$

In reaction (2), A and B represent two different cations (namely Na^+ and K^+), and X and Y are two different anions. The predominance of the 1st-nearest-neighbour pairs [A-Y] and [B-X] is associated with a negative value of $\Delta g_{\text{AB/XY}}^{\text{exchange}}$. For instance, at 1250 K (which is higher than the melting points of the four pure salts), $\Delta g_{\text{NaK/Cl(CrO}_4)}^{\text{exchange}}$ for the exchange reaction KCl(l) + 0.5 Na_2CrO_4(l) ↔ NaCl(l) + 0.5 K_2CrO_4(l) is approximately -4.2 kJ/mol.

The parameters in the MQMQA are the Gibbs energies of the quadruplet-formation reactions, as illustrated by reaction (1). Additionally, there may be a small contribution

from the 2nd-nearest-neighbour anion-anion ordering. Thus, the following pair exchange reactions are also taken into account :

$$[\text{X-A-X}]_{\text{pair}} + [\text{Y-A-Y}]_{\text{pair}} = 2\ [\text{X-A-Y}]_{\text{pair}};\quad \Delta g_{\text{A}_2/\text{XY}} \quad \text{(S3)}$$

where A represents a cation (namely Na^+ or K^+), while X and Y are two different anions. Small empirical "ternary reciprocal parameters" may need to be included in the liquid model for quantitative fits. As explained previously [14], these parameters represent the Gibbs energies of formation of the ABXY quadruplets from the binary quadruplets as follows :

$$\tfrac{1}{2}(\text{ABX}_2 + \text{ABY}_2 + \text{A}_2\text{XY} + \text{B}_2\text{XY}) = 2\ (\text{ABXY});\quad \Delta g_{\text{AB/XY}} \quad \text{(S4)}$$

The model parameter $\Delta g_{\text{AB/XY}}$ is expressed as an empirical polynomial in terms of the mole fractions $x_{\text{A}_2/\text{X}_2}$, $x_{\text{A}_2/\text{Y}_2}$, $x_{\text{B}_2/\text{X}_2}$ and $x_{\text{B}_2/\text{Y}_2}$ of the unary quadruplets A_2X_2, A_2Y_2, B_2X_2 and B_2Y_2, respectively :

$$\Delta g_{\text{AB/XY}} = \Delta g^{\text{o}}_{\text{AB/XY}} + \textstyle\sum_{i\geq 1}\left[g^{i}_{\text{AB/XY(AX)}} x^{i}_{\text{A}_2/\text{X}_2} + g^{i}_{\text{AB/XY(BX)}} x^{i}_{\text{B}_2/\text{X}_2} + g^{i}_{\text{AB/XY(AY)}} x^{i}_{\text{A}_2/\text{Y}_2} + g^{i}_{\text{AB/XY(BY)}} x^{i}_{\text{B}_2/\text{Y}_2}\right] \quad \text{(S5)}$$

The empirical parameters $\Delta g^{\text{o}}_{\text{AB/XY}}$ and $g^{i}_{\text{AB/XY(AX)}}$, etc. may depend on temperature and can be derived from the optimized thermodynamic and phase diagram data in the A, B // X, Y ternary reciprocal system. These parameters are expected to be relatively small.

"Default values" of the 2nd-nearest-neighbour (cation-cation or anion-anion) coordination numbers for the ABXY quadruplets were provided through equation [23] in reference [14]. In the present work, these "default values" were employed for the Na, K // SO_4, CrO_4; Na, K // CO_3, CrO_4 and Na, K // Cl, CrO_4 ternary reciprocal systems.

The 2nd-nearest-neighbour coordination numbers are model parameters. As explained in references [16, 17], in the common-ion binary systems A, B // X and A // X, Y, the maximum short-range ordering compositions are determined by the ratios $\left(Z^{\text{B}}_{\text{AB/X}_2}/Z^{\text{A}}_{\text{AB/X}_2}\right)$ and $\left(Z^{\text{Y}}_{\text{A}_2/\text{XY}}/Z^{\text{X}}_{\text{A}_2/\text{XY}}\right)$, respectively. $Z^{i}_{\text{AB/X}_2}$ and $Z^{i}_{\text{A}_2/\text{XY}}$ represent the 2nd-nearest-neighbour coordination numbers of the ion *i* when all *i* exist in ABX_2 and A_2XY quadruplets, respectively. The 2nd-nearest-neighbour coordination numbers are assigned appropriate absolute values to best reproduce the available phase diagram and

thermodynamic data. Note that the chosen absolute values may be different from the experimental values.

All common-cation ternary subsystems within the main system ($NaCl + Na_2CO_3 + Na_2SO_4 + Na_2S_2O_7 + Na_2CrO_4 + Na_2Cr_2O_7 + KCl + K_2CO_3 + K_2SO_4 + K_2S_2O_7 + K_2CrO_4 + K_2Cr_2O_7$) need to be designated as either "symmetric" or "asymmetric" [16]. The liquid model includes the Na^+ and K^+ cations, as well as the following main anions: Cl^-, CO_3^{2-}, SO_4^{2-}, $S_2O_7^{2-}$, CrO_4^{2-}, and $Cr_2O_7^{2-}$. All anions are classified into two distinct chemical groups based on their valence: group 1 consists solely of Cl^- whereas group 2 consists of CO_3^{2-}, SO_4^{2-}, $S_2O_7^{2-}$, CrO_4^{2-} and $Cr_2O_7^{2-}$. In the present study, common-cation ternary subsystems involving NaCl or KCl (such as ($KCl + K_2CO_3 + K_2CrO_4$)) have two anions in the same group while Cl^- belongs to another group. Thus, a Kohler-Toop-like (asymmetric) interpolation method is employed with Cl^- as the asymmetric component. For common-cation ternary subsystems without NaCl or KCl (such as ($K_2CO_3 + K_2SO_4 + K_2CrO_4$)), all three anions are in the same group, and a Kohler-like (symmetric) interpolation method is therefore employed. The parameters $\Delta g_{AB/X_2}$ (reaction (1)) and $\Delta g_{A_2/XY}$ (reaction (3)) are expanded, through optimization with available experimental data for the A, B // X and A // X, Y common-ion binary systems, respectively, as empirical polynomials in χ_{ij} and χ_{ji}. Once an interpolation method has been selected for each ternary common-ion subsystem, the composition variables χ_{ij} and χ_{ji} are defined unambiguously [16].
Additional terms may be introduced for common-cation ternary subsystems (AX + AY + AZ), in which A represents Na^+ or K^+, and X, Y, and Z are three different anions. These terms express the effect of the third component, AZ, on the energy of formation $\Delta g_{A_2/XY}$ of the binary quadruplet A_2XY. The corresponding empirical ternary parameter $g^{ijk}_{A/XY(Z)}$ can be obtained by optimizing the thermodynamic and phase diagram data in the common-cation ternary system A // X, Y, Z.

The Gibbs energy of the solution can be expressed as follows:

$$G = \sum n_{ij/kl}\, g_{ij/kl} - T\,\Delta S^{config} \qquad (S6)$$

where $n_{ij/kl}$ represents the number of moles of the various quadruplets (unary, binary, and reciprocal), $g_{ij/kl}$ represents the Gibbs energy of these quadruplets, and the configurational entropy of mixing ΔS^{config} considers quasichemical short-range ordering. The latter is determined from a random distribution of all quadruplets over "quadruplet positions". Note that there is no accurate mathematical expression for this distribution. However, an approximate expression for ΔS^{config} was provided in reference [14].

As explained in reference [18], the MQMQA has undergone several improvements. The expression of the configurational entropy has been refined, and slight modifications have been made to the interpolation expression of the Gibbs energy excess parameters in reciprocal systems. Additionally, the parameter ζ, defined in equation [17] of reference [14] and associated with the ratio between the 2nd-nearest-neighbour and 1st-nearest-neighbour coordination numbers for a species *i*, is no longer considered to be constant. These recent modifications have been made to address the limitation of the previous model, in the case of the Gibbs energy change $\Delta g_{AB/XY}^{exchange}$ of reaction (2) becoming very negative. This issue was discussed in paragraph 3.3.4 of reference [19]. The parameter ζ has been defined previously [14] as :

$$\zeta = 2(Z/z) \qquad \text{(S7)}$$

where *z* and *Z* represent the 1st-nearest-neighbour (cation-anion) and 2nd-nearest-neighbour (cation-cation or anion-anion) coordination numbers, respectively. In the reciprocal system Li^+, Na^+, K^+, Mg^{2+}, Ca^{2+} // F^-, Cl^-, the parameter ζ has been assigned a value of 2.4 [20].
ζ refers to the number of quadruplets emanating from, or containing, a 1st-nearest-neighbour [A-X] pair [14]. The refined version of the MQMQA employs the notation $\zeta_{A/X}$, which is defined as follows :

$$\zeta_{A/X} = 2Z^{A}_{A_2/X_2}Z^{X}_{A_2/X_2}\Big/\left(Z^{A}_{A_2/X_2}+Z^{X}_{A_2/X_2}\right) \qquad \text{(S8)}$$

In equation (8), $Z^{i}_{A_2/X_2}$ denotes the 2nd-nearest-neighbour coordination number of the ion *i* (*i* = A, X) when all *i* exist in A_2X_2 quadruplets. The values of the 2nd-nearest-neighbour coordination numbers and of the newly optimized model parameters for the liquid phase of the Na^+, K^+ // Cl^-, CO_3^{2-}, SO_4^{2-}, $S_2O_7^{2-}$, CrO_4^{2-}, $Cr_2O_7^{2-}$, O^{2-} reciprocal system (diluted in

free oxides) are given in Tables S2 and S3, respectively. For the reciprocal system Na^+, K^+ // Cl^-, CO_3^{2-}, SO_4^{2-}, $S_2O_7^{2-}$, CrO_4^{2-}, $Cr_2O_7^{2-}$, O^{2-}, the values of $\zeta_{A/X}$ (equation (8)) are :

$$\zeta_{A/X} = 4; \quad \zeta_{A/Cl} = 6 \qquad (S9)$$

where A = Na^+, K^+; and X = CO_3^{2-}, SO_4^{2-}, $S_2O_7^{2-}$, CrO_4^{2-}, $Cr_2O_7^{2-}$, O^{2-}.

Table S2: 2nd-nearest-neighbour coordination numbers for the quadruplets ABXY of the newly optimized systems of the Na^+, K^+ // Cl^-, CO_3^{2-}, SO_4^{2-}, $S_2O_7^{2-}$, CrO_4^{2-}, $Cr_2O_7^{2-}$, O^{2-} liquid phase

A	B	X	Y	$Z^{A}_{AB/XY}$	$Z^{B}_{AB/XY}$	$Z^{X}_{AB/XY}$	$Z^{Y}_{AB/XY}$
Na	Na	Cl	Cl	6	6	6	6
K	K	Cl	Cl	6	6	6	6
Na	Na	CO_3	CO_3	3	3	6	6
K	K	CO_3	CO_3	3	3	6	6
Na	Na	SO_4	SO_4	3	3	6	6
K	K	SO_4	SO_4	3	3	6	6
Na	Na	S_2O_7	S_2O_7	3	3	6	6
K	K	S_2O_7	S_2O_7	3	3	6	6
Na	Na	CrO_4	CrO_4	3	3	6	6
K	K	CrO_4	CrO_4	3	3	6	6
Na	Na	Cr_2O_7	Cr_2O_7	3	3	6	6
K	K	Cr_2O_7	Cr_2O_7	3	3	6	6
Na	Na	O	O	3	3	6	6
K	K	O	O	3	3	6	6
Na	Na	Cl	CrO_4	4	4	4	8
K	K	Cl	CrO_4	4	4	4	8
Na	Na	CO_3	CrO_4	3	3	6	6
Na	Na	SO_4	CrO_4	3	3	6	6
K	K	CO_3	CrO_4	3	3	6	6
K	K	SO_4	CrO_4	3	3	6	6
Na	K	CrO_4	CrO_4	3	3	6	6

K	K	Cl	Cr_2O_7	4	4	6	6
K	K	CrO_4	Cr_2O_7	3	3	6	6
Na	Na	CrO_4	Cr_2O_7	3	3	6	6
Na	K	Cr_2O_7	Cr_2O_7	3	3	6	6

The 2nd-nearest-neighbour coordination numbers for the ABXY quadruplets of the Na^+, K^+ // Cl^-, CO_3^{2-}, SO_4^{2-}, $S_2O_7^{2-}$liquid were given previously in the work of Lindberg et al [3, 4, 21] , and they were used directly in the present work.

Table S3: Model parameters optimized in the present work for the Na^+, K^+ // Cl^- , CO_3^{2-}, SO_4^{2-}, $S_2O_7^{2-}$, CrO_4^{2-}, $Cr_2O_7^{2-}$, O^{2-} liquid phase (diluted in free oxides)

System	Model parameter (J/mol)
($NaCl + Na_2CrO_4$)	$\Delta g_{Na_2/(Cl)(CrO_4)} = -250.0 - 1200.0\ \chi_{(CrO_4)(Cl)}$
($Na_2CO_3 + Na_2CrO_4$)	$\Delta g_{Na_2/(CO_3)(CrO_4)} = 350.0 + 750.0\chi_{(CrO_4)(CO_3)}$
($K_2CO_3 + K_2CrO_4$)	$\Delta g_{K_2/(CO_3)(CrO_4)} = 1,000.0$
($Na_2CrO_4 + K_2CrO_4$)	$\Delta g_{NaK/(CrO_4)_2} = -1,419.6$
($KCl + K_2Cr_2O_7$)	$\Delta g_{K_2/(Cl)(Cr_2O_7)} = -1,400.0$
($K_2CO_3 + K_2SO_4 + K_2CrO_4$)	$g^{101}_{K/(CrO_4)(CO_3)[SO_4]} = -9,000.0$
($Na_2CO_3 + K_2CO_3 + Na_2CrO_4$ + K_2CrO_4)	$\Delta g_{NaK/(CO_3)(CrO_4)} = -900.0 - 9,500.0x_{Na_2/(CO_3)_2} - 9,000.0x_{K_2/(CrO_4)_2}$

2.2 Compound Energy Formalism (CEF)

In this work, various solid solutions were modeled in the Na^+, K^+ // Cl^-, CO_3^{2-}, SO_4^{2-}, $S_2O_7^{2-}$, CrO_4^{2-}, $Cr_2O_7^{2-}$ main system. These are described in detail in Table S4, and all optimized model parameters are given. The Na^+ , K^+ // CO_3^{2-}, SO_4^{2-}, CrO_4^{2-} subsystem exhibits a hexagonal solid solution (hP22) at high temperatures, with complete mutual miscibility over the entire composition range.

This reciprocal solid solution was modeled using the MQMQA [14] with two sublattices, where the cations reside on the cationic sublattice and the anions reside on the anionic sublattice. Lindberg et al [4] previously modeled the high-temperature ($Na_2SO_4 + K_2SO_4$)

hexagonal solid solution using the Compound Energy Formalism (CEF) [22-24], in which the Na^+ and K^+ cations were randomly distributed on the cationic sublattice. Approximately at the same time, Coursol et al [25] modeled the high-temperature (Na_2SO_4 + $CaSO_4$) solid solution (with $CaSO_4$ in limited amounts) using the MQM in the pair approximation, which takes account of 2nd-nearest-neighbour (cation-cation) short-range-order. This solid solution displays a maximum evidenced by the available experimental data and well reproduced by the MQM. The MQM is more suited than the CEF to model complex solid solutions such as (Na_2SO_4 + K_2SO_4) dissolving divalent cation sulfates (such as $CaSO_4$, $ZnSO_4$ and $PbSO_4$). In a recent work [26], the MQM was retained, thus permitting us to add $ZnSO_4$ and $PbSO_4$ as solutes in the high-temperature (Na_2SO_4 + K_2SO_4) hexagonal solid solution. For the sake of consistency, the MQMQA was used in the present work to model the hP22 solid solution (with Na^+ and K^+ as the cations, and CO_3^{2-}, SO_4^{2-} and CrO_4^{2-} as the anions). In addition, several low-temperature solid solutions displaying limited solubility were considered. The CEF was used to model those.

Three sublattices were required for the chrome-glaserite phase $K_3Na(CrO_4)_2$, which exhibits significant non-stoichiometry at temperatures above room temperature [27, 28]. In all other cases, only two sublattices were necessary. For instance, let us consider the oF56 solid solution which consists of the orthorhombic low-temperature allotrope $Na_2SO_{4(S3)}$ dissolving Na_2CrO_4, K_2SO_4 and K_2CrO_4. The sublattice structure $(Na^+, K^+)_2(SO_4^{2-}, CrO_4^{2-})$ is used, where Na^+ and K^+ reside on the cationic sublattice C while SO_4^{2-} and CrO_4^{2-} reside on the anionic sublattice A. The molar Gibbs energy of the solution is given by the following expression :

$$G_m = y^C_{Na^+} y^A_{SO_4^{2-}} G^\circ_{Na^+:SO_4^{2-}} + y^C_{Na^+} y^A_{CrO_4^{2-}} G^\circ_{Na^+:CrO_4^{2-}} + y^C_{K^+} y^A_{SO_4^{2-}} G^\circ_{K^+:SO_4^{2-}} + y^C_{K^+} y^A_{CrO_4^{2-}} G^\circ_{K^+:CrO_4^{2-}} + 2RT\left(y^C_{Na^+} \ln(y^C_{Na^+}) + y^C_{K^+} \ln(y^C_{K^+})\right) + RT\left(y^A_{SO_4^{2-}} \ln(y^A_{SO_4^{2-}}) + y^A_{CrO_4^{2-}} \ln(y^A_{CrO_4^{2-}})\right) + G^E \quad (S10)$$

The first four terms give the reference Gibbs energy of the solution, where $y^C_{Na^+}$ and $y^C_{K^+}$ are the site fractions of Na^+ and K^+ on the cationic sublattice C, and $y^A_{SO_4^{2-}}$ and $y^A_{CrO_4^{2-}}$ are the site fractions of SO_4^{2-} and CrO_4^{2-} on the anionic sublattice A. The standard molar Gibbs energies of the "end-member" components Na_2SO_4, K_2SO_4, Na_2CrO_4 and K_2CrO_4 are

represented by $\overset{\circ}{G}_{Na^+:SO_4^{2-}}$, $\overset{\circ}{G}_{K^+:SO_4^{2-}}$, $\overset{\circ}{G}_{Na^+:CrO_4^{2-}}$, and $\overset{\circ}{G}_{K^+:CrO_4^{2-}}$, respectively. The fifth and sixth terms in equation (10) account for the ideal entropy of mixing, assuming a random distribution of the cations on the cationic sublattice and of the anions on the anionic sublattice (Temkin type, [29]). The final term refers to the molar excess Gibbs energy and has the following general expression :

$$G^E = y^C_{Na^+} y^C_{K^+} y^A_{SO_4^{2-}} L_{Na^+,K^+:\,SO_4^{2-}} + y^C_{Na^+} y^C_{K^+} y^A_{CrO_4^{2-}} L_{Na^+,K^+:\,CrO_4^{2-}}$$
$$+ y^C_{Na^+} y^A_{SO_4^{2-}} y^A_{CrO_4^{2-}} L_{Na^+:\,SO_4^{2-},CrO_4^{2-}} + y^C_{K^+} y^A_{SO_4^{2-}} y^A_{CrO_4^{2-}} L_{K^+:\,SO_4^{2-},CrO_4^{2-}}$$
$$+ y^C_{Na^+} y^C_{K^+} y^A_{SO_4^{2-}} y^A_{CrO_4^{2-}} L_{Na^+,K^+:\,SO_4^{2-},CrO_4^{2-}} \quad \text{(S11)}$$

The first four terms are interaction parameters in the four common-ion binary subsystems, and the last term is a reciprocal interaction parameter. The L factors can be made temperature-dependent and also composition-dependent, where Redlich-Kister terms $L^i_{i\geq 0}(y_A - y_B)^i$ as a function of site fractions are usually used. Note that no excess Gibbs energy term was required in the present work to model the oF56 solid solution.

Table S4 : Description and list of optimized model parameters for the various solid solutions in the Na$^+$, K$^+$ // Cl$^-$, CO_3^{2-}, SO_4^{2-}, $S_2O_7^{2-}$, CrO_4^{2-}, $Cr_2O_7^{2-}$ main system modeled in the present work

**Hexagonal solid solution hP22**
High-temperature allotropes $Na_2CO_{3(S3)}$, $K_2CO_{3(S2)}$, $Na_2SO_{4(S2)}$, $K_2SO_{4(S2)}$, $Na_2CrO_{4(S2)}$ and $K_2CrO_{4(S2)}$ with mutual solubility
$P6_3/mmc$ space group
Sublattice structure: $(Na^+, K^+)_2(CO_3^{2-}, SO_4^{2-}, CrO_4^{2-})$<br>Use of the MQMQA
The Gibbs energies of the "end-members" are:<br>$\overset{\circ}{g}_{Na_2CO_3} = \overset{\circ}{g}_{Na_2CO_{3(S3)}}$<br>$\overset{\circ}{g}_{K_2CO_3} = \overset{\circ}{g}_{K_2CO_{3(S2)}}$<br>$\overset{\circ}{g}_{Na_2SO_4} = \overset{\circ}{g}_{Na_2SO_{4(S2)}}$<br>$\overset{\circ}{g}_{K_2SO_4} = \overset{\circ}{g}_{K_2SO_{4(S2)}}$<br>$\overset{\circ}{g}_{Na_2CrO_4} = \overset{\circ}{g}_{Na_2CrO_{4(S2)}}$<br>$\overset{\circ}{g}_{K_2CrO_4} = \overset{\circ}{g}_{K_2CrO_{4(S2)}}$

The 2nd-nearest-neighbour coordination number of each cationic species is 3.0, and that of each anionic species is 6.0. Thus, all $\zeta_{A/X}$ are equal to 4.0 (with A = Na^+, K^+ and X = CO_3^{2-}, SO_4^{2-}, CrO_4^{2-}).<br><br>The following interaction parameters for (Na_2CO_3 + Na_2SO_4), (K_2CO_3 + K_2SO_4), (Na_2CO_3 + K_2CO_3), (Na_2SO_4 + K_2SO_4) and Na, K // CO_3, SO_4 were obtained previously:<br>$\Delta g_{Na_2/(CO_3)(SO_4)} = 1{,}106.7 + 1{,}060.0\, Y_{CO_3}$ (J/mol) [3] (This is a Bragg-Williams type term and Y_{CO_3} is the equivalent site fraction of CO_3)<br>$\Delta g_{K_2/(CO_3)(SO_4)} = 1{,}571.7 + 105.0\, Y_{CO_3}$ (J/mol) [3]<br>$\Delta g_{NaK/(CO_3)_2} = 4{,}935.0 - 2{,}928.2\, Y_K$ (J/mol) [3]<br>$\Delta g_{NaK/(SO_4)_2} = 3{,}040.4 + (2{,}370.9 - 3.1241 \times T)\, Y_K$ (J/mol) [3, 4]<br>$\Delta g_{NaK/(CO_3)(SO_4)} = 1{,}401.3$ (J/mol) [3]<br><br>The following CrO_4-based interaction parameters were obtained in the present work:<br>$\Delta g_{Na_2/(CO_3)(CrO_4)} = 8{,}000.0 - 1{,}800.0\, Y_{CrO_4}$ (J/mol)<br>$\Delta g_{K_2/(CO_3)(CrO_4)} = 5{,}600.0 - 650.0\, Y_{CrO_4}$ (J/mol)[‡]<br>$\Delta g_{Na_2/(SO_4)(CrO_4)} = 800.0$ (J/mol)<br>$\Delta g_{K_2/(SO_4)(CrO_4)} = 1{,}000.0 - 500.0\, Y_{SO_4}$ (J/mol)<br>$\Delta g_{NaK/(CrO_4)_2} = 2{,}700.0 + (2{,}000.0 - 2.4500 \times T) Y_K$ (J/mol)<br><br>[‡] : This model parameter was reported previously [30] as $\Delta g_{K_2/(CO_3)(CrO_4)} = 5{,}600.0 - 800.0\, Y_{CrO_4}$ (J/mol), but was then revised owing to the adjustment of the thermodynamic properties of K_2CrO_4.
**Solid solution oS28**<br>Orthorhombic low-temperature allotropes $Na_2CrO_{4(S1)}$ and $Na_2SO_{4(S1)}$ mutually soluble, and dissolving K_2CrO_4, Na_2CO_3, K_2SO_4 and K_2CO_3<br>Cmcm space group<br>Sublattice structure: $(Na^+, K^+)_2(SO_4^{2-}, CrO_4^{2-}, CO_3^{2-})$<br><br>The Gibbs energies of the "end-members" are:<br>$\overset{\circ}{g}_{Na_2CrO_4} = \overset{\circ}{g}_{Na_2CrO_{4(S1)}}$<br>$\overset{\circ}{g}_{Na_2SO_4} = \overset{\circ}{g}_{Na_2SO_{4(S1)}}$<br>$\overset{\circ}{g}_{Na_2CO_3} = \overset{\circ}{g}_{Na_2CO_{3(S2)}} + 7{,}112.8$ (J/mol) [3]<br>$\overset{\circ}{g}_{K_2CrO_4} = \overset{\circ}{g}_{K_2CrO_{4(S1)}} + (33{,}472.0 - 7.7404 \times T)$ (J/mol)<br>$\overset{\circ}{g}_{K_2SO_4} = \overset{\circ}{g}_{K_2SO_{4(S1)}} + 33{,}472.0$ (J/mol) [3]<br>$\overset{\circ}{g}_{K_2CO_3} = \overset{\circ}{g}_{K_2CO_{3(S1)}} + 4{,}184.0$ (J/mol) [3]<br><br>The following Redlich-Kister interaction parameter for (Na_2CO_3 + Na_2SO_4) was obtained previously:<br>$L^0_{Na/(CO_3)(SO_4)} = 3{,}347.2$ (J/mol) [3]<br><br>The following CrO_4-based Redlich-Kister interaction parameters were obtained in the present work:<br>$L^0_{Na/(CrO_4)(CO_3)} = 100{,}000.0$ (J/mol)

$L^0_{Na/(CrO_4)(SO_4)} = 3{,}000.0$ (J/mol)

Solid solution oP28

Orthorhombic low-temperature allotropes $K_2CrO_{4(S1)}$ and $K_2SO_{4(S1)}$ mutually soluble, and dissolving Na_2CrO_4, K_2CO_3, Na_2SO_4 and Na_2CO_3

Pnma space group

Sublattice structure: $(K^+, Na^+)_2(SO_4^{2-}, CrO_4^{2-}, CO_3^{2-})$

The Gibbs energies of the "end-members" are:

$\overset{\circ}{g}_{K_2CrO_4} = \overset{\circ}{g}_{K_2CrO_{4(S1)}}$

$\overset{\circ}{g}_{K_2SO_4} = \overset{\circ}{g}_{K_2SO_{4(S1)}}$

$\overset{\circ}{g}_{K_2CO_3} = \overset{\circ}{g}_{K_2CO_{3(S1)}} + 209.2$ (J/mol) [3]

$\overset{\circ}{g}_{Na_2CrO_4} = \overset{\circ}{g}_{Na_2CrO_{4(S1)}} + (22{,}384.4 + 11.9244 \times T)$ (J/mol)‡

$\overset{\circ}{g}_{Na_2SO_4} = \overset{\circ}{g}_{Na_2SO_{4(S1)}} + (29{,}288.0 - 20.9200 \times T)$ (J/mol) [3]

$\overset{\circ}{g}_{Na_2CO_3} = \overset{\circ}{g}_{Na_2CO_{3(S1)}} + 20{,}920.0$ (J/mol) [3]

The following Redlich-Kister interaction parameters for (K_2CO_3 + K_2SO_4) were obtained previously:

$L^0_{K/(CO_3)(SO_4)} = 3{,}765.6$ (J/mol) [3]

$L^1_{K/(CO_3)(SO_4)} = -2{,}008.3$ (J/mol) [3]

The following CrO_4-based Redlich-Kister interaction parameters were obtained in the present work:

$L^0_{K/(CO_3)(CrO_4)} = 17{,}500.0$ (J/mol)

$L^1_{K/(CO_3)(CrO_4)} = 3{,}000.0$ (J/mol)

$L^0_{K/(CrO_4)(SO_4)} = 1{,}800.0$ (J/mol)

‡ : This model parameter was reported previously [30] as $\overset{\circ}{g}_{Na_2CrO_4} = \overset{\circ}{g}_{Na_2CrO_{4(S1)}} + (13{,}388.8 + 2.7196 \times T)$ (J/mol), but was then revised owing to the adjustment of the thermodynamic properties of Na_2CrO_4 and K_2CrO_4.

Glaserite solid solution hP14

Solid $K_3Na(SO_4)_2$ and $K_3Na(CrO_4)_2$ mutually soluble, and dissolving Na^+

P$\bar{3}$m1 space group

Sublattice structure: $(K^+, Na^+)_3 (Na^+) (SO_4^{2-}, CrO_4^{2-})_2$

The Gibbs energies of the "end-members" are:

$\overset{\circ}{g}_{K_3Na(SO_4)_2} = 1.5 \times \overset{\circ}{g}_{K_2SO_{4(S1)}} + 0.5 \times \overset{\circ}{g}_{Na_2SO_{4(S2)}} - 10{,}878.4 + 9.7906 \times T$ (J/mol) [4]

$\overset{\circ}{g}_{Na_3Na(SO_4)_2} = 2 \times \overset{\circ}{g}_{Na_2SO_{4(S2)}} + 16{,}736.0 - 10.7110 \times T$ (J/mol) [4]

$\overset{\circ}{g}_{K_3Na(CrO_4)_2} = 1.5 \times \overset{\circ}{g}_{K_2CrO_{4(S1)}} + 0.5 \times \overset{\circ}{g}_{Na_2CrO_{4(S1)}} - 17{,}154.4 + 9.4140 \times T$ (J/mol)

$\overset{\circ}{g}_{Na_3Na(CrO_4)_2} = 2 \times \overset{\circ}{g}_{Na_2CrO_{4(S1)}} + 12{,}552.0 - 8.1588 \times T$ (J/mol)‡

‡ : This model parameter was reported previously [30] as $\overset{\circ}{g}_{Na_3Na(CrO_4)_2} = 2 \times \overset{\circ}{g}_{Na_2CrO_{4(S1)}} + 12{,}970.4 - 8.1588 \times T$ (J/mol), but was then revised owing to the adjustment of the thermodynamic properties of Na_2CrO_4 and K_2CrO_4.

Solid solution oF56

Orthorhombic low-temperature allotrope $Na_2SO_{4(S3)}$ dissolving Na_2CrO_4, K_2SO_4 and K_2CrO_4

Fddd space group

Sublattice structure: $(Na^+, K^+)_2(SO_4^{2-}, CrO_4^{2-})$

The Gibbs energies of the "end-members" are:

$\overset{\circ}{g}_{Na_2SO_4} = \overset{\circ}{g}_{Na_2SO_{4(S3)}}$

$\overset{\circ}{g}_{K_2SO_4} = \overset{\circ}{g}_{K_2SO_{4(S1)}} + 66{,}944.0$ (J/mol)

$\overset{\circ}{g}_{Na_2CrO_4} = \overset{\circ}{g}_{Na_2CrO_{4(S1)}} + 4{,}100.3$ (J/mol)

$\overset{\circ}{g}_{K_2CrO_4}^{*} = \overset{\circ}{g}_{K_2CrO_{4(S1)}} + 66{,}944.0$ (J/mol)

Solid solution aP22

Triclinic allotropes $Na_2S_2O_7$ and $Na_2Cr_2O_{7(S2)}$ mutually soluble, and dissolving $K_2S_2O_7$ and $K_2Cr_2O_7$

$A\bar{1}$ space group

Sublattice structure: $(Na^+, K^+)_2(S_2O_7^{2-}, Cr_2O_7^{2-})$

The Gibbs energies of the "end-members" are:

$\overset{\circ}{g}_{Na_2S_2O_7} = \overset{\circ}{g}_{Na_2S_2O_{7(S)}}$

$\overset{\circ}{g}_{K_2S_2O_7} = \overset{\circ}{g}_{K_2S_2O_{7(S2)}} + 29{,}288.0$ (J/mol) [4]

$\overset{\circ}{g}_{Na_2Cr_2O_7} = \overset{\circ}{g}_{Na_2Cr_2O_{7(S2)}}$

$\overset{\circ}{g}_{K_2Cr_2O_7} = \overset{\circ}{g}_{K_2Cr_2O_{7(S2)}} + 10{,}460.0 + 2.0920 \times T$ (J/mol)‡

‡ : This model parameter was reported previously [30] as $\overset{\circ}{g}_{K_2Cr_2O_7} = \overset{\circ}{g}_{K_2Cr_2O_{7(S2)}} + 7{,}531.2 + 5.6484 \times T$ (J/mol), but was then revised owing to the adjustment of the thermodynamic properties of $Na_2Cr_2O_7$ and $K_2Cr_2O_7$.

Solid solution aP44

Triclinic low-temperature allotropes $Na_2Cr_2O_{7(S1)}$ and $K_2Cr_2O_{7(S1)}$ mutually soluble

$P\bar{1}$ space group

Sublattice structure: $(Na^+, K^+)_2(Cr_2O_7^{2-})$

The Gibbs energies of the "end-members" are:

$\overset{\circ}{g}_{Na_2Cr_2O_7} = \overset{\circ}{g}_{Na_2Cr_2O_{7(S1)}}$

$\overset{\circ}{g}_{K_2Cr_2O_7} = \overset{\circ}{g}_{K_2Cr_2O_{7(S1)}}$

The following Redlich-Kister interaction parameter was obtained in the present work:

$L^0_{NaK/Cr_2O_7} = 20{,}000.0$ (J/mol)

**Solid solution $K_2Cr_2O_7$(s.s)**<br>Monoclinic high-temperature allotrope $K_2Cr_2O_{7(S2)}$ dissolving $Na_2Cr_2O_7$<br>$P2_1/c$ space group<br>Sublattice structure: $(K^+, Na^+)_2(Cr_2O_7^{2-})$<br>The Gibbs energies of the "end-members" are:<br>$\mathring{g}_{K_2Cr_2O_7} = \mathring{g}_{K_2Cr_2O_{7(S2)}}$<br>$\mathring{g}_{Na_2Cr_2O_7} = \mathring{g}_{Na_2Cr_2O_{7(S2)}} + 11{,}296.8 + 5.0208 \times T$ (J/mol)‡<br><br>‡ : This model parameter was reported previously [30] as $\mathring{g}_{Na_2Cr_2O_7} = \mathring{g}_{Na_2Cr_2O_{7(S2)}} + 11{,}296.8 + 3.8911 \times T$ (J/mol), but was then revised owing to the adjustment of the thermodynamic properties of $Na_2Cr_2O_7$ and $K_2Cr_2O_7$.
**Rocksalt solid solution cF8**<br>Cubic NaCl and KCl mutually soluble<br>$Fm\bar{3}m$ space group<br><br>The Gibbs energies of the "end-members" are:<br>$\mathring{g}_{NaCl} = \mathring{g}_{NaCl(S)}$<br>$\mathring{g}_{KCl} = \mathring{g}_{KCl(S)}$<br><br>The following Redlich-Kister interaction parameters for (NaCl + KCl) were obtained previously:<br>$L^0_{NaK/Cl} = 15{,}972.0 + 32.7960 \times T - 5.5930 \times T \times ln(T)$(J/mol) [31]<br>$L^1_{NaK/Cl} = 1{,}639.0$ (J/mol) [31]

* The "end-member" K_2CrO_4 was added to the oF56 solid solution. Its Gibbs energy in the hypothetical orthorhombic / Fddd crystal structure is that of $K_2CrO_{4(S1)}$ augmented by a positive Gibbs energy. The latter was assumed to be identical to that for the "end-member" K_2SO_4 (that is, 66,944.0 J/mol), which was previously obtained by Lindberg et al in an unpublished work. The "end-member" K_2CrO_4 is only required for calculations in the Na, K // SO_4, CrO_4 ternary reciprocal system and higher-order systems.

3. Chromate-based common-ion binary subsystems

DSC-TGA results are presented below for several compositions in the (Na_2CO_3 + Na_2CrO_4), (K_2CO_3 + K_2CrO_4), and (Na_2CrO_4 + K_2CrO_4) common-ion binary subsystems.

3.1 The ($Na_2CO_3 + Na_2CrO_4$) system

Table S5: DSC-TGA results for ($Na_2CO_3 + Na_2CrO_4$) binary mixtures (2nd and 3rd heating/cooling cycles only)

Mol% of Na_2CrO_4	T_{Onset} (°C) 2nd heating	T_{Onset} (°C) 3rd heating	T_{Peak} (°C) 2nd heating	T_{Peak} (°C) 3rd heating	Mass Change (%)
**5**	668.6	668.6	847.0	847.0	**-0.24**
**10**	668.9	668.9	829.6	829.6	**-0.13**
**90**	343.8	343.8	754.3	754.3	**-0.38**
**92.5**	376.0	376.0	774.8	774.9	**-0.50**
**95**	379.2	379.6	780.6	780.6	**-0.41**

3.2 The ($K_2CO_3 + K_2CrO_4$) system

Table S6: DSC-TGA results for ($K_2CO_3 + K_2CrO_4$) binary mixtures (2nd and 3rd heating/cooling cycles only)

Mol% of K_2CrO_4	T_{Onset} (°C) 2nd heating	T_{Onset} (°C) 3rd heating	T_{Peak} (°C) 2nd heating	T_{Peak} (°C) 3rd heating	Mass Change (%)
**40**			682.4*<br>826.7	683.6*<br>827.5	**-2.07**
**60**	661.9	662.0	826.3<br>861.6	826.3<br>861.6	**-2.39**
**80**	661.4	661.4	921.0	920.2	**-1.27**
**90**	662.5	662.5	947.1	947.5	**-0.97**

* These temperature values were taken at the maximum of the peak (and not at the onset of the peak) since these were very low intensity peaks.

3.3 The ($Na_2CrO_4 + K_2CrO_4$) system

Table S7: DSC-TGA results for mechanical mixtures of pre-treated reagents $Na_2CrO_4.4H_2O$ and K_2CrO_4 in the ($Na_2CrO_4 + K_2CrO_4$) binary subsystem (2nd and 3rd heating/cooling cycles only)

Mol% of K_2CrO_4	T_{Onset} (°C) 2nd heating	T_{Onset} (°C) 3rd heating	T_{Peak} (°C) 2nd heating	T_{Peak} (°C) 3rd heating	Mass Change (%)
**35**	569.0	568.9	232.1*	231.3*	**-1.27**
			752.9	752.7	
**45**	537.8	530.6	331.8*	331.5*	**1.03****
			773.6	772.9	
	597.2	597.5			
**55**	534.1	537.8	330.0*	329.5*	**-0.35**
			802.2	809.0	
	606.6	606.7			
**65**	528.5	533.3	326.3*	327.5*	**0.87****
			841.4	843.7	
	590.9	590.9			
**70**	586.2	586.6	527.5*	528.7*	**-0.63**
			860.9	860.5	
**75**	554.1	554.4	278.2*	277.7*	**-0.71**
			528.2*	528.2*	
			881.4	873.9	

* These temperature values were taken at the maximum of the peak (and not at the onset of the peak) since these were very low intensity peaks.

** There was a small mass gain associated with oxidation due to impurity traces of oxygen in Ar(g).

The binary compositions listed in Table S8 were investigated by DSC-TGA; they were obtained by adding an excess of Na_2CrO_4 or K_2CrO_4 to a sample of chrome-glaserite with the nominal composition of (45 mol% Na_2CrO_4 + 55 mol% K_2CrO_4) equilibrated at 400°C for three weeks.

Table S8: DSC-TGA results for binary mixtures obtained by addition of an excess of Na_2CrO_4 or K_2CrO_4 to an equilibrated sample with the nominal composition (45 mol% Na_2CrO_4 + 55 mol% K_2CrO_4) (1st, 2nd and 3rd heating/cooling cycles only, and all temperatures in °C)

Mol% of K_2CrO_4	T_{Onset} 1st heating	T_{Onset} 2nd heating	T_{Onset} 3rd heating	T_{Peak} 1st heating	T_{Peak} 2nd heating	T_{Peak} 3rd heating	Mass Change (%)
**40**		601.2	601.1	374.1*	332.3*		**-0.35**
				497.8*	516.1*	520.6*	
				778.4	779.8	778.4	
**65**		605.2	605.2	556.7*			**-0.31**
				809.9	816.4	825.3	
**72**		599.5	600.3	557.2*			**-0.41**
				827.8	835.4	836.6	
**73**	552.5	594.2	594.0	841.0	839.2	841.3	**-0.63**

* These temperature values were taken at the maximum of the peak (and not at the onset of the peak) since these were very low intensity peaks.

4. Scanning Electron Microscopy (SEM) and Energy Dispersive X-ray Spectroscopy (EDS)

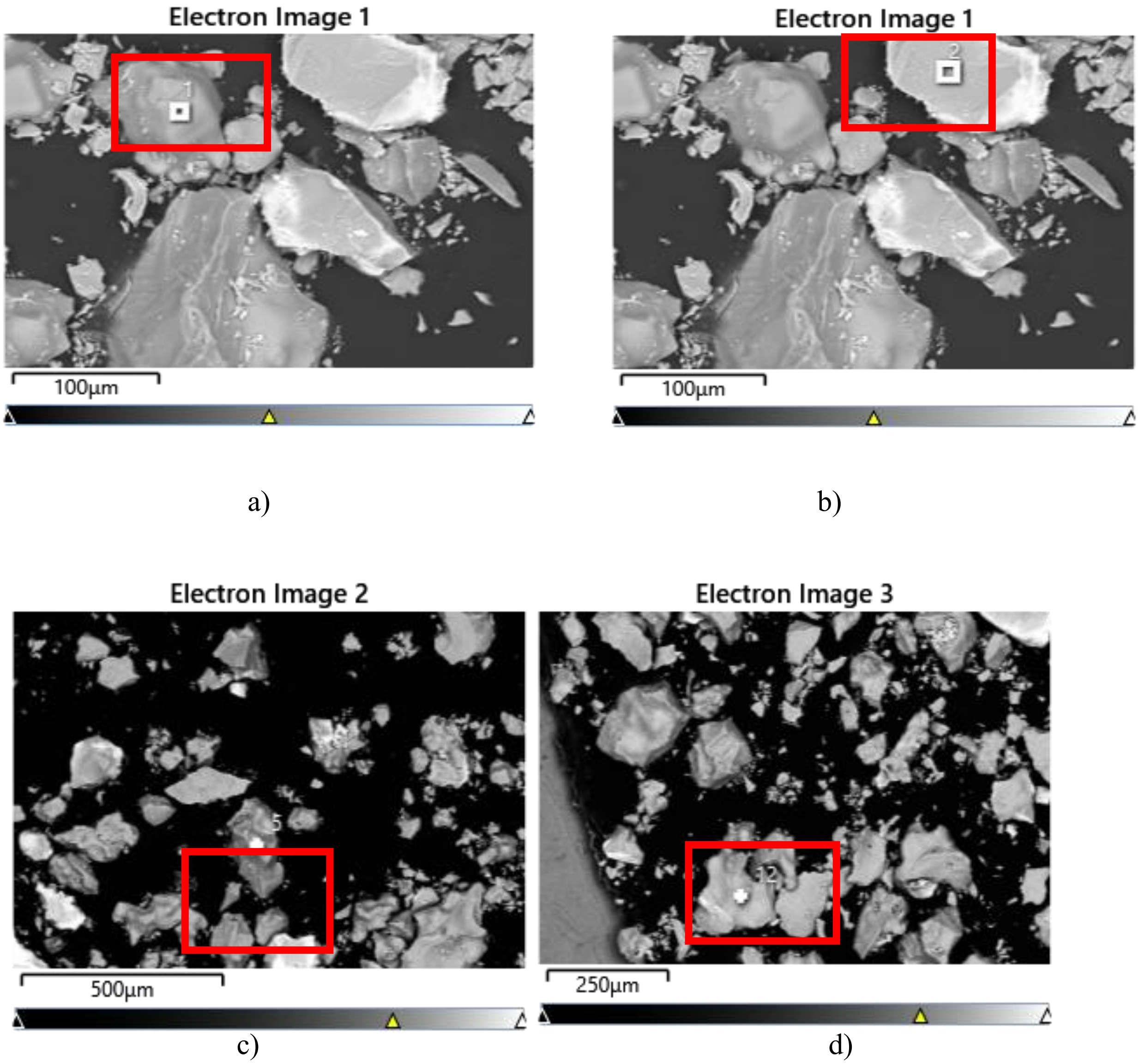

Figure S1: SEM secondary electron images of the equilibrated chrome-glaserite sample with the nominal composition of (45 mol% Na_2CrO_4 + 55 mol% K_2CrO_4) : a) spectrum 1, b) spectrum 2, c) spectrum 5, and d) spectrum 12

5. Chromate-based common-cation ternary subsystems

5.1 The ($KCl + K_2CO_3 + K_2CrO_4$) system

Table S9: DSC-TGA results for a ($KCl + K_2CO_3 + K_2CrO_4$) common-cation ternary mixture (2nd and 3rd heating/cooling cycles only)

Composition	T_{Peak} (°C) 2nd heating	T_{Peak} (°C) 3rd heating	Mass Change (%)
(55.0 mol% KCl + 28.0 mol% K_2CO_3 + 17.0 mol% K_2CrO_4)	595.5	596.3	**-2.34**

6. Chromate-based ternary reciprocal subsystems

6.1 The ($NaCl + KCl + Na_2CrO_4 + K_2CrO_4$) system

Calculations are presented below for four of the fifteen isoplethal sections measured by Bergman and Trunin [32] using the visual-polythermal method. All experimental data (●) were extracted graphically from the smoothed liquidus projection of these authors.

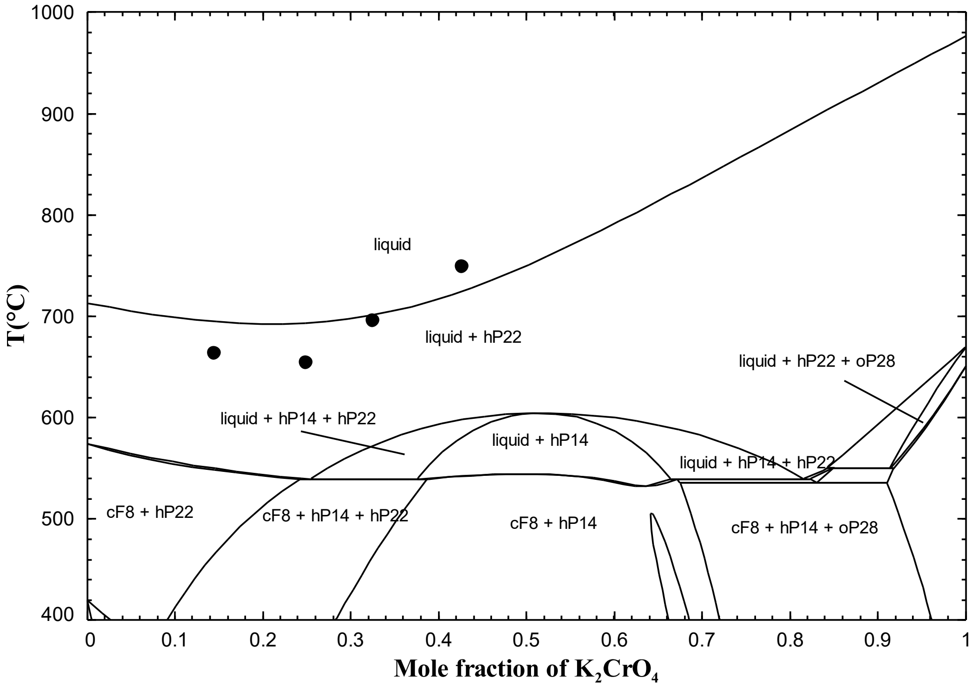

Figure S2: Calculated isoplethal section in the (NaCl + KCl + Na_2CrO_4 + K_2CrO_4) system ((Na_2CrO_4)$_{0.897}$(Na_2Cl_2)$_{0.103}$-K_2CrO_4, section I)

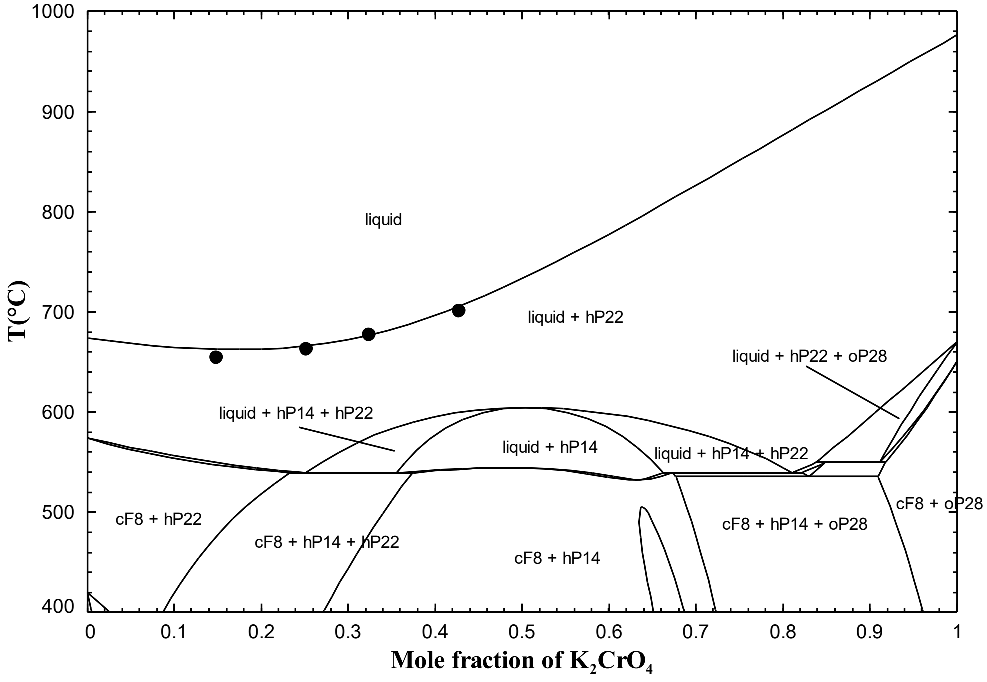

Figure S3: Calculated isoplethal section in the (NaCl + KCl + Na_2CrO_4 + K_2CrO_4) system ((Na_2CrO_4)$_{0.846}$(Na_2Cl_2)$_{0.154}$-K_2CrO_4, section II)

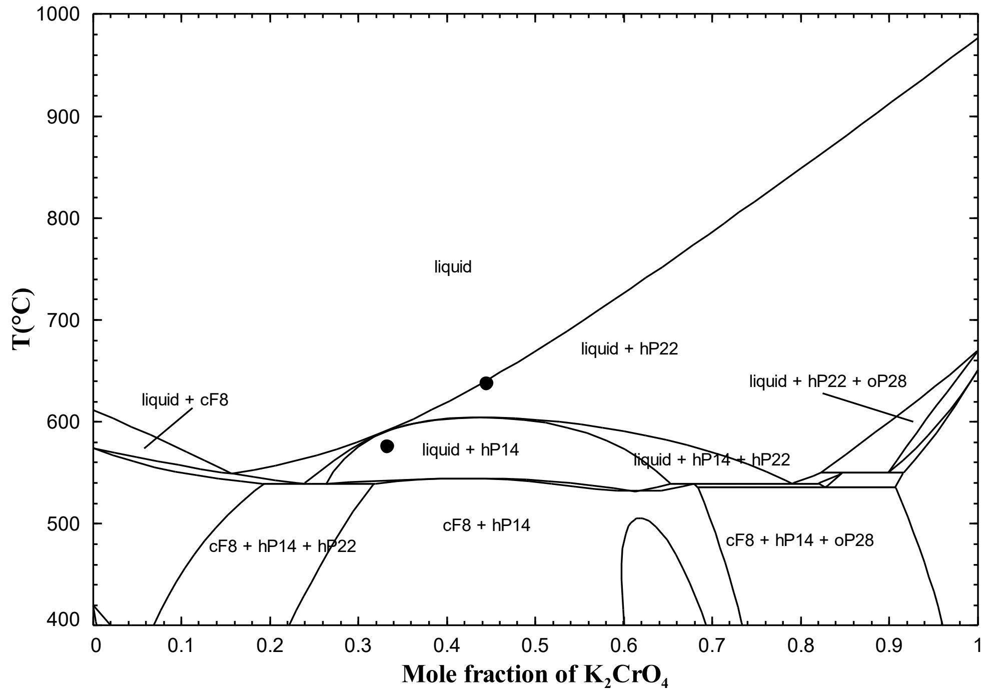

Figure S4: Calculated isoplethal section in the ($NaCl + KCl + Na_2CrO_4 + K_2CrO_4$) system ($(Na_2CrO_4)_{0.644}(Na_2Cl_2)_{0.356}$-$K_2CrO_4$, section IV)

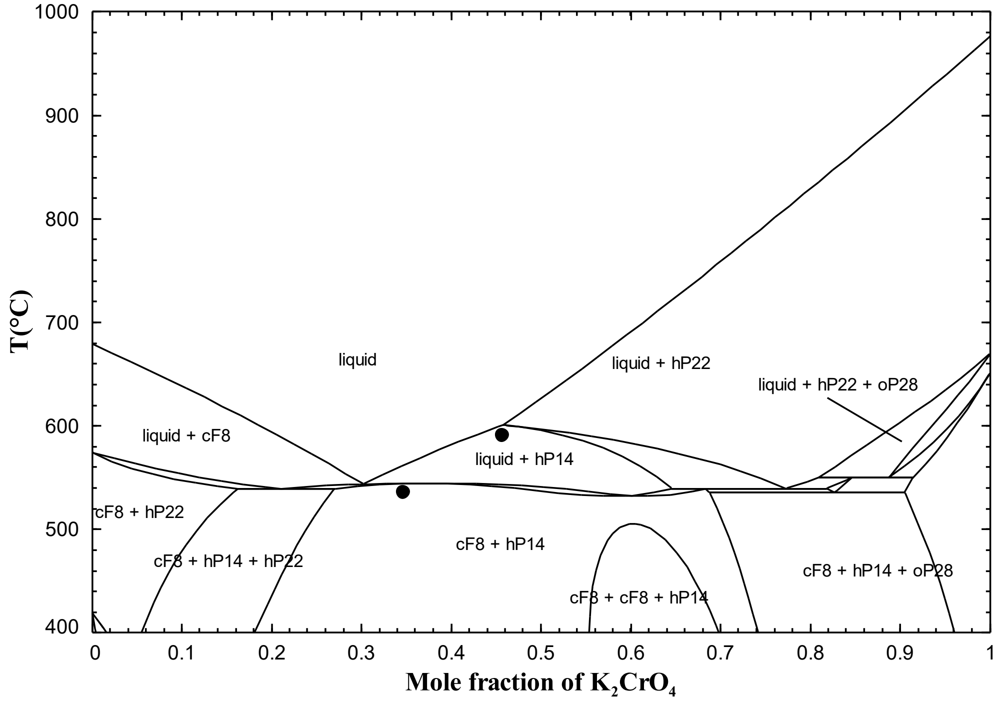

Figure S5: Calculated isoplethal section in the ($NaCl + KCl + Na_2CrO_4 + K_2CrO_4$) system ($(Na_2CrO_4)_{0.497}(Na_2Cl_2)_{0.503}$-$K_2CrO_4$, section V)

References

[1] C. W. Bale *et al.*, FactSage thermochemical software and databases, 2010–2016," *Calphad,* vol. 54, pp. 35-53, 2016.

[2] γ-Na_2CO_3 ($Na_2[CO_3]$ rt) Crystal Structure: Datasheet from "PAULING FILE Multinaries Edition - 2022" in Springer Materials (https://materials.springer.com/isp/crystallographic/docs/sd_2042290). Springer-Verlag Berlin Heidelberg & Material Phases Data System (MPDS), Switzerland National Institute for Materials Science (NIMS), Japan. [Online]. Available: https://materials.springer.com/isp/crystallographic/docs/sd_2042290

[3] D. Lindberg, R. Backman, and P. Chartrand, "Thermodynamic evaluation and optimization of the (Na_2CO_3 + Na_2SO_4 + Na_2S + K_2CO_3 + K_2SO_4 + K_2S) system," *Journal of Chemical Thermodynamics,* vol. 39, no. 6, pp. 942-960, 2007.

[4] D. Lindberg, R. Backman, and P. Chartrand, "Thermodynamic evaluation and optimization of the (Na_2SO_4 + K_2SO_4 + $Na_2S_2O_7$ + $K_2S_2O_7$) system," *Journal of Chemical Thermodynamics,* vol. 38, no. 12, pp. 1568-1583, 2006.

[5] S. R. Hall and B. McMahon, *International tables for crystallography, definition and exchange of crystallographic data*. Springer 2005, p. 594.

[6] *$Na_2S_2O_7$ ($Na_2[S_2O_7]$) Crystal Structure: Datasheet from "PAULING FILE Multinaries Edition – 2022" in SpringerMaterials (https://materials.springer.com/isp/crystallographic/docs/sd_1838862).* Springer-Verlag Berlin Heidelberg & Material Phases Data System (MPDS), Switzerland & National Institute for Materials Science (NIMS), Japan. [Online]. Available: https://materials.springer.com/isp/crystallographic/docs/sd_1838862

[7] S. Benalia *et al.*, "Critical Evaluation and Calorimetric Study of the Thermodynamic Properties of Na_2CrO_4, K_2CrO_4, Na_2MoO_4, K_2MoO_4, Na_2WO_4, and K_2WO_4," *Journal of Physical and Chemical Reference Data,* vol. 52, no. 4, 2023.

[8] *Na_2CrO_4 ($Na_2[CrO_4]$ rt) Crystal Structure: Datasheet from "PAULING FILE Multinaries Edition – 2022" in SpringerMaterials (https://materials.springer.com/isp/crystallographic/docs/sd_1001365).* Springer-Verlag Berlin Heidelberg & Material Phases Data System (MPDS), Switzerland & National Institute for Materials Science (NIMS), Japan. [Online]. Available: https://materials.springer.com/isp/crystallographic/docs/sd_1001365

[9] S. Benalia, P. Chartrand, and C. Robelin, "Critical Evaluation of the Thermodynamic Properties of $Na_2Cr_2O_7$, $K_2Cr_2O_7$, $Na_2Mo_2O_7$, $K_2Mo_2O_7$, $Na_2W_2O_7$, and $K_2W_2O_7$," *Journal of Physical and Chemical Reference Data,* vol. 52, no. 4, 2023.

[10] *β-$Na_2Cr_2O_7$ ($Na_2[Cr_2O_7]$ rt) Crystal Structure: Datasheet from "PAULING FILE Multinaries Edition – 2022" in SpringerMaterials (https://materials.springer.com/isp/crystallographic/docs/sd_1401687).* Springer-Verlag Berlin Heidelberg & Material Phases Data System (MPDS), Switzerland & National Institute for Materials Science (NIMS), Japan. [Online]. Available: https://materials.springer.com/isp/crystallographic/docs/sd_1401687

[11] *α-$Na_2Cr_2O_7$ ($Na_2[Cr_2O_7]$ ht) Crystal Structure: Datasheet from "PAULING FILE Multinaries Edition – 2022" in SpringerMaterials (https://materials.springer.com/isp/crystallographic/docs/sd_1100126).* Springer-Verlag Berlin Heidelberg & Material Phases Data System (MPDS), Switzerland & National Institute for Materials Science (NIMS), Japan. [Online]. Available: https://materials.springer.com/isp/crystallographic/docs/sd_1100126

[12] *K_2CrO_4 ($K_2[CrO_4]$ tar) Crystal Structure: Datasheet from "PAULING FILE Multinaries Edition – 2022" in SpringerMaterials (https://materials.springer.com/isp/crystallographic/docs/sd_0377606).*

Springer-Verlag Berlin Heidelberg & Material Phases Data System (MPDS), Switzerland & National Institute for Materials Science (NIMS), Japan. [Online]. Available: https://materials.springer.com/isp/crystallographic/docs/sd_0377606

[13] *$K_2Cr_2O_7$ ($K_2[Cr_2O_7]$ rt) Crystal Structure: Datasheet from "PAULING FILE Multinaries Edition – 2022" in SpringerMaterial (https://materials.springer.com/isp/crystallographic/docs/sd_1322711).* Springer-Verlag Berlin Heidelberg & Material Phases Data System (MPDS), Switzerland & National Institute for Materials Science (NIMS), Japan. [Online]. Available: https://materials.springer.com/isp/crystallographic/docs/sd_1322711

[14] A. D. Pelton, P. Chartrand, and G. Eriksson, "The modified quasi-chemical model: Part IV. Two-sublattice quadruplet approximation," *Metallurgical and Materials Transactions A,* vol. 32, no. 6, pp. 1409-1416, 2001.

[15] A. D. Pelton and Y.-B. Kang, "Modeling short-range ordering in solutions," *International Journal of Materials Research,* vol. 98, no. 10, pp. 907-917, 2007.

[16] A. D. Pelton and P. Chartrand, "The modified quasi-chemical model: Part II. Multicomponent solutions," *Metallurgical and Materials Transactions A,* vol. 32, no. 6, pp. 1355-1360, 2001.

[17] A. D. Pelton, S. Degterov, G. Eriksson, C. Robelin, and Y. Dessureault, "The modified quasichemical model I-binary solutions," *Metallurgical and Materials Transactions B,* vol. 31, no. 4, pp. 651-659, 2000.

[18] G. Lambotte and P. Chartrand, "Thermodynamic optimization of the (Na_2O + SiO_2 + NaF + SiF_4) reciprocal system using the Modified Quasichemical Model in the Quadruplet Approximation," *Journal of Chemical Thermodynamics,* vol. 43, no. 11, pp. 1678-1699, 2011.

[19] P. Chartrand, "New thermodynamic models for liquid solutions," Copyright © 2023 American Chemical Society (ACS). All Rights Reserved., 2000.

[20] P. Chartrand and A. D. Pelton, "Thermodynamic evaluation and optimization of the Li, Na, K, Mg, Ca//F, Cl reciprocal system using the modified quasi-chemical model," *Metallurgical and Materials Transactions A,* vol. 32, no. 6, pp. 1417-1430, 2001.

[21] D. Lindberg, R. Backman, and P. Chartrand, "Thermodynamic evaluation and optimization of the (NaCl + Na_2SO_4 + Na_2CO_3 + KCl + K_2SO_4 + K_2CO_3) system," *Journal of Chemical Thermodynamics,* vol. 39, no. 7, pp. 1001-1021, 2007.

[22] B. Sundman and J. Ågren, "A regular solution model for phases with several components and sublattices, suitable for computer applications," *Journal of physics and chemistry of solids,* vol. 42, no. 4, pp. 297-301, 1981.

[23] M. Hillert, B. Jansson, and B. Sundman, "Application of the Compound-Energy Model to Oxide Systems/Anwendung des „Compound-Energy "-Modells auf Oxidsysteme," *International Journal of Materials Research,* vol. 79, no. 2, pp. 81-87, 1988.

[24] M. Hillert, "The compound energy formalism," *Journal of Alloys and Compounds,* vol. 320, no. 2, pp. 161-176, 2001.

[25] P. Coursol, A. D. Pelton, P. Chartrand, and M. Zamalloa, "The $CaSO_4$-Na_2SO_4-CaO Phase Diagram," *Canadian Metallurgical Quarterly,* vol. 44, no. 4, pp. 537-546, 2005.

[26] L. Jin, D. Lindberg, Y. Tsuchiyama, and C. Robelin, "A thermodynamic model for high temperature corrosion applications: The (Na_2SO_4 + K_2SO_4 + $ZnSO_4$ + $PbSO_4$) system," *Chemical Engineering Science,* vol. 260, p. 117847, 2022.

[27] W. Eysel, "Crystal-Chemistry of System Na_2SO_4-K_2SO_4-K_2CrO_4-Na_2CrO_4 and of Glaserite Phase," *American Mineralogist,* vol. 58, no. 7-8, pp. 736-747, 1973.

[28] A. Goldberg, W. Eysel, T. Halm, A. Goldberg, W. Eysel, and T. Hahn, "Phase diagram and crystallography of the system Na_2CrO_4-K_2CrO_4," *Neues Jahrbuch für Mineralogie-Monatshefte,* vol. H. 6, pp. 241-252, 1973.

[29] M. Temkin, "Mixtures of fused salts as ionic solutions," *Acta physicochimica URSS,* vol. 20, p. 411, 1945.

[30] S. Benalia, F. Tesfaye, D. Lindberg, L. Hupa, P. Chartrand, and C. Robelin, "Thermodynamic Model for High Temperature Corrosion Applications: The (NaCl + Na_2CO_3 + Na_2SO_4 + $Na_2S_2O_7$ + Na_2CrO_4 + $Na_2Cr_2O_7$ + Na_2MoO_4 + $Na_2Mo_2O_7$ + Na_2O+ KCl + K_2CO_3 + K_2SO_4 + $K_2S_2O_7$ + K_2CrO_4 + $K_2Cr_2O_7$ + K_2MoO_4 + $K_2Mo_2O_7$ + K_2O) System," *Industrial & Engineering Chemistry Research,* vol. 62, no. 49, pp. 21397-21427, 2023.

[31] J. M. Sangster and A. D. Pelton, "Phase diagrams and thermodynamic properties of the 70 binary alkali halide systems having common ions," *Journal of Physical and Chemical Reference Data,* vol. 16, pp. 509-561, 1987.

[32] A. G. Bergman and A. S. Trunin, "The Na, K ll Cl, CrO_4 system," *Zhurnal Neorganicheskoi Khimii* vol. 12, no. 7, pp. 1966-8, 1967.
